# Integrative Proteomics and Metabolomics Analysis Reveals the Role of Small Signaling Peptide Rapid Alkalinization Factor 34 (RALF34) in Cucumber Roots

**DOI:** 10.3390/ijms24087654

**Published:** 2023-04-21

**Authors:** Julia Shumilina, Alexey S. Kiryushkin, Nadezhda Frolova, Valeria Mashkina, Elena L. Ilina, Vera A. Puchkova, Katerina Danko, Svetlana Silinskaya, Evgeny B. Serebryakov, Alena Soboleva, Tatiana Bilova, Anastasia Orlova, Elizaveta D. Guseva, Egor Repkin, Katharina Pawlowski, Andrej Frolov, Kirill N. Demchenko

**Affiliations:** 1 Saint Petersburg State University, 199034 Saint Petersburg, Russia; 2 Laboratory of Cellular and Molecular Mechanisms of Plant Development, Komarov Botanical Institute, Russian Academy of Sciences, 197022 Saint Petersburg, Russia; 3 Laboratory of Analytical Biochemistry and Biotechnology, Timiryazev Institute of Plant Physiology, Russian Academy of Sciences, 127276 Moscow, Russiafrolov@ifr.moscow (A.F.); 4 Department of Ecology, Environment and Plant Sciences, Stockholm University, 10691 Stockholm, Sweden

**Keywords:** *Cucumis sativus* (cucumber), small signaling peptides, RALF34, root development, overexpression, hairy root transformation, tandem mass spectrometry, proteomics, metabolomics

## Abstract

The main role of RALF small signaling peptides was reported to be the alkalization control of the apoplast for improvement of nutrient absorption; however, the exact function of individual RALF peptides such as RALF34 remains unknown. The Arabidopsis RALF34 (*At*RALF34) peptide was proposed to be part of the gene regulatory network of lateral root initiation. Cucumber is an excellent model for studying a special form of lateral root initiation taking place in the meristem of the parental root. We attempted to elucidate the role of the regulatory pathway in which RALF34 is a participant using cucumber transgenic hairy roots overexpressing *CsRALF34* for comprehensive, integrated metabolomics and proteomics studies, focusing on the analysis of stress response markers. *Cs*RALF34 overexpression resulted in the inhibition of root growth and regulation of cell proliferation, specifically in blocking the G2/M transition in cucumber roots. Based on these results, we propose that *CsRALF34* is not part of the gene regulatory networks involved in the early steps of lateral root initiation. Instead, we suggest that *Cs*RALF34 modulates ROS homeostasis and triggers the controlled production of hydroxyl radicals in root cells, possibly associated with intracellular signal transduction. Altogether, our results support the role of RALF peptides as ROS regulators.

## 1. Introduction

Peptides can act as signaling molecules [1,2,3,4]. Small signaling peptides participate in various aspects of the plant life cycle, including development, nutrient availability signaling, and response to environmental cues, similar to canonical phytohormones [5,6,7,8,9,10].

Peptides of the rapid alkalinization factor (RALF) family were isolated from tobacco leaves [11] and have been studied for over twenty years as plant developmental regulators [12]. RALF peptides were discovered to be factors causing rapid alkalization of the medium [11]. They regulate various processes such as root growth and development [13,14,15], root nodule formation [16], fertilization [17,18], fruit ripening [19], and plant–pathogen interactions [20,21].

The Arabidopsis RALF family consists of 37 members [15]. The Arabidopsis RALF34 (*At*RALF34) peptide was suggested to be involved in the gene regulatory network controlling lateral root initiation [14]. *AtRALF34*, regulated by auxin and likely by ethylene, is expressed before the first asymmetric divisions of pericycle cells at the xylem pole [14], as well as before the expression of *AtGATA23*, the earliest known marker for lateral root initiation, which controls founder cell identity [22]. Arabidopsis RALF34 serves as a negative regulator of lateral root initiation and probably prevents initiation in close proximity to existing primordia [14], acting in a non-cell-autonomous manner similarly to CLE peptides [23,24].

Previously, 17 members of the RALF protein family were identified in cucumber [25]. One peptide, *Cs*RALF34, was proposed to be the ortholog of *At*RALF34. *CsRALF34*, the gene encoding this peptide, is expressed in most plant organs, i.e., in the roots, hypocotyls, cotyledons, leaves, male and female flowers, as well as fruits. The expression of *CsRALF34* in roots is not regulated by auxin but seems to be partially inducible by ethylene [25]. Relative transcript levels of *CsRALF34* were downregulated in roots treated with 10 μM 1-aminocylopropane-1-carboxylic acid (ACC, a precursor of ethylene) but were not affected by treatment with 1 μM 2-aminoisobutyric acid (AIB, an inhibitor of ethylene biosynthesis).

In the roots of Arabidopsis and cucumber, the expression of *RALF34* started in the protoxylem cells before any visible sign of lateral root initiation [14,25]. In contrast to Arabidopsis, in cucumber, this process began very close to the initial cells in the apical part of the root meristem [25]. In the cucumber pericycle, *RALF34* promoter activity was first detected in the single lateral root founder cell before auxin response maxima were established in the two pericycle cells, which gave rise to lateral root primordia. *RALF34* promoter activity was found in lateral root primordia until lateral root emergence. Recently, we have shown that in cucumber roots, RALF34 was synthesized in the xylem and pericycle cells, then transported and accumulated in the apoplast and cell walls of the cortex cells in the basal part of the meristem [25].

Signal transduction involving RALF peptides and their receptors includes dozens of different signaling pathways and signal molecules. There are numerous reviews analyzing and systematizing individual components of this complex signaling machinery [12,26,27,28,29,30]. However, each molecular player of the RALF signaling pathway is not clearly understood, nor are the accompanying alterations in root metabolism. Although the dynamics of root cell metabolism can efficiently be characterized by conventional biochemical methods [31,32], in the most efficient and straightforward way, this can be addressed by post-genomic research methods in terms of the proteomics and metabolomics methodological platforms [33,34,35]. A combination of these two bioanalytical techniques is universally recognized as the best suited to analyze the metabolic shifts during root development [36].

To gain deeper insight into the changes in the cucumber proteome associated with the overexpression of *CsRALF34*, a bottom-up shotgun proteomics approach can be employed. This analytical strategy relies on the limited digestion of total protein isolates with site-specific proteases combined with subsequent analysis of the resulting proteolytic peptides using nano-flow liquid chromatography-mass spectrometry (nanoLC-MS) [37]. This method is free from any bias for protein pI, hydrophobicity, and molecular weight when combined with highly efficient phenol-based extraction procedures and MS-friendly digestion techniques [38] that enable an analysis of the total proteome [39]. Therefore, shotgun bottom-up proteomics are the method of choice for the characterization of plant proteome responses, i.e., dynamics of the abundance of individual proteins, in the context of the biochemical and physiological changes accompanying developmental processes [40,41].

However, metabolic shifts can be caused not only by changes in protein levels but also by the modulation of enzyme activity [42]. The latter effects cannot be recognized at the proteome level. Thus, the dynamics of corresponding metabolites need to be addressed by metabolomics. Nevertheless, analysis of primary and secondary metabolites can efficiently complement the proteomics data towards a better insight into the biochemical response accompanying lateral root formation [43]. The overlay of the protein and metabolite dynamics can provide a generalized overview of the metabolic pathways which are up- or downregulated under experimental conditions [44]. This information is also important for the identification of the signaling pathways involved, although the patterns of post-translational enzyme modifications are informative in this context as well [45]. A comprehensive study of the proteome, metabolism, and biochemical markers of stress is required for a thorough analysis of the effect of RALF34.

In this study, *CsRALF34* overexpression in cucumber roots was used to elucidate the role of this gene at the early stages of lateral root initiation in the apical meristem of the parental root. The effect of *CsRALF34* overexpression was analyzed in detail at the molecular level using proteomics and metabolomics techniques.

## 2. Results

### 2.1. Overexpression of CsRALF34 Does Not Affect Lateral Root Initiation in Cucumber

**Figure 1:**
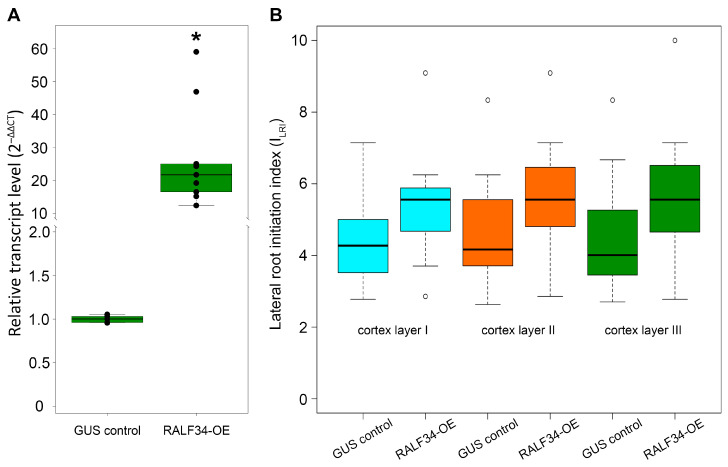
Overexpression of *Cucumis sativus RALF34* in transgenic roots. (**A**) Expression of *CsRALF34* in control roots (GUS control) and in roots with an overexpression construct (RALF34-OE). RT-qPCR analysis was performed using RNA isolated from the control group (mix of roots) and from individual transgenic roots. Statistical analysis using Wilcoxon’s signed-rank test showed a significant increase (*, *p* < 0.001) of expression levels in the overexpression group compared with the control. The *y*-axis indicates the relative transcript level (2^–ΔΔCT^ method). (**B**) The Lateral Root Initiation Index in three outer cortical layers of control roots (GUS control) and of roots overexpressing *RALF34* (RALF34-OE). Statistical analysis using Wilcoxon’s signed-rank test showed no significant differences (*p* > 0.05) in the I_LRI_ of individual roots in the overexpression group compared with control roots.

Transgenic hairy root systems overexpressing *CsRALF34* were used to clarify the role of *CsRALF34* during the early steps of lateral root initiation in the apical meristem of cucumber parental roots (Figure 1). Hairy root transformation of cucumber was performed with a control construct (*p35S::gusA*) and the overexpression construct (*p35S::CsRALF34*), respectively. Expression levels of *CsRALF34* were analyzed using RT-qPCR in individual transgenic roots, showing that relative expression levels of *CsRALF34* were upregulated compared with control roots by 12- to 59-fold (Figure 1A). The Lateral Root Initiation Index (I_LRI_) was determined for the control group (GUS control) and for roots overexpressing *CsRALF34* (RALF34-OE) (Figure 1B). The I_LRI_ of roots overexpressing *CsRALF34* showed no statistically significant differences from the I_LRI_ of control roots.

### 2.2. Treatment of Cucumber Roots with Synthetic CsRALF34 Affects Root Growth but Not Lateral Root Initiation

**Figure 2:**
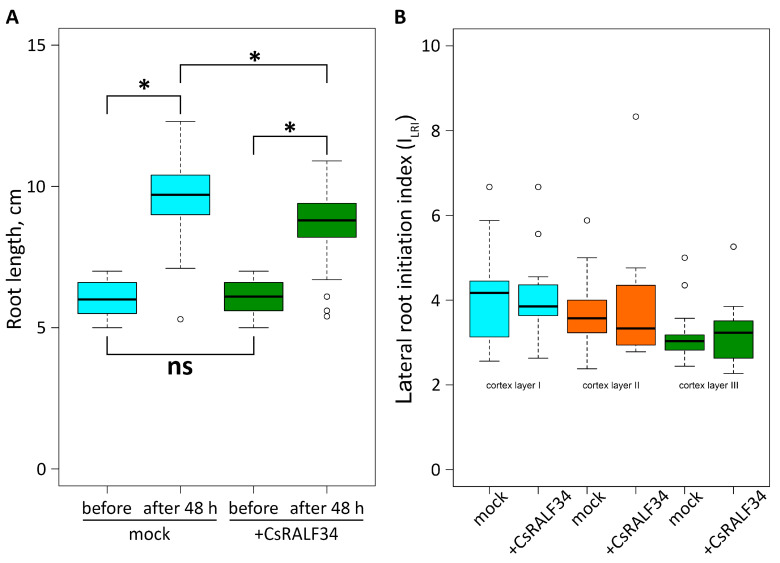
Comparison of *Cucumis sativus* roots treated with 2 μM synthetic *Cs*RALF34 peptide for 48 h. (**A**) Comparison of average root length between the control group (mock, *n* = 100) and treated roots (*+Cs*RALF34, *n* = 100). Statistical analysis using Wilcoxon’s signed-rank test showed no significant differences in root length before treatment (ns) and significant differences in root length (*, *p* < 0.001) after treatment. (**B**) Comparison of the Lateral Root Initiation Index in three outer cortical layers. Statistical analysis using Wilcoxon’s signed-rank test showed no significant differences (*p* > 0.05) between the I_LRI_ values of control roots (mock, *n* = 14) and treated roots (+*Cs*RALF34; *n* = 10).

Wild-type cucumber roots were treated with 2 μM *Cs*RALF34 peptide for 48 h (Figure 2A). Before the start of the experiment, no statistically significant differences in root length were observed between cucumber seedlings in the control and in the peptide-treated group; the average initial root length was 6.03 cm for the control and 6.11 cm for the peptide-treated group. After 48 h of treatment, the average root length of the control group had increased from 6.03 cm to 9.71 cm (root length increment of 3.68 cm). In the peptide-treated group, it had increased from 6.11 cm to 8.75 cm (root length increment 2.64 cm). The root length increment of the peptide-treated group was 1.04 cm lower than that of the control group, a statistically significant difference (*p* < 0.001). The I_LRI_ was determined for both groups (Figure 2B); there was no statistically significant difference between the I_LRI_ of peptide-treated roots and control group roots.

### 2.3. Overexpression of CsRALF34 Does Not Affect the Expression of CsGATA14, CsGATA24, and CsE2F/DP Genes

**Figure 3:**
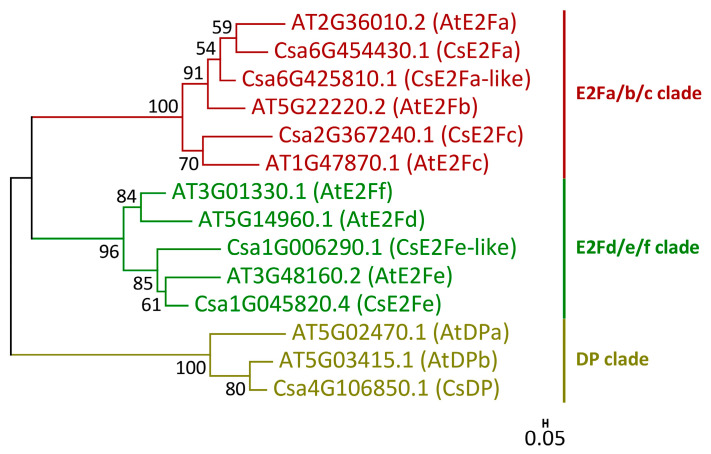
Phylogenetic tree of E2F/DP proteins from *Arabidopsis thaliana* and *Cucumis sativus*. The three different clades of E2F/DP proteins are presented in different colors. Gene ID prefixes: AT, *A. thaliana* based on the Arabidopsis Information Resource; Csa, *C. sativus* cv. Chinese Long v2 based on the Cucurbit Genomics Database v1. Scale bar: 0.05 amino acid substitutions per site.

**Figure 4:**
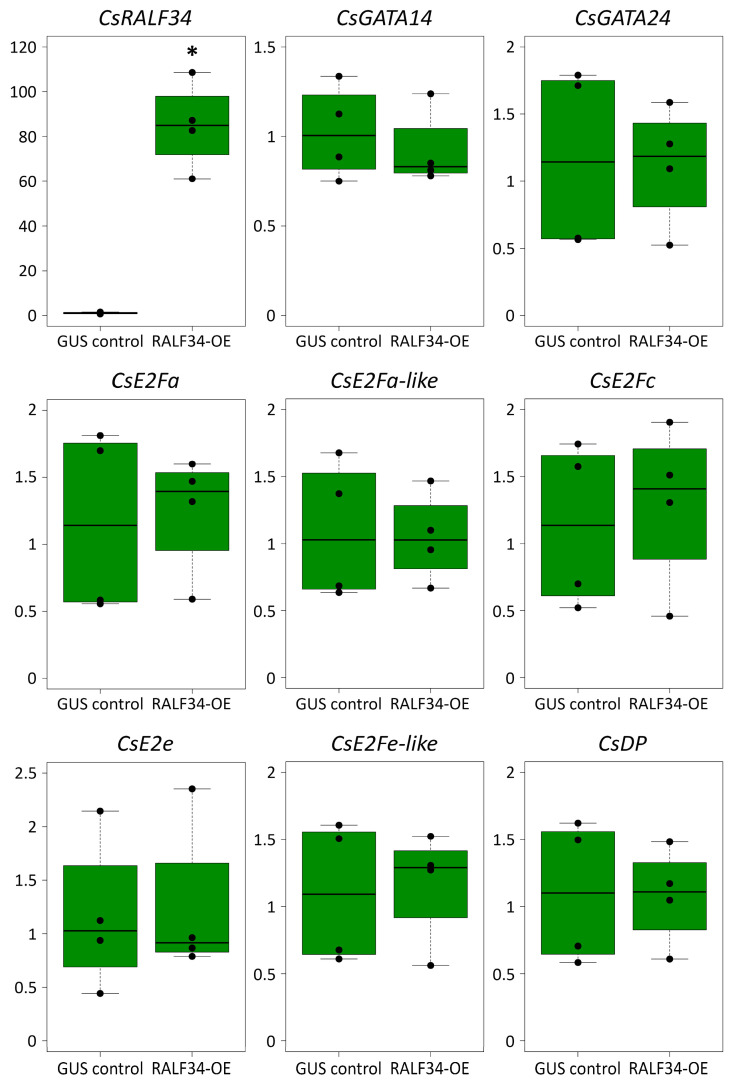
Relative transcript levels of *Cucumis sativus RALF34*, *GATA14*, *GATA24*, and *E2F/DP* genes in control roots and in roots overexpressing *CsRALF34*. GUS control, transgenic roots expressing *GUS*; RALF34-OE, transgenic roots overexpressing *CsRALF34*. Statistical analysis using Wilcoxon’s signed-rank test showed significant differences (*, *p* < 0.05) between expression levels in the overexpression group compared with the control only for *CsRALF34* (asterisk). The *y*-axis indicates relative transcript levels (2^−ΔΔCT^ method).

During the overexpression experiment (2.1), we also analyzed the expression levels of representatives of the *CsE2F*/*DP* family, i.e., *CsE2Fa*, etc., and of the cucumber orthologs of *AtGATA23*. The cucumber proteome contains six members of the E2F/DP protein family (*Cs*E2F/DP) (Figure 3). The expression of the six cucumber *CsE2F/DP* genes encoding these proteins were analyzed in control roots and in roots overexpressing *CsRALF34*. *CsRALF34* overexpression did not affect the expression levels of any of these genes (Figure 4). Expression of levels of the *GATA* genes, *CsGATA14* and *CsGATA24*, previously proposed to represent *AtGATA23* orthologs in cucumber [46], were examined as well. The expression levels of both genes did not change in response to *CsRALF34* overexpression (Figure 4).

### 2.4. Analysis of Biochemical Stress Response Markers in Cucumis Sativus Roots

**Figure 5:**
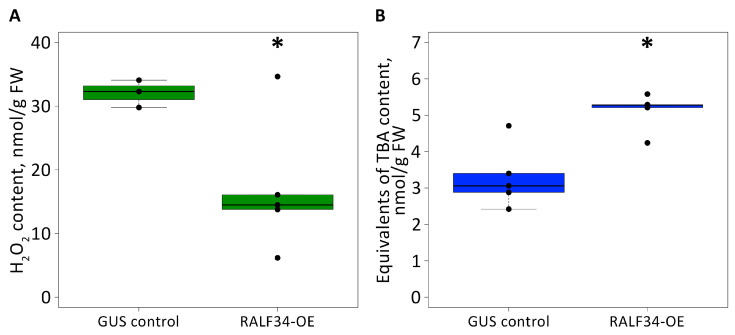
Biochemical characterization of *Cucumis sativus* transgenic control roots (*p35S::gusA*, GUS-control) compared with roots overexpressing *CsRALF34* (*p35S::CsRALF34*, RALF34-OE). (**A**) Hydrogen peroxide contents in control roots and roots overexpressing *CsRALF34*; (**B**) contents of thiobarbituric acid-reactive substance (TBARS) in control roots and roots overexpressing *CsRALF34* (determined as malondialdehyde equivalents). Asterisks denote statistically significant differences between groups of samples, *t*-test: *p* < 0.05. The raw data are summarized in Supplementary Information S2.

The effects of the RALF34 peptide might be related to the plant stress response. To obtain a preliminary overview of the dynamics of lipid peroxidation, membrane integrity, and the status of antioxidant defense in control roots vs. roots overexpressing *CsRALF34*, several markers of the cellular antioxidant defense status, i.e., the tissue levels of hydrogen peroxide, lipid hydroperoxides, thiobarbituric acid-reactive substances (expressed as malondialdehyde equivalents, TBARS), and ascorbic acid/dehydroascorbate balance, were analyzed (Figure 5). The results clearly indicated that hydrogen peroxide levels were significantly lower in roots overexpressing *CsRALF34* in comparison to control roots (Figure 5A). In contrast, the contents of thiobarbituric acid-reactive substances were increased almost two-fold in roots overexpressing *CsRALF34* compared with control roots (Figure 5B). On the other hand, the levels of lipid hydroperoxides and ascorbate were not affected by the overexpression of *CsRALF34* (Figure S1-2A,B). Interestingly, the contents of ascorbic acid were below the quantification limit in all samples, i.e., the ascorbate pool was represented only by the oxidized form of ascorbate, dehydroascorbate (data not shown).

### 2.5. Metabolomics Analysis

#### 2.5.1. Analysis of Primary Metabolites

The analysis of primary metabolites relied on two methods: gas chromatography-quadrupole mass spectrometry with EI ionization (GC-EI-Q-MS) and ion-pair reversed-phase high-performance liquid chromatography coupled online with triple quadrupole tandem mass spectrometry (RP-IP-HPLC-QqQ-MS/MS). After processing, both datasets were merged into one matrix prior to statistical evaluation.

The first approach, GC-EI-Q-MS, aimed to identify and relatively quantify thermally stable primary metabolites and revealed 321 total compounds, annotated in the aqueous methanolic extracts, obtained from cucumber control roots (GUS control) or from roots overexpressing *CsRALF34* (RALF34-OE), respectively (Table S1-4). In this group, 234 individual compounds were identified as primary metabolites by spectral similarity search against available libraries and/or co-elution with authentic standards. Some metabolites appeared as several isomers and/or methoxyamine-trimethylsilyl derivatives; therefore, the total number of metabolites identified was only 175. The identified metabolites represented 27 amino acids, 6 amines, 15 fatty acids and esters, 29 organic acids (lactic, glycolic, salicylic, benzoic acids, as well as di- and tricarboxylic intermediates of the TCA cycle), 53 sugars (monosaccharides with uronic, aldonic acids, and phosphorylated derivatives, di- and oligosaccharides), 16 phenolic compounds, 3 heterocyclic compounds, 3 co-factors, 1 sterol, and 22 representatives of other classes.

The second metabolomics approach, RP-IP-HPLC-QqQ-MS, aimed to identify and relatively quantify thermally labile metabolites and revealed 157 annotated analytes in total (Table S1-5). This included: 37 nucleosides and nucleotides and their derivatives; 26 amino acids; 8 sugars and their derivatives, sugar acids and sugar alcohols; 24 sugar phosphates; 27 carboxylic acids; 4 fatty acid derivatives; 7 non-nucleotide heterocyclic compounds, including 3 imidazoles, 3 inorganic compounds, 4 inositol phosphate derivatives, and 7 terpenes; 3 thioesters; 4 vitamins; 1 benzene derivative; and 2 representatives of other classes.

**Figure 6:**
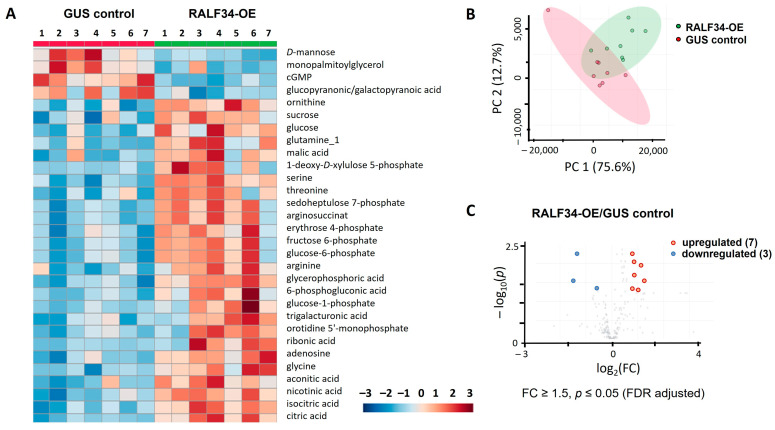
Illustration of quantitative differences in the profiles of primary metabolites associated with *CsRALF34* overexpression. (**A**) Results of hierarchical clustering with a heatmap representation of the 30 most abundant metabolites obtained from *Cucumis sativus* control roots and roots overexpressing *CsRALF34* (GUS control and RALF34-OE, respectively). (**B**) Principal component analysis with a score plot and (**C**) volcano plot with a graphical representation of differentially abundant analytes (Benjamini–Hochberg false discovery rate correction at *p* ≤ 0.05 and fold change (FC) ≥ 1.5) obtained for GUS control and RALF34-OE, respectively.

After merging the two result sets, a combined matrix with 391 entries was built and processed using the MetaboAnalyst 5.0 online software tool. Hierarchical clustering analysis with a heatmap representation of the normalized relative abundances corresponding to the individual metabolites revealed essential intra-group variability in both the control group and in *CsRALF34* overexpressing roots (Figure 6A). Thus, variability within the control group was higher than in the *CsRALF34*-overexpressing group (Figure S1-3). The principal component analysis did not show a complete separation of the two groups in the corresponding score plots, while 75.6% of the total variance could be explained by the first principal component (PC1, Figure 6B).

Despite the essential intra-group variability, the control group and the *CsRALF34* overexpressors demonstrated clear differences in their metabolic profiles. A *t*-test with Benjamini–Hochberg false discovery rate (FDR) correction at *p* ≤ 0.05 revealed that 26 metabolites were significantly downregulated in transgenic cucumber hairy roots overexpressing the *CsRALF34* gene (Figure S1-4). A fold change (FC) analysis (with an FC cut-off of FC ≥ 1.5) revealed 33 upregulated and 15 downregulated metabolites, demonstrating abundance dynamics associated with *CsRALF34* overexpression (Figure S1-5). Finally, the combination of these two criteria yielded ten metabolites, three of which were downregulated, while seven were upregulated upon the overexpression of *CsRALF34* (Table 1, Figure 6C). *D*-mannose, cGMP, and gluconic and/or galacturonic acid appeared to be most responsive to *CsRALF34* overexpression and demonstrated clear down-regulation in the RALF34-OE roots. The group of upregulated metabolites included nicotinic acid, adenosine, glucose-1-phosphate, arginine, orotidine 5′-monophosphate, ornithine, and 6-phosphogluconic acid.

#### 2.5.2. Analysis of Semi-Polar Secondary Metabolites

The analysis of semi-polar secondary metabolites revealed 486 and 374 compounds detected in negative and positive ion mode, respectively, which were organized in two data matrices according to their ionization mode. The principal component analysis (PCA) of both datasets combined revealed no group separation in the corresponding score plots (Figure S1-6A,B). As can be seen from the results of hierarchical clustering with heat map representation (Figure S1-6C,D), this observation might be explained by high intra-group variability, which was comparable to the inter-group dispersion. However, as can be seen from the corresponding volcano plots (Figure S1-6E,F), the majority of the analytes only exhibited minimal (within 1.5-fold) non-significant inter-group differences. Although several analytes demonstrated very strong FC responses to *CsRALF34* overexpression, the intensity of the corresponding levels varied strongly within the groups. Thus, in contrast to the primary metabolome, the secondary metabolome was not significantly affected by *CsRALF34* overexpression.

### 2.6. Protein Isolation and Tryptic Digestion

To ensure the efficient extraction of root proteins and the maximal possible coverage of the *Cucumis sativus* proteome, we selected the phenol-based protein extraction method. Determination of the protein concentrations in the obtained isolates revealed extraction yields in the range of 0.234–0.463 mg/g fresh weight (Table S1-3). The assay precision was determined by SDS-PAGE with 5 µg of protein per lane (Figure S1-7). Gel-based cross-validation by the densitometric assessment of Coomassie-stained gels revealed overall lane densities of 1.53 × 10^6^–1.82 × 10^6^ arbitrary units (AU, RSD = 5.8%, Table S1-6). This RSD value was sufficient for sample amount normalization and did not require additional recalculation. The signal patterns observed in the electropherograms were similar between lanes (Figure S1-7A,B). Tryptic digestion of the obtained protein isolates was considered to be complete, as no bands could be detected (Figure S1-7C,D) [47].

### 2.7. Annotation of Cucumis sativus Proteins

In total, 10,378 peptides were identified by MS/MS spectra in the whole dataset. Among them, 8561 and 8780 peptides were identified in control roots and in roots overexpressing *CsRALF34*, respectively (Figure S1-8A, Supplementary information S3, Table S3-1). Among these, 6983 peptides (67.3%) occurred in both groups, while 1598 (15.4%) and 1797 (17.3%) peptides were unique for either control or *CsRALF34-*overexpressing roots, respectively. Based on these identifications, 2302 possible proteins could be annotated (2147 and 2120 in control roots and in *CsRALF34* overexpressors, respectively, Figure S1-8B, Table S3-2), which represented 1946 non-redundant proteins (i.e., protein groups): 1821 vs. 1802 in control roots vs. *CsRALF34* overexpressors (Figure S1-8C, Table S3-3). Among these, 1965 proteins (85.4%) were common for both groups, 182 (7.9%) and 155 (6.7%) proteins were unique for controls or *CsRALF34* overexpressors, respectively (Figure S1-8B, Table S3-2). However, all these identifications relied exclusively on MS/MS spectra and did not consider inter-group alignments, which can result in the identification of some proteins as group-specific, although the corresponding peptide signal was detectable in the other group as well, but not fragmented. Fortunately, this issue can be solved by label-free quantification.

### 2.8. Label-Free Quantification

**Figure 7:**
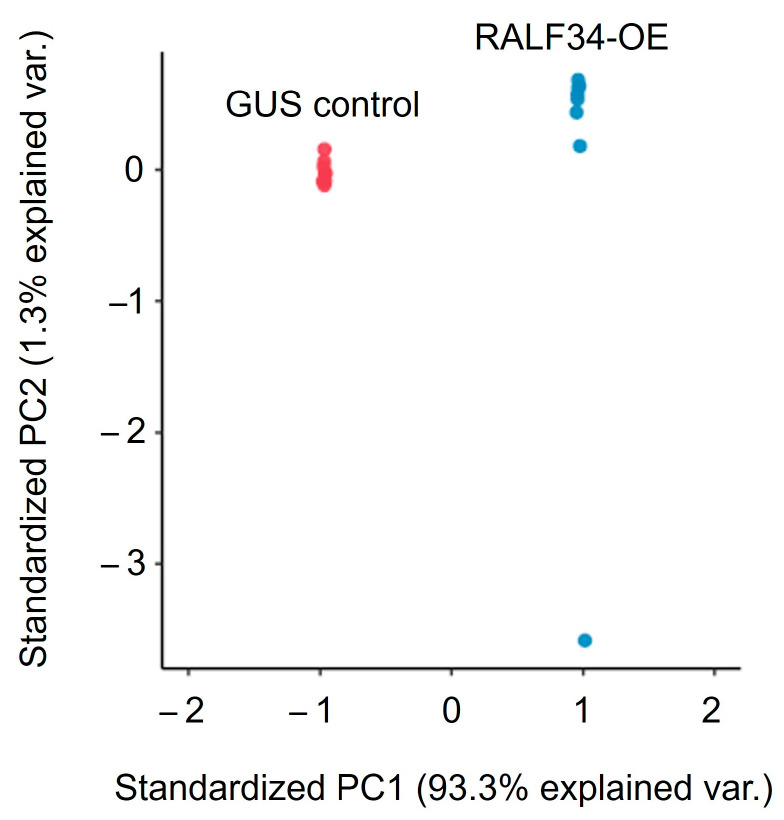
Principal component analysis with a score plot for transgenic *Cucumis sativus* roots containing either *p35S::gusA* (GUS controls) or *p35S::CsRALF34* (RALF34-OE). Principal component 1 (PC1) showed the inter-group differences (93% of explained variability) associated with *CsRALF34* overexpression.

In the first step, we characterized the observed differences by principal component analysis applied to the whole dataset (Figure 7). The results indicated low intra-group dispersion and a high degree of explained inter-group variance, i.e., more than 93% of group-specific proteins provided reliable group separation by principal component 1 (PC1). On the PC2 axis, no inter-group separation could be observed.

Label-free quantification revealed 208 differently expressed proteins (*t*-test: *p* ≤ 0.05, FC ≥ 1.5, Supplementary information S4) in cucumber hairy roots overexpressing *CsRALF34* among the 2302 annotated proteins. Among the differentially expressed proteins, 92 were upregulated, and 116 were downregulated in *CsRALF34* overexpressors compared with control roots (Table S4-1 and Table 2, respectively). The most strongly upregulated proteins were thioredoxin (17.8-fold), glutathione peroxidase (17.2-fold), carnitine operon protein CaiE (16.6-fold), and wound/stress protein (16.5-fold) (Table 2). The 70 kDa heat shock protein (21.1-fold), ubiquitin-like domain-containing CTD phosphatase (18.7-fold), and phloem lectin (18.2-fold) demonstrated the most pronounced downregulation (Table 2). PCA of the set of differentially expressed proteins confirmed the inter-group separation observed with the whole root proteome based on principal component 1 (PC1, 99.1% of the explained variance can be seen in the score plot presented in Figure S1-9A). To assess the contribution of individual proteins to the differences associated with *CsRALF34* overexpression, the corresponding loading plot, and biplot were analyzed (Figure S1-9B,C). The top 25 loadings which most strongly contributed to the *Cs*RALF34-associated alterations in the cucumber root proteome are listed in Table 2 (all loadings of differentially expressed proteins are listed in Table S1-7). Among them, thioredoxin, glutathione peroxidase, acidic endochitinase, tripeptidyl peptidase II, and protein transport protein SEC23 were upregulated (FC range 17.8–1.9-fold) and 70 kDa heat shock protein, ubiquitin-like domain-containing CTD phosphatase, phloem lectin, 10 kDa chaperonin, NADH dehydrogenase, and UDP-glycosyltransferase 1 were downregulated (FC range 21.1–1.6-fold).

### 2.9. Functional Annotation of CsRALF34-Regulated Proteins

**Figure 8:**
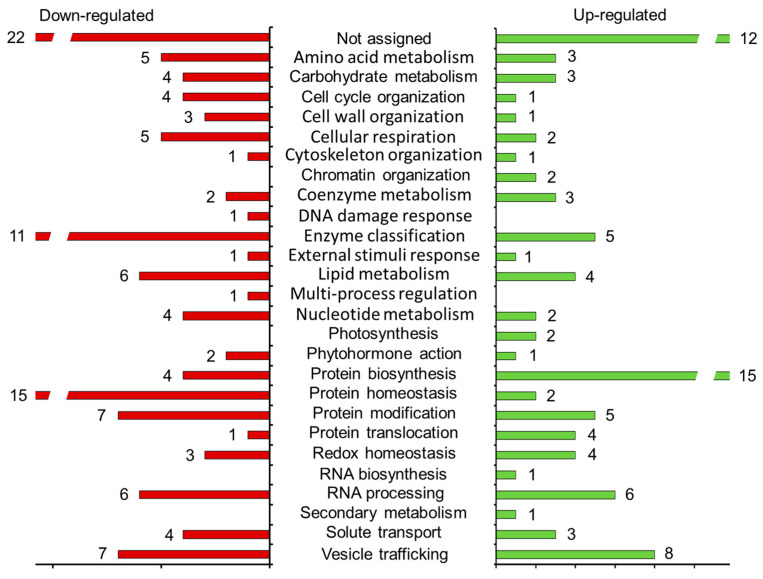
Functional annotation of the proteins identified as differentially abundant in *Cucumis sativus* transgenic roots overexpressing *CsRALF34* compared with transgenic GUS control roots. Numerical values indicate the numbers of proteins constituting individual up- (green) or downregulated (red) functional classes. The individual proteins comprising each functional group (in addition to all related information) are listed in Supplementary Information S5.

Functional annotation of the prospective *Cs*RALF34-dependently regulated proteome (i.e., 208 proteins identified as differentially expressed) relied on Mercator4 software. The analysis revealed 26 functional classes (bins, Figure 8, Supplementary Information S5) among the 29 available. The up- or downregulated proteins were represented by 25 and 22 bins, respectively (i.e., proteins involved in the DNA damage response, multi-process regulation, and solute transport were downregulated, whereas the polypeptides related to chromatin organization, photosynthesis, RNA biosynthesis, secondary metabolism, and solute transport were upregulated).

The group of upregulated proteins was dominated by those involved in protein biosynthesis (15 entries), with ribosomal proteins L19, L18a, L35, S24, and eukaryotic translation initiation factor 3 showing the highest abundance gain in *Cs*RALF34 overexpressors (17.0-, 16.4-, 15.7-, 16.7-, and 16.3-fold, respectively). Proteins involved in vesicle tracking also strongly contributed to the upregulated group with eight entries in total (for example, vacuolar-sorting receptor 7, exocyst complex component, and general vesicular transport factor p115 with 14.9-, 14.9-, or 13.1-fold changes in abundance) were part of this group. For 12 proteins (13.3% of all upregulated species), no function could be assigned in terms of the available bins; these were indicated as “not assigned” (Figure 8).

The group of downregulated polypeptides was dominated by those involved in protein homeostasis (15 entries) with 70 kDa heat shock protein, 10 kDa chaperonin, subtilisin-like serine protease, and Clp protease showing a decreased abundance in *Cs*RALF34 overexpressors (21.1-, 17.6-, 17.4-, and 16.2-fold, respectively). Enzymes involved in protein modification also strongly contributed to the downregulated group, represented by seven entries. These included glycylpeptide N-tetradecanoyltransferase and dolichyl-diphosphooligosaccharide-protein glycosyltransferase with 16- and 15.4-fold abundance changes, respectively. For 22 downregulated proteins (17.6%), the function could not be established, and they were indicated as “not assigned” (Figure 8).

**Figure 9:**
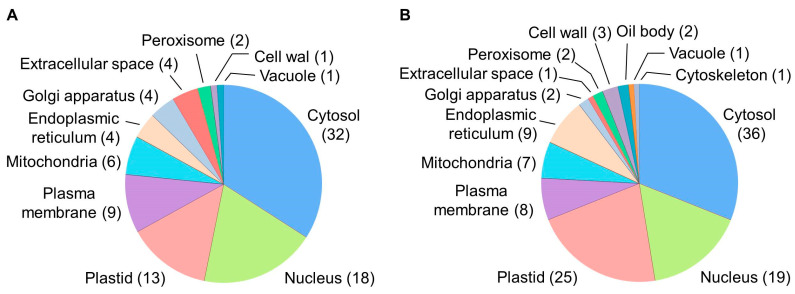
Prediction of the sub-cellular localization of proteins identified as up- (**A**) or downregulated (**B**) in *Cucumis sativus* transgenic roots overexpressing *CsRALF34*. Numerical values indicate the numbers of proteins with locations predicted to specific compartments. The individual proteins annotated to specific predicted compartments are listed in Supplementary Information S6.

The prediction of subcellular localization relied on WoLF PSORT with subsequent manual verification based on database and literature data (Supplementary Information S6). The results showed that the cytosol, plastids, and nucleus most strongly contributed to the *CsRALF34*-affected proteome (33.3%, 18.1%, and 17.6%, respectively (Figure 9A,B). On the other hand, the cell wall, peroxisome, oil body, and cytoskeleton proteins (1.9%, 1.9%, 1.0%, and 0.5%, respectively) were least affected by *CsRALF34* overexpression in cucumber roots. Interestingly, the localization patterns of up- and downregulated proteins were rather similar.

### 2.10. The Effects of CsRALF34 on Root Metabolic and Signaling Pathways

The metabolic and signaling pathways affected by *CsRALF34* overexpression were examined by mapping individual regulated proteins using the Kyoto Encyclopedia of Genes and Genomes (KEGG) database. Based on these data, the activation of specific signaling pathways, changes in sugar and tricarboxylic acid metabolism, and enhancement of protein biosynthesis and transport could be observed in root cells of *CsRALF34* overexpressors compared with control roots (Figure 8, Supplementary Information S5).

**Figure 10:**
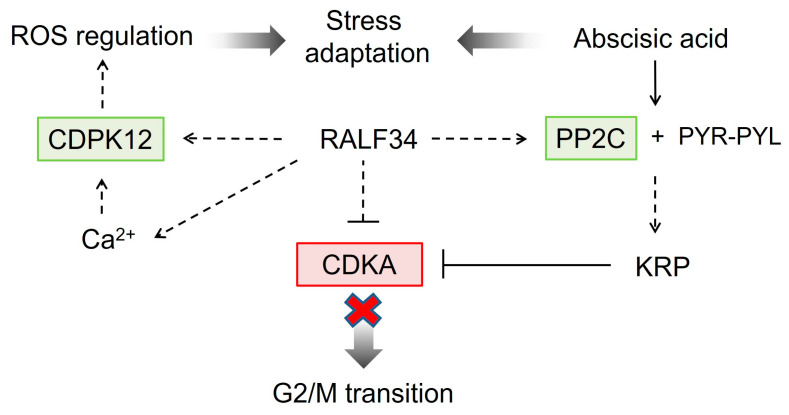
Signaling pathway involved in the regulation of the cell cycle and response to abiotic stress was activated in *CsRALF34* overexpressors. The up- or downregulated proteins are marked by green and red color, respectively. The pathway mapping relied on the KEGG signal pathway database, Shimotohno et al. [48], Dekomah et al. [49], and Asano et al. [50]. CDKA, cyclin-dependent kinase A (2.7.11.22); CDPK12, calcium-dependent protein kinase 12 (2.7.11.1); PP2C, protein phosphatase 2C (3.1.3.16); PYR-PYL, pyrabactin resistance, pyrabactin resistance-like component of abscisic acid receptor; ROS, reactive oxygen species; KRB, kip-related protein. Narrow arrows indicate individual steps of a pathway, dotted arrows indicate several steps of a pathway, while broad arrows point to the final effect of a pathway.

**Figure 11:**
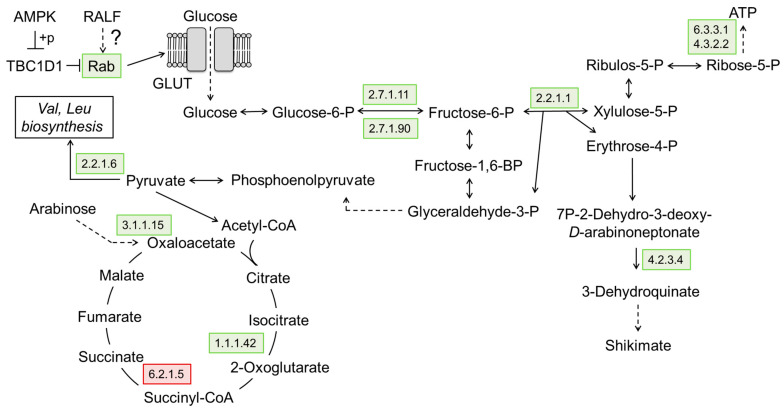
Metabolic pathways affected by the overexpression of *CsRALF34*. The up- or downregulated proteins are marked by green and red, respectively. Pathway mapping relied on the KEGG signal pathway database. AMPK, adenosine monophosphate-activated protein kinase; TBC1D1, TBC1 domain family member 1 (TBC1, tre-2/USP6, BUB2, CDC16); Rab, member of the Ras superfamily of small G proteins (Rab, Ras-related in brain); ATP, adenosine triphosphate; 5.4.2.8, mannose phosphomutase; 6.3.3.1, phosphoribosyl formylglycinamidine cyclo-ligase; 4.3.2.2, adenylosuccinate lyase; 2.7.1.11, 6-phosphofructokinase; 2.7.1.90, diphosphate-dependent phosphofructokinase; 2.2.1.1, transketolase; 2.2.1.6, acetolactate synthase; 4.2.3.4, 3-dehydroquinate synthase; 1.1.1.42, isocitrate dehydrogenase; 6.2.1.5, succinyl-CoA synthetase. Narrow arrows indicate one step of the pathway; dotted arrows indicate several steps.

In addition to the metabolic shifts, the overexpression of *CsRALF34* resulted in pronounced increases in the abundance of calcium-dependent protein kinase (2.7.11.1), protein phosphatase 2C (3.1.3.16), and checkpoint kinase (2.7.11.1). These proteins have been linked to cell cycle control and stress adaptation (Figure 10). Furthermore, the levels of thioredoxin 1, heat shock proteins Hsp 90 and Hsp 72, ubiquitin carboxyl-terminal hydrolase 1, universal stress protein, and glutathione peroxidase (1.11.1.9) were increased, whereas the relative abundances of NADH-dehydrogenase, proliferating cell nuclear antigen, glutathione S-transferase (2.5.1.18), and aminocyclopropane carboxylate oxidase (1.14.17.4) were decreased (Tables S4-1 and S4-2). Other effects of *CsRALF34* overexpression were increases in Rab-protein, enzymes of glycolysis, pentose phosphate pathway, mannose and purine metabolism, valine, leucine, and melatonin biosynthesis (Figure 11).

The relative abundances of TCA cycle enzymes were also changed in *CsRALF34* overexpressors. The levels of isocitrate dehydrogenase (1.1.1.42) were increased, whereas the relative contents of succinyl-CoA synthetase (6.2.1.5) were decreased (Figure 11). Furthermore, clear decreases in the relative abundances of aquaporin-7 (AQP-7) and oligosaccharyl transferase (OST) could be observed (Table S4-2).

Further cellular processes affected by *CsRALF34* overexpression were protein biosynthesis, transport, and exocytosis (Figure 8, Supplementary Information S5). RNA-binding family protein (Y14), ribosome biosynthesis proteins (NOP4, nucleolar protein 4 and NMD3, ribosome export adaptor), small and large ribosome subunit proteins (S10, 12, 24, 25, 29, 32, 38, L14, 15, 18, 19, and 35), mannosyl-oligosaccharide alpha-1,3-glucosidase (GlcII), protein transport protein Sec23/24, betaine-homocysteine S-methyltransferase (1.1.1.5), and 5-methyltetrahydropteroyltriglutamate-homocysteine methyltransferase (2.1.1.14) were upregulated in roots overexpressing *CsRALF34* (Table S4-1). Interestingly, several proteins involved in proteasomal degradation (PDIs, protein disulfide-isomerase A1, TRAP, translocon-associated protein subunit alpha, Hsp70, 70kDa heat shock protein, p97, transitional endoplasmic reticulum ATPase), protein transport into lytic vacuoles (VAMP7, vesicle-associated membrane protein 7) and endocytosis (dynamin, AP-2 complex subunit alpha (AP2), vacuolar protein-sorting-associated protein 4 (VPS4), and vacuolar protein sorting-associated protein (VTA1)) were downregulated in roots overexpressing *CsRALF34* compared with control roots (Table S4-2).

## 3. Discussion

### 3.1. CsRALF34 Is Not Involved in the Lateral Root Initiation

In this study, an integrated multiomics approach was used to elucidate the role of the small signal peptide *Cs*RALF34 in the development of cucumber root systems. The study was carried out on transgenic hairy roots of cucumber overexpressing *CsRALF34* compared with transgenic hairy roots expressing *p35S::gusA*. In the roots overexpressing *CsRALF34*, as confirmed by RT-qPCR, the lateral root initiation index (I_LRI_) remained unchanged compared with transgenic control roots, indicating that the number of initiated lateral root primordia was not affected (Figure 1).

In contrast, the number of lateral root primordia was reduced in Arabidopsis *ralf34* loss-of-function mutants [14]. Another effect on root development by *At*RALF34 was a change in the root growth rate. A stunted root growth rate was described for plants treated with the synthetic *At*RALF34 peptide compared with the control group [15]. Consistent with this result, *Atralf34* mutant roots were characterized by an increase in growth rate compared with the roots of wild-type plants [51]. Individual developing hairy roots always differ from each other in age and growth rate; therefore, the effects of *CsRALF34* overexpression on the growth rate of hairy roots cannot be determined. Therefore, wild-type cucumber seedlings’ roots were treated with synthetic *Cs*RALF34 to determine its effect on root growth. This effect was similar to that described for Arabidopsis [15]: treatment led to a statistically significant decrease in the root length increment (Figure 2). At the same time, the number of initiated lateral root primordia calculated using I_LRI_ was unchanged in cucumber roots treated with the synthetic *Cs*RALF34 peptide (Figure 2), as well as in transgenic roots overexpressing *CsRALF34* (Figure 1).

It has also been proposed that *At*RALF34 might indirectly regulate the expression of *AtGATA23* [14], the key marker gene of the early steps of lateral root initiation [22]. We previously proposed *CsGATA14* and *CsGATA24* to represent the putative cucumber orthologs of *AtGATA23* [46]. Here, we show that neither *CsGATA14* nor *CsGATA24* expression levels were changed in response to *CsRALF34* overexpression (Figure 4).

Next, we tested the hypothesis that the expression of *CsRALF34* may be involved in gene regulatory networks participating in the early steps of lateral root initiation. It is known that expression of the transcription factor *E2Fa* in Arabidopsis, a member of the *E2F*/*DP* family [52], is regulated by auxin-dependent activation of the transcription factors LBD18/LBD33 [53]. This is a common mechanism of the auxin-dependent activation of the mitotic cycle occurring in the xylem pole of the pericycle before the first asymmetric division during lateral root initiation. Phylogenetic analysis of Arabidopsis and cucumber E2F/DP (*Cs*E2F/DP) proteins was carried out, leading to the identification of cucumber orthologs of Arabidopsis *E2F*/*DP* genes (Figure 3). Expression levels of all *CsE2F*/*DP* genes were not changed in response to *CsRALF34* overexpression (Figure 4).

Taken together, the data obtained using I_LRI_ calculations for transgenic hairy roots overexpressing *CsRALF34*, as well as for *Cs*RALF34-treated roots, and the evaluation of *CsE2F*/*DP*, *CsGATA14*, and *CsGATA24* expression suggest that *Cs*RALF34 does not represent a key player in lateral root initiation occurring in the root apical meristem of the cucumber parental root. However, although *CsRALF34* overexpression does not affect lateral root initiation (i.e., it does not change the number of initiated lateral roots), an effect on the root growth rate cannot be excluded at this point.

The absence of any visible phenotype in roots overexpressing *CsRALF34* prompted us to perform a comprehensive analysis of changes in the proteome and metabolome in response to *CsRALF34* overexpression. Protein isolation followed by tryptic digestion was performed. The variability in protein yields (0.234–0.463 mg/g fresh weight, Table S1-6) was relatively low, in agreement with the low amounts of protein in roots compared with other parts of the plant [54,55]. Importantly, the precise estimation of protein extraction recoveries (RSD = 5.8%) were in line with our prior results [56]. Therefore, the observed lower variability in protein yields and precise estimation of the protein concentrations in the extracts enabled the reliable implementation of label-free quantification in the analysis of heterogeneous root proteomes [47].

PCA applied to the whole proteome (Figure 7) exhibited low intra-group variance and a high degree of inter-group variance (more than 93%). These results indicated a high likelihood that the observed differences were predominantly associated with the effects of *CsRALF34* overexpression.

### 3.2. The Functions of CsRALF34

#### 3.2.1. Activation of Protein Biosynthesis

**Figure 12:**
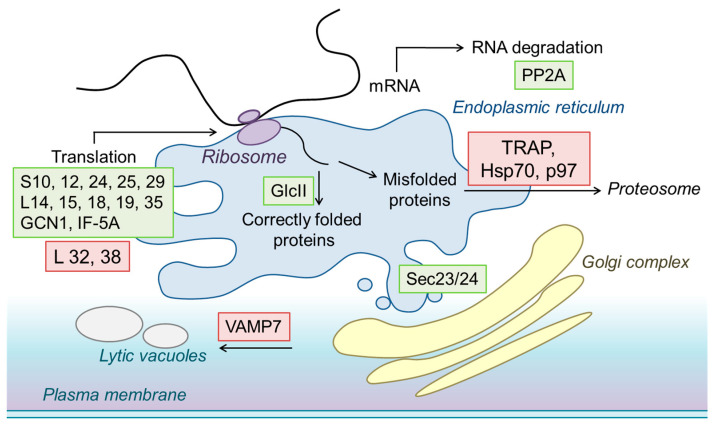
The pathways of protein synthesis and transport activated by *CsRALF34* overexpression (based on the KEGG signal pathway database). Green, upregulated; red, downregulated proteins. PP2A, serine/threonine-protein phosphatase 2A; S10, 12, 24, 25, 29, 32, and 38, proteins of the small ribosomal subunit; L14, 15, 18, 19, and 35, proteins of the large ribosomal subunit; GlcII, mannosyl-oligosaccharide alpha-1,3-glucosidase; TRAP, translocon-associated protein subunit alpha; Hsp70, heat shock 70 kDa protein; p97, transitional endoplasmic reticulum ATPase; VAMP7, vesicle-associated membrane protein 7; Sec23/24, protein transporter from ER to Golgi.

Our experiments clearly demonstrated that the overexpression of *CsRALF34* resulted in a pronounced upregulation of proteins involved in protein biosynthesis and transport (Figure 12). Upregulation of proteins constituting the small and large ribosomal subunits, RNA-binding family protein, nucleolar protein 4, ribosome export adaptor, protein transporter Sec23/24, and others were observed (Figure 12, Table S4-1).

#### 3.2.2. Inhibition of Root Growth and Regulation of Cell Proliferation

To date, in several studied plant species (e.g., *Medicago truncatula* and *Solanum lycopersicum*), the main role of the RALF34 peptide was reported to be the control of the alkalization of the environment for growth inhibition by suppressing the functions of H^+^-ATPase [11,16,57]. This should also prevent cell elongation. However, in other studies, this effect was discussed as a result of the molecular signaling cascade activated by RALF34. Pearce, Moura, Stratmann, and Ryan [11] showed that the expression of *RALF34* was associated with a decrease in the activity of the mitogen-activated protein kinase (MAPK), which was associated with the inhibition of lateral root growth. The data presented here revealed that the overexpression of *CsRALF34* in cucumber roots resulted in a decrease in CDKA levels, which, in turn, could lead to a G2/mitosis transition block [48].

Furthermore, we observed downregulation of the proliferating cell nuclear antigen (PCNA; Csa6g188070.1, Table S4-1), which is known to be involved in DNA replication and serves as an interaction partner of DNA polymerases [58,59]. Combined with the decrease in the levels of helicases, these data support the assumption that *Cs*RALF34 overexpression results in blockage of G2/mitosis transition in cucumber root cells (Figure 10).

#### 3.2.3. Impact of CsRALF34 on ROS Signaling and Stress Adaptation

The exact mechanisms behind the contribution of RALF peptides to stress response and adaptation are still unknown. However, Stegmann et al. [60] showed that several *At*RALFs are involved in the regulation of intracellular ROS levels. The authors reported that *At*RALF23 mediated an increase in ROS production, whereas *At*RALF17, in contrast, caused a decrease in ROS levels. Our results generally support the role of RALF peptides as ROS regulators, suggesting that *Cs*RALF34 modulates ROS homeostasis. At least *Cs*RALF34 caused alterations in the function of the respiratory chain combined with activation of the antioxidant defense systems.

We observed decreases in the relative abundances of NADH-dehydrogenase, complex I of the respiratory chain, and succinyl-CoA synthetase, the enzyme that converts succinyl-CoA to succinate (Supplementary Information S4, Table S4-2). Succinate deficiency is known to suppress the activity of succinate dehydrogenase, which is an enzyme of the TCA cycle and simultaneously represents complex II of the mitochondrial respiratory chain. The loss of its activity leads to a deficit of electrons in the respiratory chain. Moreover, as NADH-dehydrogenase reduces ubiquinone, which, in its reduced form, is the substrate of cytochrome c reductase, the activity of complex III could also be negatively affected by *Cs*RALF34. Thus, given that complexes I and III are the main generators of the superoxide anion radical [61,62], this decrease in the electron flow through the entire respiratory chain might lead to a decrease in the rate of superoxide generation.

As shown here, the levels of H_2_O_2_ were significantly lower in roots overexpressing *CsRALF34* compared with control roots (Figure 5A). This can be explained by the inhibition of electron transfer along the respiratory chain described above because superoxide anions are converted to H_2_O_2_ by superoxide dismutase. On the other hand, the increased levels of thioredoxin 1, heat shock proteins Hsp90 and Hsp72, ubiquitin carboxyl-terminal hydrolase 1, and universal stress protein indicate that the cellular antioxidant defense was upregulated.

Equally importantly, glutathione peroxidase levels were also upregulated in response to *Cs*RALF34 overexpression (Table S4-1). This enzyme is directly involved in antioxidant defense, specifically in the reduction of lipid hydroperoxides to the corresponding alcohols and in the reduction of hydrogen peroxide to water [63]. Thus, it can be concluded that *Cs*RALF34 is critically involved in the suppression of H_2_O_2_ production and the activation of multiple cellular antioxidant systems. It can be assumed that *Cs*RALF34 mediated these effects via signaling cascades.

Due to their upregulation upon *CsRALF34* overexpression, we could identify protein phosphatase 2C (PP2C) and calcium-dependent protein kinase 12 (CDPK12) as the components of the signaling pathways involved in the RALF34-dependent responses. PP2C is involved in abscisic acid (ABA) signal transduction. Binding of ABA to its receptors, the pyrabactin resistance/pyrabactin resistance-like/regulatory components of the abscisic acid receptor (PYR/PYL/RCAR), leads the receptors to bind to PP2C, which, in turn, leads to its inhibition and the activation of the SNF1-related protein kinase 2 (SnRK2). In the absence of ABA, SnRK2 proteins are inhibited by PP2C-dependent dephosphorylation (Hsu et al., 2021). Briefly, PP2C plays an important role in ABA signaling [64]. Importantly, CDPK12 is known to contribute to regulating the expression of antioxidant defense genes; therefore, its activity might affect plant adaptations to stress [50]. Thus, the RALF34-induced upregulation of PP2C could affect ABA signaling and, thereby, plant adaptation to osmotic stress. In this context, it is possible that the upregulation of the cellular antioxidant pathways, the decrease in ROS production, and other metabolic shifts (Figure 10 and Figure 11) could be mediated by increases in *PP2C* expression levels.

The involvement of ROS in this process was supported by the analysis of the tissue oxidative status in control and *CsRALF34* overexpressing roots (Figure 5 and Figure S1-2). Consistent with the upregulation of the ROS-detoxifying enzymes, a clear decrease in H_2_O_2_ content was observed in *CsRALF34* overexpressing roots. On the other hand, levels of thiobarbituric acid-reactive substances were increased in *CsRALF34* overexpressors compared with wild-type roots, indicating a complex effect of RALF34 on different aspects of antioxidant defense.

This conclusion is in agreement with the work of Song et al. [65], who showed that a mutation in FERONIA (FER), the RALF receptor gene in Arabidopsis, was associated with lower levels of ROS formation. Thus, it appears to be likely that RALF might directly affect ROS production. Moreover, because CDPK is activated by interaction with Ca^2+^ ions [66], upregulation of the production of this kinase might result in increased sensitivity of the root to intracellular calcium levels. Upon treatment with nanomolar concentrations of natural or synthetic RALF peptides, the increasing levels of cytoplasmic Ca^2+^ reached their maximum within 40 s [67]. This permits us to speculate that different RALF peptides can act in a concerted way; some RALF peptides might bind to the RALF receptor and initiate the release of Ca^2+^, whereas the others could increase the levels of sensitivity to ABA. Finally, it is important to mention that Ca^2+^ levels might fluctuate strongly even over short periods of time. Interference of these dynamically altering calcium levels with RALF-mediated responses might underlie a plethora of RALF effects.

#### 3.2.4. RALF-Related Dynamics of Cellular Metabolism

Based on the results of our study, *Cs*RALF34 appeared to affect the energy metabolism of the root cell. As mentioned above, the overexpression of *CsRALF34* was accompanied by the downregulation of NADH-dehydrogenase and succinyl-CoA synthetase. It appears likely that a compensatory mechanism of ATP generation was in effect, related to the upregulation of the TCA cycle, glycolysis, pentose phosphate pathway, and purine biosynthesis de novo (Figure 11); the effect of *Cs*RALF34 treatment on the root growth increment was not dramatic.

Altogether, the analysis of the primary and secondary metabolome showed that the primary metabolome was much more affected by *CsRALF34* overexpression (Table 1). The levels of *D*-mannose and cGMP were decreased, whereas the levels of glucose-1-phosphate, 6-phosphogluconic acid, ornithine, and adenosine were upregulated (Table 1). These results are consistent with the proteome analysis data with regard to enzymes involved in sugar metabolism.

#### 3.2.5. RALF34 as a Modulator of Phytohormone Responses

Currently, the effect of RALF peptides on hormonal regulation is discussed in the literature [12]. In the most comprehensive way, this aspect was addressed in the studies of *fer* mutants, i.e., the plants defect at the gene of receptor-like kinase FER, which is one of the RALF receptors [28]. As was shown recently, this receptor is involved in the modulation of jasmonic acid, ethylene, ABA, and brassinosteroid dynamics in plants [28].

The results of this study (Section 2.10, Figure 10) clearly indicate an upregulation of PPC2, which can be expected to lead to a downregulation of SnRK2. This, in turn, would affect responses to ABA, ethylene, jasmonic acid, and to abiotic stress in general (Figure 10). This is consistent with the observation that a *fer* loss-of-function mutant demonstrated hypersensitivity to both ABA and abiotic stresses associated with exposure to high salt concentrations and low temperatures [68]. The results of this study support the assumption that RALF34 is involved in the response to abiotic stress.

In addition to the effects related to the ABA signaling pathway, the overexpression of *CsRALF34* was associated with a downregulation of aminocyclopropane carboxylate oxidase, which is responsible for the final step of ethylene biosynthesis [69]. This fact might suggest a decrease in the ethylene levels in the roots overexpressing *CsRALF34*. Notably, Mao et al. [70] showed that Arabidopsis *fer* mutants displayed higher levels of ethylene in plant tissues combined with a dwarf phenotype. Therefore, the authors assumed that the FER receptor kinase interacts with S-adenosylmethionine synthases and downregulates ethylene biosynthesis in response to environmental stress, as well as the external application of auxin and brassinosteroids [70]. Remarkably, Deslauriers and Larsen [71] showed that mutation of *fer* led to a loss of sensitivity to brassinosteroids and resulted in enhancement of the ethylene response. Finally, Bergonci et al. [13] showed that RALF1 can compete with brassinosteroids for components shared by both signal transduction pathways.

In summary, the available data are consistent with our results. It is likely that after binding to its receptor, RALF34 suppresses the expression of aminocyclopropane carboxylate synthase, which then leads to a decrease in ethylene levels.

## 4. Materials and Methods

### 4.1. Plant Material and Bacterial Strains

Cucumber (*Cucumis sativus* L.) cv. Phoenix (Sortsemovosch, Saint Petersburg, Russia) was used in this study.

*Escherichia coli* strain XL-1 Blue was used for molecular cloning. *Rhizobium rhizogenes* (*Agrobacterium rhizogenes*) strain R1000 was used for the genetic transformation of plants.

### 4.2. Reagents

The full list of the reagents is given in Supplementary Information S1 (Protocol S1-1).

### 4.3. Phylogeny and Bioinformatics

Sequences of six Arabidopsis E2F proteins [72] and two Arabidopsis DP proteins [73] were downloaded from the Arabidopsis Information Resource (TAIR, www.arabidopsis.org, accessed on 10 March 2023) [74] and used as a query to find amino acid sequences of *C. sativus* (cucumber, cv. Chinese Long v2) [75] in the Cucurbit Genomics Database v1 (CuGenDB v1, cucurbitgenomics.org, accessed on 10 March 2023) [76]. All alignments were performed using online Clustal Omega software (www.ebi.ac.uk/Tools/msa/clustalo/, accessed on 10 March 2023) [77] at default settings. The alignment file was transferred into MEGA7.0 software [78], followed by phylogenetic tree construction using the Maximum Likelihood method [79]. The Jones–Taylor–Thornton model [80] with evolutionary rate differences among sites (+G parameter) (Yang, 1994) was used for phylogeny reconstruction of the E2F/DP family in cucumber. Phylogeny was tested using the bootstrap method with 1000 replicates.

### 4.4. RT-qPCR Assays

Total RNA was extracted from frozen plant material using ExtractRNA reagent (Evrogen, Moscow, Russia). RNA quantity and integrity were measured using a Qubit 4.0 fluorimeter (Thermo Fisher Scientific, Waltham, MA, USA) using Qubit RNA BR Assay and IQ Assay Kits. Reverse transcription was performed as described previously [46]. For each qPCR assay, 1 μL of cDNA (80–120 ng) from a non-diluted sample (total volume 20 μL) was used.

The RT-qPCR analysis was performed using a Quant Studio 5 Real-Time PCR system (Thermo Fisher Scientific) in a total volume of 20 μL. qPCR and PCR conditions were described previously [46]. Primers used for qPCR (Table S1-1) were designed using Vector NTI Advance v 11.0 software (Thermo Fisher Scientific). Purified PCR primers were purchased from Evrogen (Moscow, Russia). Quantification cycles (Cq) were determined using Quant Studio Design and Analysis software v. 1.5.1 (Thermo Fisher Scientific). Relative transcript levels were calculated as described previously [46]. Elongation factor *EF1α* was chosen as a reference gene according to data on the stability of reference gene expression in cucumbers presented in the literature [81].

RT-qPCR analysis of *CsRALF34* relative expression levels in the first replicate of the overexpression experiment was performed five times independently from the control group (GUS control, mixed root sample) and for each individual transgenic root (RALF34-OE, *n* = 9). The second replicate of the *CsRALF34* overexpression experiment was conducted to analyze the transcript levels of Cs*RALF34* as well as the *CsGATA14*, *CsGATA24*, and *CsE2F/DP* genes in response to *CsRALF34* overexpression. RT-qPCR analysis of the second replicate was performed four times independently.

### 4.5. Lateral Root Initiation Index (I_LRI_) Estimation

The Lateral Root Initiation Index (I_LRI_) [82] was estimated for two groups of roots: individual transgenic roots overexpressing *CsRALF34* (*n* = 8) and GUS control roots (*n* = 8), as well as for wild-type roots treated with synthetic *Cs*RALF34 peptide (*n* = 10) and for corresponding wild-type control roots (*n* = 14). The I_LRI_ was calculated on longitudinal root sections as described previously [83]. The most proximal lateral root primordia were detected in the region located 2–5 mm from the root tip above the division zone of the cortex [84].

### 4.6. Treatments with Synthetic CsRALF34 Peptide

A synthetic *Cs*RALF34 peptide (FWRRVHYYISYGALSANRIPCPPRSGRPYYTHNCYKARGPVNPYTRGCSAITRCRR; >87% purity) was synthesized by ProteoGenix (Schiltigheim, France), and its structure was validated independently by mass spectrometry. Lyophilized peptide was diluted in a mixture of acetonitrile and double distilled water (1:3). Five-day-old wild-type cucumber seedlings with 5–7 cm long roots were incubated in aerated Hoagland’s medium [85] supplemented with 2 μM *Cs*RALF34 [15,86,87] or with the same volume of the solvent (control) for 48 h. Each experiment included 50 seedlings (both in the control and in the group with *Cs*RALF34 treatment) and was repeated twice independently. For each seedling, the root length was measured before and after peptide treatment.

### 4.7. Molecular Cloning and Vector Design

Two genetic constructs were developed for overexpression assays using multisite Gateway technology (Gateway LR Clonase II plus, Thermo Fisher Scientific): for *CsRALF34* overexpression using the *35S*CaMV promoter and a control vector for *gusA* (*β-glucuronidase A*) overexpression under the control of the same promoter. The pKGW-RR-MGW binary vector, carrying the *pAtUBQ10::DsRED1* [88] screening cassette in the backbone, was used as the destination vector (kindly provided by Erik Limpens, Wageningen University, Wageningen, The Netherlands). LR plus clonase reactions were prepared according to the manufacturer’s instructions. The sequences of the cassettes of all constructs were verified by the PCR amplification of fragments and sequencing of the products. All primer sequences and their combinations are listed in Tables S1-2 and S1-3.

The *p35S*-pENTRattL4attR1 entry vector (Wageningen University, The Netherlands) was used as a donor of the *35S*CaMV promoter. Two entry vectors were developed harboring *CsRALF34* or *gusA* (*β-glucuronidase A*) coding sequences, respectively. *CsRALF34* was PCR amplified using cucumber genomic DNA as a template, cloned into the pJET1.2 vector (Thermo Fisher Scientific), and verified by sequencing. Then, *CsRALF34* and *gusA* coding sequences were amplified using *CsRALF34*-pJET1.2 and *NLS-Egfp-gusA*–pUC18-entry8 as templates [83], respectively, and *Kpn*I-*Not*I cloned into the pUC18-entry8 vector [89]. pENTRattR2attL3-*TermAct* [46] was used as a donor of the *Arabidopsis thaliana Actin2* gene terminator. The resulting constructs were pKGW-RR-MGW-***p35S::CsRALF34*** for *CsRALF34* overexpression assays and pKGW-RR-MGW-***p35S::gusA*** as controls (Figure S1-1). The binary vectors were introduced in agrobacterial cells by electroporation [90].

### 4.8. Plant Transformation and Handling for CsRALF34 Overexpression Assays

*Agrobacterium rhizogenes* (*Rhizobium rhizogenes*)-mediated transformation of cucumber seedlings was carried out as described previously [46,90] with minor modifications. The experiment was performed in two replicates. At least 25 composite plants with roots overexpressing *CsRALF34* (*p35S::CsRALF34*) and GUS control plants (*p35S::gusA*) were obtained in each transformation. Transformants were cultured in vermiculite moistened with 4x Hoagland’s medium. Transgenic roots 6–10 cm in length were harvested from both groups four times at 7-day intervals and flash-frozen in liquid nitrogen. The total number of roots was as follows: 220 for GUS control plants and 246 for *CsRALF34* overexpressors.

### 4.9. Determination of Hydrogen Peroxide Contents

Analysis of H_2_O_2_ contents in cucumber roots relied on the method described by Chantseva et al. [91], with modifications explained in Supplementary Information S1 (Protocol S1-2). The absorbance was measured at 575 nm (length of the optical path: 1 cm), and calculations were performed as described previously [91].

### 4.10. Determination of Lipid Peroxidation Product Contents

Lipid peroxidation products were quantified as malondialdehyde equivalents according to the protocol described by Soboleva et al. [92], with modifications presented in Supplementary Information S1 (Protocol S1-3).

### 4.11. Determination of Lipid Hydroperoxide Contents

Analysis of lipid hydroperoxide contents relied on the method of Frolov et al. [56], with modifications described in Supplementary Information S1 (Protocol S1-4).

### 4.12. Determination of Ascorbic Acid Contents

The determination of ascorbate contents and the respective statistical calculations were performed as described by Shumilina et al. [54] with modifications presented in Supplementary Information S1 (Protocol S1-5).

### 4.13. Analysis of Primary Metabolites

The primary metabolomics study relied on two techniques: thermally stable metabolites were analyzed by gas chromatography-electron ionization-quadrupole-mass spectrometry (GC-EI-Q-MS), whereas the temperature-labile metabolites were analyzed by reversed-phase-ion-pair-ultrahigh-performance liquid chromatography-electrospray ionization-triple quadrupole tandem mass spectrometry (RP-IP-UHPLC-ESI-QqQ-MS/MS) (Table S1-9, Protocol S1-6). The analysis of temperature-labile anionic primary metabolites was described by Leonova et al. [93].

### 4.14. Analysis of Semi-Polar Secondary Metabolites

The semi-polar secondary metabolites were analyzed using chromatography and mass spectrometry (RP-HPLC-ESI-QqTOF-MS) settings specified in Supplementary Information S1 (Table S1-10, Protocol S1-7).

### 4.15. Processing of the Acquired Metabolomics Data

Data pre-processing and processing relied on the Automated Mass Spectral Deconvolution and Identification System v.2.66 (www.amdis.net, accessed on 10 March 2023), Xcalibur v.2.0.7 and LCquan v.2.5.6 (Thermo Fisher Scientific), MSDial v.3.12 (prime.psc.riken.jp/Metabolomics_Software/MS-DIAL/, accessed on 10 March 2023). For interpretation of the LC-MS data LabSolution (Shimadzu, Kyoto, Japan) and MSDial (prime.psc.riken.jp/compms/msdial/, accessed on 10 March 2023) were used. Metabolite identification relied on a broad panel of available spectral libraries: National Institute of Standards and Technology (NIST), Golm Metabolome Database (GMD), Human Metabolome Database (HMDB), MS/MS and electron ionization (EI)-MS spectra curated by RIKEN Center for Sustainable Resource Science (prime.psc.riken.jp/Metabolomics Software/MS-DIAL/, accessed on 10 March 2023), and an in-house library (partially with Kovats retention time indices, calculated by the retention times of alkane standards). The post-processing and statistical interpretation of the acquired data relied on MetaboAnalyst 5.0 (www.metaboanalyst.ca, accessed on 10 March 2023) [94].

### 4.16. Protein Isolation and Determination

For protein extraction, approximately 250 mg of milled frozen root material was used, as described previously [95], with some modifications (Supplementary Information S1 (Protocol S1-6)). The precision of the assay was cross-verified by SDS-PAGE, according to Greifenhagen et al. [96], with some modifications. Briefly, after the gels were stained with 0.1% Coomassie Brilliant Blue G-250 for 12 h, the average densities across individual lanes (expressed in arbitrary units) were determined using a ChemiDoc XRS imaging system controlled by Quantity One 1-D analysis software (Bio-Rad Laboratories, Hercules, CA, USA). To calculate the relative standard deviations (RSDs), the densities of individual lines were normalized to the gel average value by ImageJ software [97].

### 4.17. Tryptic Digestion and Sample Pre-Cleaning

The day before the digestion procedure, Amicon Ultra 30K filter units (Sigma-Aldrich, Saint-Louis, MO, USA) were passivated in 500 µL 5% Tween-20 by shaking (350 rpm, 25 °C, 12 h). The next day, the filter units were washed twice with Milli-Q water while shaking (450 rpm, 30 min, 25 °C). Subsequently, 35 µg of protein was transferred to filter units, and 200 µL of urea solution (8 M urea, 50 mM Tris-HCl buffer, pH 7.5, UA) was added. Then the tubes with filters were centrifuged (14,000× *g*, 10 min, 4 °C), and the supernatants were discarded. All these steps with UA were repeated three times. Furthermore, 100 µL of reducing solution (100 mmol/L dithiothreitol in UA) was added, and incubated under continuous shaking (450 rpm, 25 °C, 1 h). Then, the mixtures were centrifuged (14,000× *g*, 10 min, 25 °C), and the supernatants were discarded. The same centrifugation and discarding steps were performed after the subsequent addition of 100 µL of alkylation solution (50 mmol/L iodoacetamide in UA); the incubation under continuous shaking (450 rpm, 25 °C, 1 h) was performed in the dark. The next step was washing filters with 200 µL of UA twice (centrifugation at 14,000× *g*, 10 min, 4 °C and discarding of supernatant after each step). Then, the same procedures were carried out with the digestion buffer (50 mmol/L ammonium bicarbonate, ABC) twice. The stock solution of trypsin (0.5 µg/µL) was freshly prepared, added to the solutions on the filters, and then incubated under continuous shaking (450 rpm, 37 °C, 4 h). The incubations were further continued after the addition of trypsin stock solution (enzyme-to protein-ratio *w*/*w* 1:50) under continuous shaking (450 rpm, 37 °C) overnight. The samples were centrifuged (14,000× *g*, 10 min, 25 °C), and the filtrates containing the mixtures of proteolytic peptides were supplemented with 40 µL of ABC buffer and centrifuged again (14,000× *g*, 10 min, 25 °C). This step was repeated twice; then, proteolytic hydrolysates were frozen at –20 °C. The completeness of the digestion was verified by SDS-PAGE [96].

### 4.18. Solid Phase Extraction

The proteolytic hydrolysates were pre-cleaned by reverse-phase solid-phase extraction (RP-SPE) using the elution scheme of Spiller et al. [98], with minor modifications specified in Supplementary Information S1 (Protocol S1-7).

### 4.19. Nano LC-MS/MS

The protein hydrolysates were loaded on an Acclaim PepMap 5 mm Trap Cartridge (Thermo Fisher Scientific) and separated on a Bruker FORTY separation column (C18 ReproSil AQ, 40 cm × 75 µm, 1.9 µm, 120 A; Bruker Daltonics, Bremen, Germany) using a nanoElute UHPLC chromatography system (Bruker Daltonics) coupled on-line to a TimsToF Pro quadrupole time-of-flight mass spectrometer (QqTOF-MS) via a CaptiveSpray ion source (Bruker Daltonics). The details of the chromatographic separation method are summarized in Supplementary Information S1 (Table S1-11). The UHPLC-QqTOF-MS/MS analysis relied on data-dependent acquisition experiments performed in the positive ion mode, comprising a survey TOF-MS scans and dependent MS/MS scans for the most abundant signals in the following 3 s (at certain t_R_) with charge states ranging from 2 to 5. The mass spectrometer settings are summarized in Table S1-12.

### 4.20. Data Post-Processing and Analysis

The acquired raw LC-MS data were processed with PEAKS Studio software (v. 10.6, Bruker Daltonics) (for the search settings, see Table S1-13). Thereby, the identification of peptides and annotation of proteins relied on a search against amino acid sequences of *Cucumis sativus* cv. Chinese Long v2 [75] (Cucurbit Genomics Database v1) accomplished with the SEQUEST algorithm [99,100]. Proteomics data were post-processed in RStudio (posit.co, accessed on 10 March 2023), which is an R programming language environment [101]. Quantitative protein analysis was accomplished on *log2*-transformed normalized data using the *limma* package with the minimal abundance alteration cut-off of 1.5 logarithmic fold change [102]. Additionally, Mercator4 (v. 2.0, plabipd.de/portal/mercator4, accessed on 10 March 2023) was used for protein annotations [103,104]. The subcellular localization of proteins was defined using WoLF PSORT [105]. Enrichment analysis was carried out using KOBAS-i [106].

### 4.21. Statistical Analysis

All boxplots were prepared using R programming language [101] and RStudio software (posit.co, accessed on 10 March 2023). The default code for the boxplot and stripchart functions from the base R package were used. Statistical analyses of qPCR, *Cs*RALF34 treatment, and I_LRI_ data were performed using Wilcoxon’s signed-rank test from the base R package. For stress response markers (e.g., H_2_O_2_, TBARS, 13(RS)-HPOD, Asc), the Student’s *t*-test for independent samples combined with a normality test was applied. *t*-tests with Benjamini–Hochberg false discovery rate correction were also performed for the identification of metabolites and proteins up- or downregulated during *CsRALF34* overexpression. In all statistical tests, differences with *p*-values < 0.05, <0.01, or <0.001 were considered statistically significant.

## 5. Conclusions

The results of our study on the function of the cucumber ortholog of Arabidopsis RALF34 showed that in contrast to Arabidopsis, *Cs*RALF34 did not affect lateral root initiation at all but did affect root growth. At the proteome level, the overexpression of *CsRALF34* led to increased levels of protein phosphatase 2C (PP2C) and calcium-dependent protein kinase 12 (CDPK12), i.e., regulators involved in phytohormone signaling, developmental processes, and the response to several stress factors. These effects should lead to the suppression of CDKA activity and, thus, to a block of G2/mitosis transition. Reduced levels of proliferating cell nuclear antigen and helicases are consistent with reduced mitotic activity in the basal part of the root meristem apical and might also explain the reduction in root growth. Reduced levels of NADH-dehydrogenase and succinyl CoA synthetase suggested suppression of the mitochondrial electron transport, which would be consistent with the reduced ROS levels in *CsRALF34* overexpressors. However, it is difficult to determine which one has the primary effect.

Altogether, these results show that a better understanding of RALF34-receptor interactions in non-model plants should be key to understanding the physiological role of these small signaling peptides.

## Figures and Tables

**Table 1:** Primary metabolites of *Cucumis sativus* roots demonstrating statistically significant regulation associated with the overexpression of *CsRALF34*.

Regulated Metabolites		FC *	log_2_(FC)	*p*_adjusted_	−lg(*p*)
DOWNREGULATED	*D*-mannose	0.343	−1.542	0.029	1.538
	cGMP	0.391	−1.356	0.006	2.242
	gluconic/galacturonic acid	0.648	−0.625	0.047	1.324
UPREGULATED	nicotinic acid	1.665	0.735	0.006	2.242
	adenosine	1.745	0.803	0.009	2.053
	glucose-1-phosphate	1.813	0.858	0.049	1.310
	arginine	1.825	0.868	0.020	1.703
	orotidine 5′-monophosphate	1.916	0.938	0.049	1.310
	ornithine	2.151	1.105	0.011	1.965
	6-phosphogluconic acid	2.346	1.230	0.029	1.538

* FC, fold change in the abundance of individual metabolites in the *Cucumis sativus* roots with *CsRALF34* overexpression compared with *GUS* control roots; only Benjamini–Hochberg false discovery rate correction-adjusted (*p*_adjusted_) data (*p* ≤ 0.05, FC ≥ 1.5) are presented.

**Table 2:** Top 25 differentially expressed proteins with the highest impact on the inter-group differences observed in the principal component analysis performed for 208 differentially expressed proteins.

Protein Name	Accession ^a^	Direction of Alterations ^b^	PC1	FC ^c^	*p*_adjusted_^d^	Functional Annotation	Prediction of Localization
Thioredoxin	Csa2g362500.1	up	0.084	17.8	5.3 × 10^–35^	redox homeostasis	cytosol
Glutathione peroxidase	Csa5g154200.1	up	0.081	17.2	4.4 × 10^–35^	redox homeostasis	cytosol, mitochondrion
Ribosomal protein L19	Csa4g001980.1	up	0.081	17.0	5.6 × 10^–35^	protein biosynthesis	cytosol
CHP-rich zinc finger protein-like	Csa3g850660.1	up	0.081	17.2	6.8 × 10^–35^	not assigned	cytosol
40S ribosomal protein S24	Csa2g012120.1	up	0.080	16.7	3.2 × 10^–34^	protein biosynthesis	cytosol
Carnitine operon protein CaiE	Csa1g001460.1	up	0.079	16.6	5.6 × 10^–35^	cellular respiration	mitochondrion
Wound/stress protein	Csa6g500520.1	up	0.078	16.5	4.4 × 10^–35^	not assigned	endoplasmic reticulum
Porin/voltage-dependent anion-selective channel protein	Csa6g404150.1	up	0.078	16.5	5.6 × 10^–35^	solute transport	mitochondrion
60S ribosomal protein L18a	Csa4g608120.1	up	0.077	16.4	6.8 × 10^–35^	protein biosynthesis	cytosol
Phosphofructokinase	Csa1g575110.1	up	0.077	16.0	8.7 × 10^–35^	cellular respiration	cytosol
Eukaryotic translation initiation factor 3 subunit C	Csa3g223320.1	up	0.077	16.3	1.4 × 10^–32^	protein biosynthesis	nucleus
Pleckstrin homology domain-containing family A member	Csa3g134900.1	up	0.077	16.3	5.0 × 10^–16^	lipid metabolism	plasma membrane
3-oxoacyl-[acyl-carrier-protein] synthase 3	Csa5g160250.1	up	0.076	16.3	9.0 × 10^–35^	lipid metabolism	plastid
RNA-binding protein 8A	Csa1g616290.1	up	0.076	16.1	8.3 × 10^–35^	RNA processing	nucleus
Transmembrane 9 superfamily protein member	Csa1g256720.1	up	0.075	15.3	9.5 × 10^–35^	not assigned	plasma membrane
70 kDa heat shock protein	Csa2g070310.1	down	−0.100	21.1	1.7 × 10^–35^	protein homeostasis	cytosol
Ubiquitin-like domain-containing CTD phosphatase	Csa5g587150.1	down	−0.089	18.7	4.5 × 10^–35^	protein modification	nucleus
Phloem lectin	Csa4g500830.1	down	−0.086	18.2	4.8 × 10^–35^	not assigned	plastid
AP-4 complex accessory subunit tepsin	Csa3g462690.1	down	−0.083	17.6	2.4 × 10^–34^	vesicle trafficking	Golgi apparatus
10 kDa chaperonin	Csa5g570400.1	down	−0.083	17.6	4.8 × 10^–35^	protein homeostasis	plastid
Pentatricopeptide repeat-containing protein	Csa3g840460.1	down	−0.082	17.5	1.7 × 10^–35^	not assigned	plastid
60S ribosomal protein L32	Csa1g462020.2	down	−0.082	17.3	2.0 × 10^–30^	protein biosynthesis	cytosol
Subtilisin-like serine protease	Csa5g187860.1	down	−0.081	17.4	8.3 × 10^–35^	protein homeostasis	vacuole
Protein MEMO1	Csa2g382410.1	down	−0.081	17.2	6.8 × 10^–35^	not assigned	mitochondrion
NADH dehydrogenase 1 alpha subcomplex subunit 13	Csa2g169710.1	down	−0.079	16.9	5.6 × 10^–35^	cellular respiration	mitochondrion

^a^ The identification of peptides and annotation of proteins relied on a search against amino acid sequences from *Cucumis sativus* cv. Chinese Long v2 using the SEQUEST algorithm; Mercator4 was used for protein annotation; subcellular localization of proteins was predicted with the WoLF PSORT tool. Data are available via ProteomeXchange with the identifier PXD037725. ^b^ The direction of alteration is specified as upregulated (up) or downregulated (down); ^c^ FC, fold change; ^d^ *p*_adjusted_, adjusted *p*-value.

## Data Availability

All relevant data are available within the paper and its Supplementary Materials files.

## Supplementary Materials

The following supporting information can be downloaded at: https://www.mdpi.com/article/10.3390/ijms24087654/s1.

## Abbreviations

GC-EI-Q-MS, gas chromatography-quadrupole mass spectrometry with EI ionization; MS/MS, tandem mass spectrometry; PCA, principal component analysis; RSD, relative standard deviation; TCA cycle, tricarboxylic acid cycle; ROS, reactive oxygen species; RT-qPCR, reverse transcription-quantitative polymerase chain reaction.
