# Supplementary material for: Integrative Proteomics and Metabolomics Analysis Reveals the Role of Small Signaling Peptide Rapid Alkalinization Factor 34 (RALF34) in Cucumber Roots
Source: Int J Mol Sci. 2023 Apr 21;24(8):7654. doi: 10.3390/ijms24087654 (PMC10140933; doi:10.3390/ijms24087654)
Supplement: Supplementary file 1 [file ijms-24-07654-s001.zip › Supplementary Information S1.pdf]

The following Supplementary Material is available for this article:

## Contents:

**Table S1-1.** List of qPCR primers used in this study.

**Table S1-2.** List of primers used for PCR in this study.

**Table S1-3.** Combination of primers, used for different cloning steps.

**Table S1-4.** Thermally stabile primary metabolites annotated by spectral similarity search and/or co-elution with authentic standards in *Cucumis sativus* root aqueous methanolic extracts from the roots with overexpression of *CsRALF34* or from roots of control group.

**Table S1-5.** Thermally labile primary metabolites annotated by co-elution with authentic standards and tandem mass spectrometric (MS/MS) information in *Cucumis sativus* root aqueous methanolic extracts from the roots with *CsRALF34* overexpression or from roots of control group.

**Table S1-6.** Protein extraction yields, protein concentrations in the extracts and optical densities of individual SDS-PAGE lanes corresponding to individual samples of protein extracts isolated from the *Cucumis sativus* roots with overexpression of *CsRALF34* or from roots of control group.

**Table S1-7.** Eigenvalues (loadings) obtained in the principal component analysis, in particular PC1, of differentially expressed part of *Cucumis sativus* root proteome, associated with *CsRALF34* overexpression.

**Table S1-8.** Gas chromatographic (GC) separation conditions and electron ionization-quadrupole-mass spectrometry (EI-Q-MS) settings for analysis of primary thermally stabile metabolites from *Cucumis sativus* roots with GC-EI-Q-MS.

**Table S1-9.** The conditions of ion pair-reversed phase ultrahigh performance liquid chromatographic (IP-RP-UHPLC) separation and the settings for electrospray ionization-triple quadrupole-tandem mass spectrometry (ESI-QqQ-MS/MS) used for the analysis of anionic primary thermally labile metabolites from *Cucumis sativus* root with IP-RP-UHPLC- ESI-QqQ-MS/MS.

**Table S1-10.** The conditions of ultrahigh performance liquid chromatographic (UHPLC) separation and the settings for electrospray ionization-quadrupole-time of flight mass spectrometry (ESI-QqTOF-MS) applied for the analysis of semi-polar secondary metabolites from *Cucumis sativus* root.

**Table S1-11.** Parameters of the nanoHPLC separation method employed in the nanoLC-QqTOF-MS-based proteomics experiments.

**Table S1-12.** Instrument settings applied for ESI-QqTOF-MS DDA experiments employed in the nanoLC-QqTOF-MS-based proteomics experiments.

**Table S1-13.** PEAKS Studio 10.6 parameters for database search settings.

**Figure S1-1.** Map of vectors for *CsRALF34* overexpression.

**Figure S1-2.** Biochemical characterization of *Cucumis sativus* transgenic roots overexpressing *CsRALF34*.

**Figure S1-3.** Results of hierarchical clustering with a heatmap representation of all primary metabolites.

**Figure S1-4.** Visualization of the results (A) and the corresponding statistical information (B) acquired in the t-test accomplished for the aqueous methanolic extracts obtained from the Cucumis sativus roots with CsRALF34 overexpression or from the control group.

**Figure S1-5.** Visualization of the results (A) and the corresponding statistical information (B) acquired in the fold-change (FC) analysis with the cut-off FC ≥ 1.5, accomplished for the aqueous methanolic extracts obtained from the Cucumis sativus roots with CsRALF34 overexpression or from the control group.

**Figure S1-6.** Analysis of the semi-polar secondary metabolites: post-processing of the RP-HPLC-QqTOF-MS data.

**Figure S1-7.** Electrophoregrams of total protein isolates and their tryptic hydrolysates from the Cucumis sativus roots from the control group (GUS-control) and with CsRALF34 overexpression (CsRALF34-OE).

**Figure S1-8.** Number of peptides (A), proteins (B) and protein groups (C), identified in control (GUS-control) and CsRALF34-OE (overexpression of *CsRALF34*) *Cucumis sativus* roots.

**Figure S1-9.** Principal component analysis (PCA) with a score plot, loadings plot and a biplot illustrating correlation between principal components and source variables.

**Protocol S1-1.** The full list of the reagents.

**Protocol S1-2.** Determination of Hydrogen Peroxide Contents.

**Protocol S1-3.** Determination of Lipid Peroxidation Product Contents.

**Protocol S1-4.** Determination of Lipid Hydroperoxide Contents.

**Protocol S1-5**. Determination of Ascorbic Acid Contents.

**Protocol S1-6.** Analysis of Primary Metabolites.

**Protocol S1-7.** Analysis of Semi-Polar Secondary Metabolites.

**Protocol S1-8.** Protein Isolation and Determination.

**Protocol S1-9.** Solid Phase Extraction.

# Tables

**Table S1-1.** List of qPCR primers used in this study.

| Name | Sequence 5′-3′ | Amplicon size, bp |
|---|---|---|
| CsEF1a FOR | ATGGGTAAGGAGAAGGTTCACATTAACATT | 241 |
| CsEF1a REV | CGAACTTCCACAAAGCAATATCAATT | |
| CsRALF34 FOR | CGTAGGGAAGGAGTGAAGAGGTGG | 160 |
| CsRALF34 REV | TGGATGAGGGAAGTGGTGGTGG | |
| CsGATA14 FOR | TTCCTTCAAGACCAAACCCCTGAT | 280 |
| CsGATA14 REV | CCTCCACCAGTTGTTCCTCCGAG | |
| CsGATA24 FOR | GAAGAAAATGGGAGGATCAGGAGG | 164 |
| CsGATA24 REV | ATAAAGCCATAAGAAGCAACGCTGC | |
| CsE2Fa FOR | AAGCCATCGGAGCCACTGC | 186 |
| CsE2Fa REV | GAGAGGTTTAACAACAATGGTATCAGG | |
| CsE2Fa-like FOR | GTCCATGCCGTTACGATAGTTCTCT | 163 |
| CsE2Fa-like REV | CCAATTCCCTCTAGGACATTTGTTATATC | |
| CsE2Fc FOR | GATCTCATCTTCCGCCACCG | 141 |
| CsE2Fc REV | CCGATGATACTGAAGACAACACTGAG | |
| CsE2Fe FOR | CCCTTGATGAAGCTGCTAAGCTCT | 171 |
| CsE2Fe REV | TCACTCCCAACCACCTAAACGC | |
| CsE2Fe-like FOR | GGACTCTTCTTCAAACGCTTACAGC | 192 |
| CsE2Fe-like REV | CTACACAGAATACCAACACTTTCTAAAACG | |
| CsDP FOR | GTTGCAGATGAACTTGTCGCAGA | 177 |
| CsDP REV | GCAGACCCTTCCATTGTATCTCCT | |

**Table S1-2.** List of primers used for PCR in this study.

| Name | Restriction enzyme | Sequence 5`-3` |
|---|---|---|
| CDS_CsRALF34_FOR | - | TTGAAAACCGACACTAAAAAACAAGAA |
| CDS_CsRALF34_REV | - | ATATAAAATAAGGAAATCCCCAAAACTACA |
| CDS_CsRALF34_FOR1 | KpnI | AAAGGTACCATGGCTTCCAAATCCCTCCTCTT |
| CDS_CsRALF34_REV1 | NotI | AAGCGGCCGCTCAGCGGCGGCAGCGA |
| gusA_FOR | KpnI | AAAGGTACCATGTTACGTCCTGTAGAAACCCCAAC |
| gusA_REV | NotI | AAGCGGCCGCTCATTGTTTGCCTCCCTGCTG |
| p35S_FOR | - | GCCGCCTAGAGCCAAGCTGA |
| TermAct_REV | - | CTCAAGCGAAATGGTGCGATCT |

Restriction enzyme/att sites in adaptors are underlined and given in **bold** style.

**Table S1-3.** Combination of primers, used for different cloning steps.

| Combination of primers | Application |
|---|---|
| CDS_CsRALF34_FOR/ CDS_CsRALF34_REV | PCR amplification of *CsRALF34* coding sequence using cucumber genomic DNA as a template for subsequent cloning to pJET1.2 |
| CDS_CsRALF34_FOR1/ CDS_CsRALF34_REV1 | PCR amplification of *CsRALF34* coding sequence for subsequent *Kpn*I-*Not*I cloning to pUC18-entry8 |
| gusA_FOR/gusA_REV | PCR amplification of *gusA* coding sequence for subsequent *Kpn*I-*Not*I cloning to pUC18-entry8 |
| p35S_FOR/ TermAct_REV | *p35S::CsRALF34-TermAct* and *p35S::gusA-TermAct* inserts verification by PCR in pKGW-RR-MGW vector |

**Table S1-4.** Thermally stabile primary metabolites annotated by spectral similarity search and/or co-elution with authentic standards in *Cucumis sativus* root aqueous methanolic extracts from the roots with overexpression of *CsRALF34* or from roots of control group. Metabolite analysis relied on GC-EI-Q-MS after derivatization of the lyophilized extracts with methoxyamine hydrochloride (MOA) and *N*-methyl-*N*-(trimethylsilyl)trifluoroacetamide (MSTFA).

| # | Name[a] | Derivative[b] | $t_R$ exp[c] | RI[d] | *m/z* quant[e] |
|---|---|---|---|---|---|
| 1 | Carbodiimide | 2TMS | 6.16 | 977.9 | 171 |
| 2 | Ethoxyamine | 2TMS | 6.36 | 979.9 | 119 |
| 3 | Boric acid | 3TMS | 6.5 | 988.8 | 221 |
| 4 | Ethylene glycol | 2TMS | 6.63 | 997.2 | 191 |
| 5 | Glyoxylic acid | 2TMS | 6.82 | 1007.1 | 117 |
| 6 | 1,3-Dimethoxybenzene | - | 6.88 | 1009.5 | 140 |
| 7 | 4-Hydroxypyridine | 1TMS | 7.64 | 1043.4 | 152 |
| 8 | RI_1057_Unknown | - | 7.95 | 1057.6 | 234 |
| 9 | Lactic acid | 2TMS | 8.12 | 1064.9 | 117 |
| 10 | Glycolic acid_peak1 | 2TMS | 8.42 | 1078.4 | 177 |
| 11 | Glycolic acid_peak2 | 2TMS | 8.52 | 1082.8 | 177 |
| 12 | Glycine | - | 8.68 | 1091.6 | 106 |
| 13 | Valine_peak1 | 1TMS | 8.85 | 1097.4 | 72 |
| 14 | Glycine_peak1 | 3TMS | 8.89 | 1099.6 | 116 |
| 15 | 1-Octanol | 1TMS | 9 | 1104.5 | 187 |
| 16 | *L*-Alanine_peak1 | 3TMS | 9.01 | 1105.1 | 116 |
| 17 | Valine | 1TMS | 9.01 | 1111.81 | 72 |
| 18 | *L*-Alanine_peak2 | 2TMS | 9.09 | 1108.8 | 116 |
| 19 | Hydroxylamine | 3TMS | 9.15 | 1111.9 | 249 |
| 20 | Oxalic acid_peak1 | 2TMS | 9.89 | 1148.5 | 133 |
| 21 | Oxalic acid_peak2 | 2TMS | 9.95 | 1151.4 | 220 |
| 22 | β-Lactate | 2TMS | 10.06 | 1156.6 | 177 |
| 23 | RI_1169_Unknown | 1TMS | 10.31 | 1168.9 | 89 |
| 24 | *L*-Proline_peak1 | TMS | 10.78 | 1191.9 | 70 |
| 25 | Methyl-phosphate | 2TMS | 10.82 | 1193.8 | 241 |
| 26 | Isoleucine_peak1 | 1TMS | 10.83 | 1194 | 86 |
| 27 | Malonic acid_peak 1 | 2TMS | 11.33 | 1218.5 | 130 |
| 28 | Malonic acid_peak 2 | 2TMS | 11.43 | 1223.5 | 174 |
| 29 | *DL*-Glyceraldehyde | 2TMS, MEOX | 11.43 | 1224 | 133 |
| 30 | Valine_peak2 | 1TMS | 11.56 | 1230.3 | 144 |
| 31 | *DL*-Norvaline | 2TMS | 11.64 | 1234.06 | 144 |
| 32 | *DL*-Glyceraldehyde | 2TMS, MEOX | 11.68 | 1235.9 | 133 |
| 33 | unknown_RI_1240 | - | 11.83 | 1243.49 | 156 |
| 34 | Diethylene glycol | 2TMS | 11.84 | 1244.1 | 117 |
| 35 | Urea | 2TMS | 12.37 | 1269.8 | 189 |
| 36 | Benzoic acid | 1TMS | 12.42 | 1272.6 | 179 |

| # | Name[a] | Derivative[b] | $t_R$ exp[c] | RI[d] | *m/z* quant[e] |
|---|---|---|---|---|---|
| 37 | Serine | 2TMS | 12.6 | 1281.2 | 116 |
| 38 | Ethanamine | 3TMS | 12.67 | 1284.5 | 174 |
| 39 | Octanoic acid | 2TMS | 12.84 | 1292.1 | 201 |
| 40 | Glycerol | 3TMS | 12.87 | 1294.2 | 205 |
| 41 | *L*-Leucine | 2TMS | 12.87 | 1294.3 | 158 |
| 42 | Phosphate | 3TMS | 12.88 | 1294.7 | 299 |
| 43 | *L*-Isoleucine_peak2 | 2TMS | 13.3 | 1311.2 | 158 |
| 44 | *L*-Proline_peak2 | 2TMS | 13.38 | 1313.7 | 142 |
| 45 | *L*-Threonine_peak1 | 2TMS | 13.43 | 1315.7 | 219 |
| 46 | Nicotinic acid | TMS | 13.55 | 1319.9 | 180 |
| 47 | Glycine_peak2 | 2TMS | 13.58 | 1320.8 | 248 |
| 48 | Maleic acid | 2TMS | 13.68 | 1324.3 | 245 |
| 49 | Succinic acid_peak 1 | 2TMS | 13.7 | 1325 | 247 |
| 50 | Butenedioic acid | - | 13.82 | 1329.3 | 101 |
| 51 | Succinic acid_peak 2 | 2TMS | 13.91 | 1332.1 | 247 |
| 52 | RI_1336_unknown | - | 14.05 | 1337.1 | 57 |
| 53 | *D*-Glyceric acid | 3TMS | 14.18 | 1341.6 | 189 |
| 54 | Uracil | 2TMS | 14.41 | 1349.9 | 241 |
| 55 | Citraconic acid | 2TMS | 14.66 | 1358.6 | 245 |
| 56 | RI_1361_Unknown | - | 14.73 | 1361 | 188 |
| 57 | Fumaric acid | 2TMS | 14.75 | 1361.5 | 245 |
| 58 | RI_1364_unknown | - | 14.81 | 1363.4 | 188 |
| 59 | *L*-Homoserine | 2TMS | 14.85 | 1365.1 | 146 |
| 60 | $\alpha$-methyl-serine_peak1 | 3TMS | 14.85 | 1366.8 | 116 |
| 61 | Nonanoic acid | 1TMS | 14.97 | 1369.2 | 215 |
| 62 | 3-Aminopropionitrile | 2TMS | 15 | 1361.3 | 245 |
| 63 | Propanedioic acid | - | 15.42 | 1385 | 131 |
| 64 | 1,1,1-Tris(hydroxymethyl)propane | 3TMS | 15.48 | 1386.1 | 191 |
| *65* | *L*-Threonine_peak2 | 3TMS | 15.48 | 1387.1 | 219 |
| 66 | $\alpha$-methyl-serine_peak2 | 3TMS | 15.6 | 1391.8 | 116 |
| 67 | Homocysteine | 3TMS | 15.88 | 1401.2 | 234 |
| 68 | Glutaric acid (Pentanedioic acid)_1 | 2TMS | 15.95 | 1403.9 | 261 |
| 69 | 3-Deoxytetronic acid | 3TMS | 16.14 | 1410.7 | 219 |
| 70 | *L*-Methionine | TMS | 16.2 | 1410.9 | 131 |
| 71 | β-Alanine | 3TMS | 16.29 | 1416.1 | 248 |
| 72 | *L*-Aspartic acid_peak 1 | 2TMS | 16.32 | 1417.2 | 116 |
| 73 | *L*-Homoserine | 3TMS | 16.75 | 1433.3 | 218 |
| 74 | Erythrose_peak 1 | 3TMS, MEOX | 16.99 | 1442.1 | 205 |
| 75 | Decanoic acid | 1TMS | 17.09 | 1445.9 | 229 |
| 76 | Erythrose_peak 2 | 3TMS, MEOX | 17.17 | 1448.5 | 205 |
| 77 | *D*(-)-Erythrulose_peak1 | 1MEOX_3TMS | 17.21 | 1450.3 | 173 |
| 78 | *D*(-)-Erythrulose_peak2 | 1MEOX_3TMS | 17.39 | 1456.8 | 173 |
| 79 | *L*-Glutamine | 3TMS | 17.4 | 1456.9 | 91 |
| 80 | 4-Ketoglucose | 4TMS | 17.4 | 1457 | 103 |

| # | Name[a] | Derivative[b] | $t_R$ exp[c] | RI[d] | *m/z* quant[e] |
|---|---|---|---|---|---|
| 81 | L-Hydroxyproline | 2TMS | 17.62 | 1465.2 | 158 |
| 82 | Malic acid | 3TMS | 17.85 | 1472.9 | 233 |
| 83 | meso-Erythritol | 2TMS | 17.9 | 1475.6 | 217 |
| 84 | Salicylic acid | 2TMS | 18.02 | 1479.8 | 267 |
| 85 | RI_1478_unknown | - | 18.05 | 1480.9 | 58 |
| 86 | Adipic acid | 2TMS | 18.06 | 1481.3 | 205 |
| 87 | Hexadecanoic acid_peak2 | - | 18.13 | 1484 | 170 |
| 88 | 2-Phenylglycine | 2TMS | 18.24 | 1486 | 178 |
| 89 | *L*-Aspartic acid_peak 2 | 3TMS | 18.27 | 1490.2 | 232 |
| 90 | *L*-Proline_peak3 | 2TMS | 18.3 | 1490.3 | 156 |
| 91 | *L*-Methionine | 2TMS | 18.32 | 1490.9 | 128 |
| 92 | $\gamma$-Aminobutyric acid (=GABA) | 3TMS | 18.42 | 1495.7 | 174 |
| 93 | Pyrogallol | 3TMS | 18.56 | 1499.6 | 239 |
| 94 | *D*-Erythronic acid | 4TMS | 18.63 | 1504.9 | 292 |
| 95 | Phenylalanine | - | 18.86 | 1521.6 | 120 |
| 96 | *L*-Cysteine | 3TMS | 18.96 | 1527.9 | 57 |
| 97 | *L*-Threonic acid | 4TMS | 19.19 | 1544.8 | 292 |
| 98 | Glutaric acid/Pentanedioic acid_2 | 2TMS | 19.21 | 1546.2 | 221 |
| 99 | Dodecanol | 1TMS | 19.29 | 1552.1 | 243 |
| 100 | $\alpha$-Hydroxyglutaric acid | 3TMS | 19.39 | 1559.4 | 247 |
| 101 | Glutaric acid/Pentanedioic acid_3 | 2TMS | 19.48 | 1565.7 | 198 |
| 102 | RI_1571_Unknown (related to proline) | - | 19.55 | 1571 | 142 |
| 103 | RI_1588_Unknown | - | 19.79 | 1588.8 | 239 |
| 104 | Asparagine | 2TMS | 19.87 | 1593.2 | 159 |
| 105 | RI_1598_unknown | - | 19.94 | 1598.9 | 57 |
| 106 | Cinnamic acid | 1TMS | 19.96 | 1600.5 | 131 |
| 107 | Heptanedioate/Pimelic acid | 2TMS | 20.12 | 1608.6 | 75 |
| 108 | Glutamic acid | 3TMS | 20.26 | 1615.7 | 246 |
| 109 | L(-)-Phenylalanine | 2TMS | 20.33 | 1619.7 | 218 |
| 110 | L-(+)-Tartaric acid (=L-Threaric acid) | 4TMS | 20.56 | 1631.7 | 292 |
| 111 | D(+)- Xylose_peak1 | 4TMS, MEOX | 20.57 | 1632.3 | 307 |
| 112 | RI1640_Unknown | - | 20.71 | 1639.8 | 229 |
| 113 | Vanillin | 1MEOX_1TMS | 20.83 | 1645.9 | 223 |
| 114 | Pyrophosphate | 4TMS | 20.98 | 1654.1 | 451 |
| 115 | Dodecanoic acid | 1TMS | 21.16 | 1663.3 | 257 |
| 116 | *D*(+)- Xylose_peak2 | 4TMS, MEOX | 21.19 | 1665.2 | 307 |
| 117 | d-Ribose | 1TMS | 21.21 | 1665.8 | 307 |
| 118 | RI_1680_C5-sugar alcohol | - | 21.49 | 1680.6 | 307 |
| 119 | RI_1685_unknown | - | 21.56 | 1684.4 | 221 |
| 120 | RI_1688_hydroxyfatty acid | - | 21.64 | 1688.6 | 97 |
| 121 | l-(-)-Arabitol | 5TMS | 21.68 | 1690.7 | 307 |
| 122 | RI_1693_C5-sugar alcohol | - | 21.73 | 1693.2 | 217 |
| 123 | Suberic acid | TMS | 21.79 | 1696.6 | 303 |
| 124 | *D*-glyceraldehyde-3-phosphate | 1MEOX_3TMS | 21.88 | 1701.3 | 328 |

| # | Name[a] | Derivative[b] | $t_R$ exp[c] | RI[d] | *m/z* quant[e] |
|---|---|---|---|---|---|
| | (gap)_peak1 | | | | |
| 125 | *L*-Rhamnose_peak1 | nTMS | 21.94 | 1704.6 | 160 |
| 126 | *D*-glyceraldehyde-3-phosphate (gap)_peak2 | 1MEOX_3TMS | 21.98 | 1706.7 | 328 |
| 127 | Arabinose | 1MEOX_4TMS | 22.01 | 1708.7 | 307 |
| 128 | *L*-Rhamnose_peak2 | nTMS | 22.01 | 1708.7 | 160 |
| 129 | D-glyceraldehyde-3-phosphate (gap)_peak3 | 1MEOX_3TMS | 22.19 | 1718.9 | 328 |
| 130 | Putrescine (=1.4-butanediamine) | 1MEOX_4TMS | 22.28 | 1723.9 | 174 |
| 131 | Ribitol | 5TMS | 22.29 | 1724 | 205 |
| 132 | RI_1729_unknown | - | 22.36 | 1728.2 | 57 |
| 133 | Ribonic acid | 3TMS | 22.52 | 1736.7 | 333 |
| 134 | Dihydroxyacetone phosphate dilithium salt_peak1 | 1MEOX_3TMS | 22.59 | 1740.8 | 315 |
| 135 | Ornithine hydrochloride anhydrous_peak1 | 3TMS | 22.62 | 1742 | 174 |
| 136 | cys-Aconitic acid | 3TMS | 22.72 | 1747.7 | 333 |
| 137 | RI_1748_C5-sugar derived acid | - | 22.73 | 1748.4 | 292 |
| 138 | Dihydroxyacetone phosphate_peak2 | 1MEOX_3TMS | 22.75 | 1749.5 | 315 |
| 139 | Glycerol-alpha-phosphate | 4TMS | 22.82 | 1753.7 | 299 |
| 140 | RI_1759_C5-sugar | - | 22.92 | 1759.3 | 191 |
| 141 | Vanillic acid | 2TMS | 22.96 | 1761.3 | 297 |
| 142 | RI_1771_unknown_phosphate | - | 23 | 1771.9 | 299 |
| 143 | RI_1766_C5-sugar derived acid | - | 23.05 | 1766.5 | 333 |
| 144 | RI_1770_Amine | - | 23.12 | 1770.3 | 174 |
| 145 | RI_1784_unknown | - | 23.35 | 1783.3 | 174 |
| 146 | Terephthalic acid | 2TMS | 23.37 | 1784.1 | 295 |
| 147 | 1,4-Benzenedicarboxylic acid | 2TMS | 23.42 | 1786.9 | 333 |
| 148 | RI_1788_C5-sugar derived acid | - | 23.45 | 1788.4 | 333 |
| 149 | *DL*-Isocitric acid | 4TMS | 23.59 | 1796.19 | 273 |
| 150 | Shikimic acid | 4TMS | 23.6 | 1796.08 | 255 |
| 151 | *D*(-)-Phospho-glyceric acid (gip) | 4TMS | 23.65 | 1799.4 | 299 |
| 152 | Ornithine hydrochloride anhydrous_peak2 | 4TMS | 23.74 | 1803.1 | 174 |
| 153 | 3,4-dihydroxybenzoic acid | nTMS | 23.89 | 1807.7 | 370 |
| 154 | RI_1823_Amino acid | - | 24.25 | 1823.8 | 156 |
| 155 | RI_1856_C5-6-sugar alcohol | - | 24.29 | 1825.7 | 215 |
| 156 | Quinic acid | 5TMS | 24.34 | 1827.4 | 345 |
| 157 | Adenine | TMS | 24.45 | 1831.5 | 165 |
| 158 | Myristic acid | nTMS | 24.47 | 1832.6 | 117 |
| 159 | *D*-Fructose_peak1 | 1MEOX_5TMS | 24.61 | 1838 | 307 |
| 160 | RI_1841_C6-sugar acid | - | 24.68 | 1841.2 | 275 |
| 161 | *D*-Fructose_peak2 | 1MEOX_5TMS | 24.78 | 1845 | 361 |
| 162 | *D*-Mannose_peak1 | 1MEOX_5TMS | 24.86 | 1848.3 | 319 |

| # | Name[a] | Derivative[b] | $t_R$ exp[c] | RI[d] | *m/z* quant[e] |
|---|---|---|---|---|---|
| 163 | *D*-(+)-Gluconic acid δ-lactone (=gluconolactone) | 4TMS | 24.93 | 1851.1 | 319 |
| 164 | *D*-Mannose_peak2 | 1MEOX_5TMS | 25.01 | 1857.7 | 319 |
| 165 | *D*-(+)-Galactose | 1MEOX_5TMS | 25.02 | 1854.5 | 319 |
| 166 | Erythrose-4-phosphate sodium_peak1 | 1MEOX_5TMS | 25.29 | 1865.5 | 357 |
| 167 | *L*-Histidine monohydrochloride monohydrate | 3TMS | 25.39 | 1869.3 | 154 |
| 168 | Erythrose-4-phosphate sodium_peak2 | 1MEOX_5TMS | 25.48 | 1873.1 | 357 |
| 169 | *D*-Mannitol | 6TMS | 25.53 | 1874.8 | 319 |
| 170 | Lysinee_peak1 | 3TMS | 25.56 | 1876.1 | 174 |
| 171 | *D*-Sorbitol(d-Glucitol) | 6TMS | 25.61 | 1878.1 | 319 |
| 172 | Dulcitol (=Galactitol) | 6TMS | 25.73 | 1883 | 319 |
| 173 | *D*-(+)-Galacturonic acid_peak1 | 1MEOX_5TMS | 25.79 | 1885.4 | 333 |
| 174 | Lysine_peak2 | 4TMS | 25.79 | 1885.4 | 174 |
| 175 | *D*-Glucuronic acid | 1MEOX_4TMS | 25.91 | 1890.2 | 333 |
| 176 | *D*-Glucose_peak1 | MEOX _5TMS | 25.94 | 1891.5 | 205 |
| 177 | *L*-Ascorbic acid_peak2 | 4TMS | 26.04 | 1895.2 | 332 |
| 178 | *D*-(-)-Isoascorbic acid | 4TMS | 26.13 | 1898.9 | 332 |
| 179 | *D*-(+)-Galacturonic acid monohydrate_peak2 | 1MEOX_5TMS | 26.13 | 1898.9 | 333 |
| 180 | cys-Ferulic acid | 2TMS | 26.28 | 1914.8 | 338 |
| 181 | *L*-Iditol | 6TMS | 26.29 | 1915.7 | 103 |
| 182 | RI_1919_Phosphocarbohydrate | - | 26.37 | 1919.5 | 299 |
| 183 | 5-Keto-D-Gluconic acid | nTMS | 26.4 | 1927.9 | 149 |
| 184 | *D*-Glucose_peak2 | 1MEOX_5TMS | 26.43 | 1936.9 | 205 |
| 185 | 4-Coumaric acid | nTMS | 26.57 | 1947.9 | 219 |
| 186 | *D*-(+)-Galacturonic acid_peak3 | 1MEOX_5TMS | 26.59 | 1949.4 | 333 |
| 187 | RI_1951_C6_sugar | - | 26.62 | 1951.9 | 217 |
| 188 | Gulonic acid, γ-lactone | 4TMS | 26.66 | 1953 | 189 |
| 189 | *D*-Glucaric acid | 6TMS | 26.83 | 1976.8 | 333 |
| 190 | Pyridoxine | 3TMS | 26.91 | 1986.4 | 293 |
| 191 | *L*-Ascorbic acid_peak2 | 4TMS | 27.26 | 1949.1 | 332 |
| 192 | Palmitoleic acid_1 | nTMS | 27.27 | 2015.6 | 129 |
| 193 | Gallic acid | 4TMS | 27.29 | 1950.9 | 281 |
| 194 | Mucic acid (=Galactaric acid) | 6TMS | 27.34 | 2020.2 | 333 |
| 195 | RI_2034_Unknown | - | 27.56 | 2034.4 | 203 |
| 196 | Palmitic acid | TMS | 27.71 | 2044.5 | 313 |
| 197 | Myo-inositol | 6TMS | 28.08 | 2068.5 | 318 |
| 197 | RI_2092_C6-sugar derived acid | - | 28.45 | 2092.3 | 245 |
| 198 | *D*-Ribose-5-phosphate | 1MEOX_5TMS | 28.47 | 2094 | 315 |
| 199 | RI_2103_unknown | - | 28.51 | 2096.6 | 174 |
| 200 | RI_2108_Unknown | - | 28.68 | 2107.8 | 299 |
| 201 | *N*-Acetyl glucosamine_1 | nTMS | 28.71 | 2109.3 | 205 |
| 202 | *N*-acetyl-d-hexosamine 1 | nTMS | 28.8 | 2114.81 | 319 |

| # | Name[a] | Derivative[b] | $t_R$ exp[c] | RI[d] | *m/z* quant[e] |
|---|---|---|---|---|---|
| 203 | RI_2122_unknown | - | 28.89 | 2121.4 | 221 |
| 204 | Hexanedioic acid | nTMS | 28.95 | 2124.6 | 131 |
| 205 | trans-Caffeic acid (=2-propenoic acid) | 3TMS | 29.07 | 2133.2 | 219 |
| 206 | Heptadecanoic acid | nTMS | 29.19 | 2141 | 204 |
| 207 | Octadecanol | nTMS | 29.39 | 2153.8 | 327 |
| 208 | *α-D*-gluco-hexopyranose | nTMS | 29.73 | 2175.7 | 204 |
| 209 | Phytanic acid | nTMS | 29.82 | 2181.7 | 120 |
| 210 | RI_2185_C5-6-sugar-phosphate | - | 29.88 | 2185.4 | 357 |
| 211 | N-*α*-acetyl-*L*-Lysine | - | 29.99 | 2193.6 | 174 |
| 212 | RI_2194_inositol-phosphate | - | 30 | 2194 | 299 |
| 213 | *DL*-Tryptophan | 2TMS | 30.03 | 2195.3 | 202 |
| 214 | Tryptamine_peak1 | 2TMS | 30.06 | 2197.1 | 174 |
| 215 | 9,12-Octadecadienoic acid | TMS | 30.21 | 2208 | 341 |
| 216 | Tryptamine_peak2 | 3TMS | 30.25 | 2210.3 | 174 |
| 217 | Oleic acid |  | 30.3 | 2214.2 | 341 |
| 218 | Heptadecanoic acid | TMS | 30.46 | 2225.3 | 204 |
| 219 | RI_2242_unknown phosphate | - | 30.69 | 2241.6 | 299 |
| 220 | Stearic acid_peak1 | TMS | 30.69 | 2241.7 | 341 |
| 221 | RI_2260_unknown | - | 30.91 | 2257 | 290 |
| 222 | Hexadecanoic acid butyl ester | TMS | 30.93 | 2258.6 | 87 |
| 223 | Flavone | TMS | 30.94 | 2259.7 | 221 |
| 224 | RI_2267_unknown | - | 31.05 | 2267.4 | 165 |
| 225 | RI_2274_unknown | - | 31.14 | 2273.8 | 151 |
| 226 | Glycerol-3-galactoside | TMS | 31.22 | 2279.2 | 117 |
| 227 | Fructose-6-phosphate | 1MEOX_6TMS | 31.33 | 2287.3 | 315 |
| 228 | Mannose-1-phosphate_peak1 | 1MEOX_6TMS | 31.5 | 2299.4 | 387 |
| 229 | Mannose-1-phosphate_peak2 | 1MEOX_6TMS | 31.75 | 2317 | 387 |
| 230 | RI_2318_C5-6-sugar-phosphate | - | 31.77 | 2318 | 299 |
| 231 | RI_2323_unknown sugar | - | 31.84 | 2322.9 | 319 |
| 232 | *D*-Glucose 6-phosphate | 6TMS | 31.93 | 2329.4 | 204 |
| 233 | Sinigrin | nTMS | 32.08 | 2340.6 | 17 |
| 234 | Lysine | nTMS | 32.12 | 2342.7 | 204 |
| 235 | Glucose-6-phosphate_peak1 | 1MEOX_6TMS | 32.37 | 2360.6 | 387 |
| 236 | *D*-Glucuronic acid | - | 32.38 | 2360.8 | 103 |
| 237 | Glucose-6-phosphate_peak2 | 1MEOX_6TMS | 32.54 | 2372.6 | 387 |
| 238 | RI_2377_Carbohydrate | - | 32.6 | 2377.2 | 204 |
| 239 | Myo-inositol | nTMS | 32.72 | 2385.8 | 239 |
| 240 | Hexanedioic acid | 2TMS | 32.74 | 2387.5 | 129 |
| 241 | RI_2395_unknown | - | 32.85 | 2394.9 | 221 |
| 242 | Nonadecanoic acid | TMS | 33.14 | 2416.1 | 130 |
| 243 | RI_2438_Carbohydrate | - | 33.43 | 2438.6 | 204 |
| 244 | RI_2465_unknown | - | 33.78 | 2465.4 | 87 |
| 245 | Biotin | nTMS | 33.95 | 2478.7 | 446 |
| 246 | Monoferuloylglycerol | TMS | 34.05 | 2486.4 | 249 |

| # | Name[a] | Derivative[b] | $t_R$ exp[c] | RI[d] | *m/z* quant[e] |
|---|---|---|---|---|---|
| 247 | RI_2493_Disacharide | - | 34.14 | 2492.8 | 361 |
| 248 | Ribulose-1.5-biphosphate_peak1 | 1MEOX_5TMS | 34.2 | 2498.2 | 357 |
| 249 | Ribulose-1.5-biphosphate_peak2 | 1MEOX_5TMS | 34.57 | 2525.8 | 357 |
| 250 | RI_2530_Disacharide | - | 34.63 | 2530.2 | 361 |
| 251 | Melibiose | 8TMS | 34.7 | 2535.8 | 149 |
| 252 | RI_2545_unknown | - | 34.82 | 2545.3 | 165 |
| 253 | 1-Docosanol | nTMS | 34.84 | 2546.6 | 383 |
| 254 | Salicyl alcohol-b-glucoside | nTMS | 35.01 | 2559.6 | 361 |
| 255 | Monopalmitoylglycerol | 2TMS | 35.29 | 2581.1 | 371 |
| 256 | Sucrose | 8TMS | 35.62 | 2606.8 | 361 |
| 257 | Lactose_peak1 | nTMS | 36.2 | 2654.4 | 361 |
| 258 | RI_2662_Disacharide | - | 36.29 | 2662.5 | 361 |
| 259 | RI_2670_Disacharide | - | 36.39 | 2670 | 361 |
| 260 | Lactose_peak2 | nTMS | 36.42 | 2672.7 | 361 |
| 261 | RI_2677_Disacharide | - | 36.47 | 2676.6 | 361 |
| 262 | Fructose-1.6-biphosphate_peak1 | 1MEOX_6TMS | 36.61 | 2688.3 | 315 |
| 263 | Fructose-1.6-biphosphate_peak2 | 7TMS | 36.76 | 2700.8 | 315 |
| 264 | *D*-(+)-Maltose monohydrate_peak1 | 1MEOX_8TMS | 36.87 | 2709.2 | 361 |
| 265 | *D*-(+)-Trehalose $\alpha,\alpha'$ dihydrate | 8TMS | 36.91 | 2713.2 | 361 |
| 266 | Turanose_peak1 | 8TMS | 37.05 | 2724.2 | 361 |
| 267 | Melibiose_peak1 | 8TMS | 37.13 | 2730.6 | 361 |
| 268 | *D*-(+)-Maltose monohydrate_peak2 | 1MEOX_8TMS | 37.2 | 2736.6 | 361 |
| 269 | Turanose_peak2 | 1MEOX_8TMS | 37.4 | 2752.4 | 307 |
| 270 | RI_2759_lysolipid derivate | - | 37.47 | 2759 | 311 |
| 271 | Stearic acid_peak2 | 2TMS | 37.65 | 2773.8 | 399 |
| 272 | RI_2786_Disaccharide | - | 37.8 | 2785.9 | 361 |
| 273 | RI_2793_unknown | - | 37.87 | 2791.9 | 221 |
| 274 | Squalene | nTMS | 38.1 | 2811.5 | 69 |
| 275 | RI_2813_Disacharide | - | 38.13 | 2813.8 | 361 |
| 276 | Turanose_peak3 | 7TMS | 38.23 | 2822.7 | 361 |
| 277 | RI_2835_Disacharide | - | 38.37 | 2835 | 361 |
| 278 | Melibiose_peak2 | 8TMS | 38.37 | 2835.6 | 361 |
| 279 | Isomaltose_peak1 | 1MEOX_8TMS | 38.45 | 2842.8 | 361 |
| 280 | RI_2849_unknown | | 38.52 | 2849 | 165 |
| 281 | Isomaltose_peak2 | 1MEOX_8TMS | 38.83 | 2876 | 361 |
| 282 | Galactinol_peak1 | | 39.34 | 2920.8 | 221 |
| 283 | RI_2946_Disacharide | - | 39.63 | 2945.9 | 361 |
| 284 | Galactinol_peak2 | 9TMS | 39.67 | 2950.1 | 204 |
| 285 | RI_2975_Disaccharide | - | 39.76 | 2975 | 204 |
| 286 | Melibiose_peak2 | 8TMS | 40.11 | 2985.1 | 361 |
| 287 | RI_3005_Galactinol derivate | - | 40.29 | 3005.2 | 204 |
| 288 | RI_3037_Carbohydrate-phosphate | - | 40.64 | 3037.4 | 299 |
| 289 | RI_3064_unknown | - | 40.92 | 3064.1 | 204 |
| 290 | RI_3069_Unknown | - | 40.98 | 3069.5 | 375 |

| # | Name[a] | Derivative[b] | $t_R$ exp[c] | RI[d] | *m/z* quant[e] |
|---|---|---|---|---|---|
| 291 | RI_3104_unknown | - | 41.35 | 3104.8 | 482 |
| 292 | RI_3147_unknown | - | 41.8 | 3147.1 | 204 |
| 293 | RI_3182_unknown | - | 42.18 | 3182.4 | 204 |
| 294 | Octacosanoic acid | nTMS | 42.67 | 3230.8 | 117 |
| 295 | RI_3249_unknown | - | 42.86 | 3249.8 | 496 |
| 296 | RI_3294_Trisacharide | - | 43.3 | 3294 | 427 |
| 297 | beta-Sitosterol | 1TMS | 43.62 | 3325.6 | 396 |
| 298 | *α-D*-gluco-hexopyranose | 8TMS | 43.78 | 3341.7 | 361 |
| 299 | RI_3378_Trisacharide | - | 44.15 | 3378.6 | 204 |
| 300 | Erlose | 11TMS | 44.16 | 3380.1 | 361 |
| 301 | RI_3409_unknown | - | 44.45 | 3409.3 | 204 |
| 302 | RI_3451_Trisacharide | - | 44.85 | 3451 | 204 |
| 303 | Maltose_peak3 | 8TMS | 45.31 | 3498.9 | 361 |
| 304 | Turanose_peak4 | 7TMS | 45.51 | 3519.8 | 361 |
| 305 | RI_3533_unknown | - | 45.65 | 3533.6 | 452 |
| 306 | RI_3563_unknown | - | 46 | 3570.2 | 204 |
| 307 | RI_3570_Unknown | - | 46 | 3570.1 | 647 |
| 308 | Maltotriose | 11TMS | 46.09 | 3578.6 | 204 |
| 309 | RI_3626_unknown | - | 46.6 | 3626.7 | 204 |
| 310 | RI_3644_Trisacharide | - | 46.81 | 3644.2 | 204 |
| 311 | RI_3679_Trisacharide | - | 47.22 | 3679.8 | 204 |
| 312 | RI_3721_unknown | - | 47.71 | 3721.7 | 204 |
| 313 | RI_3723_unknwon | - | 47.73 | 3722.9 | 340 |
| 314 | RI_3819_Trisacharide | - | 48.93 | 3819.2 | 204 |
| 315 | RI_3832_Trisacharide | - | 49.14 | 3832.3 | 204 |
| 316 | RI_3918_peak_340_unknown | - | 50.5 | 3918.5 | 340 |
| 317 | RI_4043_Trisacharide | - | 52.48 | 4043.3 | 204 |
| 318 | RI_4160_unknown | - | 54.33 | 4160.1 | 340 |
| 319 | RI_4168_unknown | - | 54.33 | 4168.4 | 361 |
| 320 | RI_4247_Trisacharide | - | 55.54 | 4247.7 | 361 |
| 321 | RI_4250_Trisacharide | - | 55.56 | 4250.2 | 361 |

[a]All metabolites are arranged in order of increasing retention times ($t_R$). Metabolites having the same name and _1H, _1L, 2H, and 2L designations are interpreted as oxime isomers of sugar MeOx derivatives. In the abbreviations _1H, _1L, 2H, and _2L, the numbers denote the peak number of the metabolite and the letters denote the relative peak height L (low) or H (high) of the corresponding peak compared with the peak height of the second isomer of the metabolite.

Unidentified metabolites are labeled with the word *Unknown*, and their annotation contains the retention index (RI). The name of metabolites annotated to a certain chemical class (without exact annotated structure) also begins with RI, followed by the name of the corresponding chemical class. Annotation to specific chemical classes was confirmed by the presence of characteristic signals (*m/z* values) specific for the corresponding chemical class (for example, C5-6-sugar-phosphate - *m/z* 299, 315 and 387; disaccharide - *m/z* 361, 204 and 319; galactinol derivative - *m/z* 204, 361 and 433).

[b]The numbers and types of derivatization groups attached to the identified metabolites: TMS - trimethylsilyl group, MEOX - methyloxime group.
[c]Retention time of the metabolite,
[d]Retention index of the metabolite;
[e]The *m/z* value of the most characteristic ion in the EI spectrum (quantifier), from which the extracted ion chromatogram was reconstructed and the peak area integration was accomplished at the given $t_R$.

**Table S1-5.** Thermally labile primary metabolites annotated by co-elution with authentic standards and tandem mass spectrometric (MS/MS) information in *Cucumis sativus* root aqueous methanolic extracts from the roots with *CsRALF34* overexpression or from roots of control group. Metabolite analysis relied on the ion pair-reversed phase high-performance liquid chromatography coupled online to the triple quadrupole tandem mass spectrometer (RP-IP-HPLC-QqQ-MS/MS) without any derivatization.

| # | Metabolite | $t_R$ (min)[a] | Q1 (*m*/*z*)[б] | Q3 (*m*/*z*)[в] |
|---|---|---|---|---|
| 1 | histidine | 0.79 | 154.1 | 93.0 |
| 2 | proline | 0.80 | 114.1 | 86.0 |
| 3 | leucine + isoleucine | 0.93 | 261.3 | 130.2 |
| 4 | creatine | 0.50 | 130.1 | 88.1 |
| 5 | glutamine | 0.79 | 145.1 | 108.9 |
| 6 | ornithine | 0.73 | 131.1 | 82.9 |
| 7 | allantoin | 0.82 | 157.0 | 97.2 |
| 8 | γ-aminobutyric acid | 0.70 | 102.1 | 84.0 |
| 9 | alanine | 0.80 | 88.0 | 41.9 |
| 10 | citrulline | 0.80 | 174.1 | 131.0 |
| 11 | *L*-cysteine | 1.11 | 120.0 | 79.8 |
| 12 | lysine | 0.80 | 145.1 | 99.0 |
| 13 | arginine | 0.77 | 173.1 | 131.0 |
| 14 | serine | 0.78 | 104.0 | 74.0 |
| 15 | valine_1 | 0.80 | 233.3 | 116.0 |
| 16 | valine_2 | 0.80 | 116.1 | 70.0 |
| 17 | *L*-dicysteine | 1.00 | 239.0 | 120.0 |
| 18 | glyoxilic acid | 0.89 | 73.0 | 45.0 |
| 19 | methionine | 0.89 | 148.0 | 47.0 |
| 20 | uridine | 0.88 | 243.1 | 109.9 |
| 21 | glycine | 0.79 | 74.0 | 74.0 |
| 22 | threonine | 0.79 | 118.1 | 73.9 |
| 23 | *S*-adenosyl-*L*-homocysteine | 0.84 | 383.1 | 133.9 |
| 24 | asparagine | 0.83 | 131.0 | 87.1 |
| 25 | sucrose | 0.85 | 341.1 | 89.0 |
| 26 | dehydroascorbic acid | 0.88 | 173.0 | 127.0 |
| 27 | cytidine | 0.87 | 242.1 | 108.9 |
| 28 | *P*-choline | 0.86 | 242.1 | 108.9 |
| 29 | tyrosine | 0.90 | 180.1 | 118.9 |
| 30 | guanosine | 1.32 | 282.1 | 149.9 |
| 31 | 2'-deoxyguanosine | 1.44 | 266.1 | 150.0 |
| 32 | phenylalanine | 1.50 | 164.1 | 103.0 |
| 33 | glucopyranonic acid/ | 2.40 | 193.0 | 113.0 |
| 34 | galactopyranuronic acid | 2.40 | 193.0 | 113.0 |
| 35 | glutamic acid | 2.44 | 146.0 | 102.0 |
| 36 | adenosine | 2.41 | 266.1 | 133.9 |
| 37 | ribonic acid | 2.57 | 165.0 | 75.0 |
| 38 | *D*-galactonic acid/*D*-gluconic acid | 2.47 | 195.1 | 129.0 |

| # | Metabolite | $t_R$ (min)[a] | Q1 (*m/z*)[b] | Q3 (*m/z*)[c] |
|---|---|---|---|---|
| 39 | glucosamine 6-phosphate | 2.40 | 258.0 | 97.0 |
| 40 | dihydroorotic acid | 2.52 | 157.0 | 112.7 |
| 41 | glucolate | 2.56 | 75.0 | 47.0 |
| 42 | shikimic acid | 2.48 | 173.0 | 92.9 |
| 43 | aspartic acid | 2.45 | 132.0 | 88.0 |
| 44 | quinic acid | 2.50 | 191.1 | 85.0 |
| 45 | uric acid | 2.58 | 167.0 | 124.0 |
| 46 | 3-ureidopropionic acid/3-(carbamoylamino)propanoic acid | 2.56 | 131.0 | 87.9 |
| 47 | 3-dehydroxyshikimic acid | 2.59 | 171.0 | 127.0 |
| 48 | ascorbic acid | 2.50 | 175.0 | 115.0 |
| 49 | glucosamine 1-phosphate | 2.50 | 258.0 | 78.9 |
| 50 | tryptophan | 2.54 | 203.1 | 116.2 |
| 51 | chloride | 2.85 | 35.0 | 35.0 |
| 52 | lactic acid | 3.12 | 89.0 | 42.9 |
| 53 | glutathione | 3.80 | 306.1 | 143.0 |
| 54 | phosphate | 3.73 | 96.9 | 78.9 |
| 55 | nicotinamide adenine dinucleotide | 4.44 | 662.1 | 540.1 |
| 56 | adenosine 2′,3'-cyclic mono-phosphate | 6.79 | 328.0 | 134.0 |
| 57 | orotic acid | 4.11 | 155.0 | 110.7 |
| 58 | fructose 6-phosphate/glucose 6-phosphate | 5.26 | 259.0 | 96.9 |
| 59 | 2-keto-3-deoxy-6-phosphogluconate | 5.28 | 257.0 | 97.0 |
| 60 | cyclic guanosine monophosphate | 5.60 | 344.0 | 150.0 |
| 61 | 2-deoxyribose 5-phosphate | 6.45 | 229.0 | 96.8 |
| 62 | glyceraldehyde 3-phosphate | 5.77 | 169.0 | 97.0 |
| 63 | dihydroxyacetone phosphate | 7.60 | 169.0 | 97.0 |
| 64 | sedoheptulose 7-phosphate/arginosuccinat | 5.76 | 289.0 | 97.0 |
| 65 | glycerophosphoric acid | 5.83 | 171.0 | 78.8 |
| 66 | glucose-1-phosphate | 6.11 | 259.0 | 240.8 |
| 67 | nicotinic acid | 6.44 | 122.0 | 77.9 |
| 68 | pantothenic acid | 6.48 | 218.1 | 88.1 |
| 69 | 2-*C*-methylerythritol 4-phosphate | 6.08 | 215.0 | 78.9 |
| 70 | cytidine monophosphate | 6.39 | 322.0 | 79.0 |
| 71 | mevalonic acid lactone | 6.85 | 147.1 | 59.1 |
| 72 | erythrose 4-phosphate | 5.25 | 199.0 | 96.8 |
| 73 | ribulose-5-phosphate/xylulose-5-phosphate | 6.45 | 229.0 | 96.8 |
| 74 | uridine monophosphate | 7.24 | 323.0 | 79.0 |
| 75 | ribose-1-phosphate | 7.25 | 229.0 | 211.0 |
| 76 | 2'-deoxyguanosine 5'-monophosphate | 8.33 | 346.1 | 78.8 |
| 77 | adenosine monophosphate | 8.37 | 346.1 | 78.8 |
| 78 | guanosine 5'-monophosphate | 8.39 | 362.1 | 78.9 |
| 79 | inosinic acid | 8.40 | 347.0 | 134.8 |
| 80 | 1-deoxy-*D*-xylulose 5-phosphate | 8.75 | 213.0 | 97.0 |

| # | Metabolite | $t_R$ (min)[a] | Q1 (*m*/*z*)[б] | Q3 (*m*/*z*)[в] |
|---|---|---|---|---|
| 81 | glutathione disulfide | 8.61 | 611.1 | 306.1 |
| 82 | 2'-deoxyadenosine 5'-monophosphate | 8.83 | 330.1 | 195.0 |
| 83 | digalacturonic acid | 8.77 | 369.1 | 175.0 |
| 84 | thymidine-5'-phosphate | 9.55 | 321.0 | 78.8 |
| 85 | succinic acid | 9.54 | 117.0 | 73.0 |
| 86 | 3-hydroxypyruvate | 9.43 | 103.0 | 59.0 |
| 87 | ureidosuccinic acid | 9.53 | 175.0 | 131.8 |
| 88 | fumaric acid | 9.70 | 115.0 | 71.0 |
| 89 | malate | 9.73 | 133.0 | 115.0 |
| 90 | uridine-5'-diphosphate-glucose | 9.52 | 565.0 | 323.0 |
| 91 | uridine-diphosphate-*N*-acetylglucosamine | 9.60 | 606.1 | 384.8 |
| 92 | sulfate | 9.82 | 97.0 | 97.0 |
| 93 | oxaloacetic acid-1 | 9.90 | 131.0 | 87.0 |
| 94 | oxaloacetic acid-2 | 10.32 | 131.0 | 43.0 |
| 95 | 4-diphosphocytidyl-2-*C*-methyl-*D*-erythritol | 5.57 | 520.1 | 78.9 |
| 96 | adenosine diphosphoribose | 10.26 | 558.1 | 346.0 |
| 97 | adenosine diphosphate glucose | 10.16 | 588.1 | 345.9 |
| 98 | pentanoates | 10.36 | 101.1 | 101.1 |
| 99 | $\alpha$-ketoglutaric acid | 10.33 | 145.0 | 101.0 |
| 100 | 1,4-dihydronicotinamide adenine dinucleotide | 10.80 | 664.1 | 78.9 |
| 101 | (*R*)-5-phosphomevalonic acid | 11.30 | 227.0 | 97.0 |
| 102 | 2*P*-glycolate | 12.82 | 155.0 | 79.0 |
| 103 | xanthosine-5'-phosphate | 12.85 | 363.0 | 151.1 |
| 104 | 2-phosphoglyceric acid | 12.83 | 185.0 | 79.0 |
| 105 | 3-phosphoglyceric acid | 12.76 | 185.0 | 96.7 |
| 106 | (2E)-4-hydroxy-3-methylbut-2-en-1-yl diphosphate | 12.91 | 261.0 | 79.0 |
| 107 | uridine-5'-diphosphate | 13.07 | 403.0 | 78.8 |
| 108 | 2'-deoxyadenosine-5'-diphosphate | 13.20 | 410.0 | 78.9 |
| 109 | isopentenyl pyrophosphate/ | 14.38 | 245.0 | 78.9 |
| | delta3-isopentenyl pyrophosphate | 14.38 | 245.0 | 78.9 |
| 110 | /(dimethylallylpyrophosphat) | 14.22 | 245.0 | 78.9 |
| 111 | flavin adenine dinucleotide | 12.83 | 784.1 | 79.0 |
| 112 | aconitic acid | 13.12 | 173.0 | 128.7 |
| 113 | adenosine-5'-diphosphate | 13.16 | 426.0 | 78.9 |
| 114 | nicotinamide adenine dinucleotide phosphate | 13.12 | 743.1 | 620.0 |
| 115 | guanosine-5'-diphosphate | 13.22 | 442.0 | 78.9 |
| 116 | phosphoenolpyruvic acid | 13.52 | 167.0 | 78.8 |
| 117 | cytidine-5'-diphosphate | 12.77 | 402.0 | 78.9 |
| 118 | thymidine-5'-diphosphate | 14.03 | 401.0 | 78.8 |
| 119 | 6-phosphogluconic acid | 13.01 | 275.0 | 79.0 |
| 120 | trigalacturonic acid | 13.59 | 545.1 | 369.0 |
| 121 | citric acid | 14.05 | 191.0 | 87.0 |
| 122 | isocitric acid | 14.17 | 191.0 | 73.0 |

| # | Metabolite | $t_R$ (min)[a] | Q1 (*m*/*z*)[b] | Q3 (*m*/*z*)[c] |
|---|---|---|---|---|
| 123 | 2-deoxyribose 5-phosphate | 14.57 | 212.9 | 97.1 |
| 124 | orotidine 5'-monophosphate | 15.55 | 367.0 | 78.9 |
| 125 | fructose-1,6-diphosphate | 15.45 | 339.0 | 96.9 |
| 126 | ribulose-1,5-bisphosphate | 15.44 | 309.0 | 97.0 |
| 127 | 4-diphosphocytidyl-2-*C*-methyl-*D*-erythritol 2-phosphate | 15.76 | 600.0 | 78.9 |
| 128 | sedoheptulose-1,7-biphosphate | 15.60 | 369.0 | 97.0 |
| 129 | cytidine 5'-triphosphate | 15.61 | 482.0 | 158.8 |
| 130 | desoxyadenosintriphosphat | 15.83 | 490.0 | 391.9 |
| 131 | mevalonate-5-diphosphate | 15.75 | 307.0 | 78.9 |
| 132 | adenosine triphosphate | 15.90 | 506.0 | 158.8 |
| 133 | guanosine-5'-triphosphate | 15.90 | 522.0 | 158.8 |
| 134 | adenylosuccinic acid | 16.00 | 462.1 | 133.9 |
| 135 | uridine-5'-triphosphate | 15.85 | 483.0 | 158.8 |
| 136 | ADP-ribose-2`-phosphate | 16.40 | 638.0 | 426.0 |
| 137 | dihydronicotinamide adenine dinucleotide phosphate | 16.44 | 744.1 | 79.0 |
| 138 | coenzyme A | 17.32 | 766.1 | 407.9 |
| 139 | inositol triphosphate | 17.30 | 419.0 | 320.8 |
| 140 | deoxythymidine 5'-triphosphate | 15.85 | 481.0 | 158.7 |
| 141 | 5-phosphoribosyl diphosphate | 17.30 | 388.9 | 176.8 |
| 142 | S-acetyl coenzyme A | 17.57 | 808.1 | 407.9 |
| 143 | geranyl diphosphate | 18.33 | 313.1 | 78.9 |
| 144 | methylmalonyl coenzyme A | 18.35 | 866.1 | 408.0 |
| 145 | isovaleryl coenzyme A | 19.20 | 850.2 | 407.9 |
| 146 | acetoacetyl coenzyme A | 19.20 | 850.1 | 408.0 |
| 147 | malonyl coenzyme A | 19.20 | 852.1 | 408.0 |
| 148 | β-hydroxy β-methylglutaryl-CoA | 18.39 | 910.1 | 407.9 |
| 149 | ent-copal-8-ol diphosphate | 18.33 | 866.1 | 407.6 |
| 150 | β-methylcrotonyl coenzyme A | 19.04 | 848.1 | 407.8 |
| 151 | inositol-1,3,4,5-tetraphosphate | 18.80 | 498.3 | 400.7 |
| 152 | 1-diphosinositol pentakisphosphate | 18.91 | 578.9 | 480.6 |
| 153 | geranylgeranyl pyrophosphate | 19.74 | 449.2 | 78.8 |
| 154 | farnesyl diphosphate | 19.70 | 381.1 | 78.9 |
| 155 | phytic acid/inositol hexaphosphate | 19.45 | 658.9 | 560.7 |
| 156 | glucose | 0.79 | 179.1 | 89.0 |
| 157 | 5-amino-4-imidazolecarboxamide ribotide | 8.1 | 337.1 | 78.9 |

[a] The arrangement of the metabolites in the table corresponds to the order of increasing retention times (tR).

[b] m/z of quasi-molecular ions of standard substances

[c] m/z of fragment ion obtained by fragmentation of quasi-molecular ions of standard substances.

**Table S1-6.** Protein extraction yields, protein concentrations in the extracts and optical densities of individual SDS-PAGE lanes corresponding to individual samples of protein extracts isolated from the *Cucumis sativus* roots with overexpression of *CsRALF34* or from roots of control group.

| Sample | Sample weight (g) | Protein concentration (mg/mL) | Protein recovery (mg/g fresh weight) | Optical densities [a] |
|---|---|---|---|---|
| Control (1) | 0.250 | 1.53 | 4.28E-01 | 1595601 |
| Control (2) | 0.254 | 1.13 | 3.12E-01 | 1612235 |
| Control (3) | 0.255 | 1.25 | 3.43E-01 | 1525704 |
| Control (4) | 0.241 | 1.51 | 4.37E-01 | 1525891 |
| Control (5) | 0.236 | 1.35 | 4.00E-01 | 1572125 |
| Control (6) | 0.271 | 1.65 | 4.28E-01 | 1598280 |
| Control (7) | 0.233 | 1.38 | 4.13E-01 | 1625710 |
| Control (8) | 0.284 | 1.88 | 4.63E-01 | 1658598 |
| RALF34+ (1) | 0.253 | 1.47 | 4.07E-01 | 1727770 |
| RALF34+ (2) | 0.248 | 1.64 | 4.62E-01 | 1734730 |
| RALF34+ (3) | 0.273 | 1.50 | 3.85E-01 | 1823112 |
| RALF34+ (4) | 0.224 | 1.05 | 3.30E-01 | 1718919 |
| RALF34+ (5) | 0.284 | 0.95 | 2.34E-01 | 1736292 |
| RALF34+ (6) | 0.244 | 1.33 | 3.82E-01 | 1768621 |
| RALF34+ (7) | 0.260 | 1.51 | 4.05E-01 | 1768116 |
| RALF34+ (8) | 0.224 | 1.27 | 3.97E-01 | 1542560 |

[a] optical densities of individual SDS-PAGE lanes

**Table S1-7.** Eigenvalues (loadings) obtained in the principal component analysis, in particular PC1, of differentially expressed part of *Cucumis sativus* root proteome, associated with *CsRALF34* overexpression.

| Protein name [a] | PC1[b] | Direction of alterations | $log_2FC$[c] | $p_{adjusted}$[d] |
|---|---|---|---|---|
| Thioredoxin | 0.084 | Up | -17.827 | 5.27E-35 |
| CHP-rich zinc finger protein-like | 0.081 | Up | -17.201 | 6.81E-35 |
| Glutathione peroxidase | 0.081 | Up | -17.163 | 4.37E-35 |
| Ribosomal protein L19 | 0.081 | Up | -17.026 | 5.64E-35 |
| 40S ribosomal protein S24 | 0.080 | Up | -16.696 | 3.16E-34 |
| Carnitine operon protein CaiE | 0.079 | Up | -16.568 | 5.64E-35 |
| Wound/stress protein | 0.078 | Up | -16.523 | 4.37E-35 |
| Porin/voltage-dependent anion-selective channel protein | 0.078 | Up | -16.462 | 5.64E-35 |
| 60S ribosomal protein L18a | 0.077 | Up | -16.422 | 6.81E-35 |
| Putative villin 2 protein | 0.075 | Up | -16.340 | 8.87E-35 |
| Eukaryotic translation initiation factor 3 subunit C | 0.077 | Up | -16.305 | 1.36E-32 |
| 3-oxoacyl-[acyl-carrier-protein] synthase 3 | 0.076 | Up | -16.286 | 8.96E-35 |
| Pleckstrin homology domain-containing family A member | 0.077 | Up | -16.272 | 5.00E-16 |
| RNA-binding protein 8A | 0.076 | Up | -16.085 | 8.30E-35 |
| Glycine cleavage system H protein 1 | 0.075 | Up | -16.013 | 8.80E-30 |
| Phosphofructokinase | 0.077 | Up | -15.994 | 8.70E-35 |
| Acidic endochitinase | 0.075 | Up | -15.750 | 2.88E-35 |
| Phosphomannomutase | 0.074 | Up | -15.747 | 9.47E-35 |
| 60S ribosomal protein L35 | 0.074 | Up | -15.745 | 1.51E-29 |
| TBCC domain-containing protein | 0.071 | Up | -15.704 | 9.47E-35 |
| Tripeptidyl peptidase II | 0.073 | Up | -15.642 | 1.02E-34 |
| 40S ribosomal protein S29 | 0.075 | Up | -15.619 | 9.47E-35 |
| 40S ribosomal protein S12 | 0.074 | Up | -15.518 | 8.21E-35 |
| THO complex subunit | 0.073 | Up | -15.495 | 3.51E-28 |
| Ribosomal protein L15 | 0.074 | Up | -15.455 | 1.01E-34 |
| AP-1 complex subunit gamma-2 | 0.072 | Up | -15.414 | 1.17E-34 |
| Putative 3-dehydroquinate synthase | 0.073 | Up | -15.388 | 9.18E-35 |
| 37S ribosomal protein | 0.073 | Up | -15.386 | 8.92E-25 |
| Lipid A export ATP-binding/permease protein MsbA | 0.072 | Up | -15.331 | 3.20E-19 |

| Protein name [a] | PC1[b] | Direction of alterations | $log_2FC$[c] | $p_{adjusted}$[d] |
|---|---|---|---|---|
| Protein phosphatase 2c, putative | 0.072 | Up | -15.319 | 3.61E-34 |
| Transmembrane 9 superfamily protein member | 0.075 | Up | -15.315 | 9.47E-35 |
| Isocitrate dehydrogenase [NADP] | 0.072 | Up | -15.267 | 3.17E-23 |
| Surface antigen (D15) | 0.072 | Up | -15.254 | 1.01E-34 |
| Eukaryotic translation initiation factor 5A | 0.072 | Up | -15.246 | 3.68E-29 |
| Transketolase | 0.073 | Up | -15.228 | 9.47E-35 |
| Histone H2A | 0.072 | Up | -15.194 | 9.47E-35 |
| Phosphoribosylformylglycinamidine cyclo-ligase | 0.073 | Up | -15.188 | 2.09E-34 |
| 5-methyltetrahydropteroyl triglutamate-homocysteine methyltransferase | 0.072 | Up | -15.187 | 2.53E-21 |
| Beta-glucosidase | 0.072 | Up | -15.176 | 1.20E-34 |
| Peroxidase | 0.072 | Up | -15.139 | 1.20E-34 |
| Endo-1,3-1,4-beta-d-glucanase | 0.071 | Up | -15.124 | 9.47E-35 |
| Calmodulin-binding transcription activator | 0.071 | Up | -15.101 | 6.45E-28 |
| NADP-dependent D-sorbitol-6-phosphate dehydrogenase | 0.071 | Up | -15.099 | 6.12E-33 |
| Nonsense-mediated mRNA decay NMD3 family protein | 0.071 | Up | -15.003 | 1.18E-34 |
| UDP-glycosyltransferase 1 | 0.071 | Up | -14.996 | 9.18E-35 |
| Thioredoxin family protein | 0.071 | Up | -14.990 | 5.35E-33 |
| Alpha-galactosidase 1 | 0.070 | Up | -14.943 | 6.75E-34 |
| Acyl-CoA-binding domain-containing protein | 0.073 | Up | -14.933 | 1.08E-33 |
| Vacuolar-sorting receptor 7 | 0.070 | Up | -14.912 | 1.20E-34 |
| ATP-binding cassette | 0.070 | Up | -14.909 | 2.96E-19 |
| Exocyst complex component | 0.070 | Up | -14.897 | 2.65E-23 |
| Adenylosuccinate lyase | 0.070 | Up | -14.896 | 3.33E-28 |
| Acylamino-acid-releasing enzyme | 0.070 | Up | -14.875 | 5.08E-30 |
| Acetolactate synthase small subunit | 0.070 | Up | -14.851 | 1.29E-34 |
| Ran GTPase activating protein 2 | 0.069 | Up | -14.837 | 1.22E-34 |
| Splicing factor 3B subunit | 0.070 | Up | -14.748 | 2.09E-22 |
| 60S ribosomal protein L14 | 0.069 | Up | -14.672 | 1.18E-34 |
| Pro-resilin | 0.069 | Up | -14.666 | 4.77E-29 |
| Methyltransferase | 0.069 | Up | -14.651 | 5.89E-33 |
| Serine/threonine protein kinase | 0.068 | Up | -14.599 | 2.14E-34 |
| Peroxisomal membrane protein 11-1 | 0.068 | Up | -14.586 | 2.43E-34 |
| RuvB-like helicase | 0.071 | Up | -14.568 | 1.64E-34 |
| Calcium dependent protein kinase 12 | 0.069 | Up | -14.524 | 1.43E-34 |
| Signal recognition particle 72 kDa protein | 0.068 | Up | -14.511 | 8.57E-33 |
| Histidine decarboxylase | 0.068 | Up | -14.473 | 1.43E-34 |
| Bifunctional protein folD | 0.068 | Up | -14.411 | 1.46E-34 |
| Nuclear RNA binding protein | 0.068 | Up | -14.381 | 6.59E-21 |
| Allene oxide cyclase 3 | 0.069 | Up | -14.320 | 2.43E-34 |

| Protein name [a] | PC1[b] | Direction of alterations | $log_2FC$[c] | $p_{adjusted}$[d] |
|---|---|---|---|---|
| Bifunctional protein folD | 0.066 | Up | -14.235 | 4.37E-35 |
| Aladin | 0.067 | Up | -14.041 | 2.85E-34 |
| 40S ribosomal protein S25 | 0.066 | Up | -13.980 | 1.47E-34 |
| Serine/threonine protein phosphatase 2A, regulatory subunit | 0.065 | Up | -13.820 | 3.76E-21 |
| Probable exocyst complex component 4 | 0.066 | Up | -13.807 | 2.43E-34 |
| SMP-30/Gluconolaconase/LRE-like region family protein | 0.065 | Up | -13.789 | 1.77E-34 |
| Dihydroorotate dehydrogenase (quinone) | 0.065 | Up | -13.779 | 1.46E-25 |
| Glutamine-fructose-6-phosphate aminotransferase [isomerizing] 2 | 0.065 | Up | -13.730 | 1.72E-19 |
| Phosphomevalonate kinase | 0.064 | Up | -13.618 | 4.98E-33 |
| Protein transport protein SEC23 | 0.064 | Up | -13.612 | 9.65E-34 |
| Small nuclear ribonucleoprotein-associated protein | 0.063 | Up | -13.434 | 6.54E-27 |
| Mannan synthase | 0.063 | Up | -13.399 | 4.94E-34 |
| General vesicular transport factor p115 | 0.062 | Up | -13.129 | 4.11E-34 |
| Glucosidase II beta subunit | 0.062 | Up | -13.041 | 2.27E-19 |
| ATP-dependent RNA helicase | 0.061 | Up | -12.961 | 1.05E-19 |
| Early-responsive to dehydration | 0.061 | Up | -12.920 | 2.23E-16 |
| LETM1 and EF-hand domain-containing protein 1 | 0.062 | Up | -12.768 | 7.18E-35 |
| RNA recognition motif-containing protein | 0.017 | Up | -4.239 | 4.50E-07 |
| Brefeldin A-inhibited guanine nucleotide-exchange protein | 0.009 | Up | -2.074 | 2.03E-02 |
| Chaperone protein htpG family protein | 0.009 | Up | -2.010 | 2.69E-02 |
| Translational activator GCN1 | 0.008 | Up | -1.933 | 2.69E-02 |
| ADP-ribosylation factor family protein | 0.009 | Up | -1.874 | 5.45E-04 |
| Ras protein Rab-2-B | 0.008 | Up | -1.872 | 1.23E-04 |
| 3.1.2 inositol monophosphatase | 0.007 | Up | -1.861 | 3.17E-04 |
| UDP-glycosyltransferase 1 | -0.008 | Down | 1.611 | 1.62E-03 |
| Subtilisin-like serine protease | -0.008 | Down | 1.790 | 9.16E-04 |
| 2-oxoglutarate-dependent dioxygenase | -0.009 | Down | 1.956 | 4.49E-02 |
| Transitional endoplasmic reticulum ATPase | -0.010 | Down | 2.072 | 2.96E-02 |
| Myosin-6, (Protein of unknown function DUF827, plant) | -0.013 | Down | 3.669 | 5.22E-03 |
| Phosphorylase | -0.057 | Down | 12.172 | 2.81E-24 |
| Serine/threonine protein phosphatase 2A 57 kDa regulatory subunit B' beta | -0.058 | Down | 12.279 | 2.27E-25 |
| UDP-glycosyltransferase 1 | -0.059 | Down | 12.507 | 8.02E-34 |
| Proteasome component ECM29 | -0.061 | Down | 12.859 | 3.88E-34 |
| Peroxidase | -0.062 | Down | 12.863 | 4.60E-34 |
| Tetratricopeptide repeat protein | -0.062 | Down | 12.864 | 8.49E-34 |
| ADP-ribosylation factor family protein | -0.061 | Down | 12.928 | 1.08E-32 |
| Kinesin-4 | -0.061 | Down | 13.012 | 8.22E-26 |

| Protein name [a] | PC1[b] | Direction of alterations | $log_2FC$[c] | $p_{adjusted}$[d] |
|---|---|---|---|---|
| Hexokinase 1 | -0.061 | Down | 13.057 | 1.16E-33 |
| Armadillo/beta-catenin repeat family protein | -0.062 | Down | 13.133 | 1.63E-19 |
| Oxidoreductase nad-binding rossmann fold protein | -0.064 | Down | 13.388 | 5.07E-34 |
| Phosphatidylinositol 4-kinase | -0.064 | Down | 13.413 | 4.11E-34 |
| AT5g06970/MOJ9_14 | -0.064 | Down | 13.576 | 6.93E-35 |
| Cytochrome P450 | -0.064 | Down | 13.613 | 4.35E-29 |
| Malic enzyme | -0.066 | Down | 13.615 | 1.82E-34 |
| UDP-glycosyltransferase 74 F1 | -0.064 | Down | 13.651 | 1.45E-34 |
| Cystathionine gamma synthase | -0.065 | Down | 13.701 | 3.19E-34 |
| AP-2 complex subunit beta-1 | -0.065 | Down | 13.704 | 5.74E-34 |
| Leukotriene A4 hydrolase/aminopeptidase | -0.067 | Down | 13.753 | 4.79E-35 |
| Ubiquitin carboxyl-terminal hydrolase | -0.065 | Down | 13.793 | 4.11E-34 |
| Inositol-tetrakisphosphate 1-kinase | -0.066 | Down | 13.991 | 4.60E-34 |
| 3,4-dihydroxy 2-butanone 4-phosphate synthase | -0.066 | Down | 13.993 | 3.03E-32 |
| Thioredoxin domain-containing protein | -0.066 | Down | 14.081 | 2.85E-33 |
| Beta-galactosidase | -0.068 | Down | 14.123 | 1.36E-34 |
| Peroxidase (Haem peroxidase) | -0.068 | Down | 14.135 | 2.07E-34 |
| Dynamin | -0.067 | Down | 14.232 | 4.37E-35 |
| NADPH:quinone oxidoreductase | -0.069 | Down | 14.235 | 1.22E-34 |
| Auxin F-box protein 5 | -0.067 | Down | 14.261 | 1.89E-34 |
| Lipoxygenase | -0.069 | Down | 14.299 | 1.77E-34 |
| Phytochrome | -0.067 | Down | 14.312 | 1.16E-34 |
| ATRAD3 (Putative S-adenosyl-L-methionine-dependent methyltransferase) | -0.066 | Down | 14.327 | 1.82E-34 |
| Pentatricopeptide repeat-containing protein | -0.068 | Down | 14.342 | 2.10E-34 |
| Signal recognition particle 19 kDa protein | -0.068 | Down | 14.389 | 3.64E-32 |
| Argininosuccinate lyase | -0.068 | Down | 14.390 | 6.79E-28 |
| AMP deaminase | -0.068 | Down | 14.398 | 4.84E-24 |
| Acetyl-coenzyme A synthetase | -0.068 | Down | 14.410 | 2.35E-25 |
| 3,4-dihydroxy 2-butanone 4-phosphate synthase | -0.068 | Down | 14.478 | 4.09E-28 |
| Sieve element occlusion protein 2 | -0.070 | Down | 14.556 | 5.15E-34 |
| Uridine kinase | -0.069 | Down | 14.690 | 4.37E-35 |
| RNA-dependent RNA polymerase 1b | -0.069 | Down | 14.739 | 2.89E-31 |
| Prolyl 4-hydroxylase alpha subunit | -0.070 | Down | 14.753 | 1.79E-25 |
| 50S ribosomal protein L1 | -0.069 | Down | 14.753 | 1.36E-34 |
| Lipoxygenase | -0.070 | Down | 14.756 | 5.15E-26 |
| ATP phosphoribosyltransferase | -0.070 | Down | 14.780 | 1.42E-34 |
| 1-aminocyclopropane-1-carboxylate oxidase | -0.070 | Down | 14.782 | 8.73E-22 |
| GDSL esterase/lipase | -0.070 | Down | 14.804 | 3.98E-34 |
| 5-oxoprolinase | -0.071 | Down | 14.809 | 1.22E-34 |
| NADPH-cytochrome P450 reductase | -0.070 | Down | 14.837 | 6.56E-23 |

| Protein name [a] | PC1[b] | Direction of alterations | $log_2FC$[c] | $p_{adjusted}$[d] |
|---|---|---|---|---|
| Beta-glucosidase | -0.070 | Down | 14.842 | 9.47E-35 |
| Ankyrin repeat-containing protein | -0.070 | Down | 14.843 | 2.95E-25 |
| Ubiquitin-protein ligase 4; contains IPR000569 (HECT), IPR016024 (Armadillo-type fold) | -0.068 | Down | 14.894 | 1.20E-34 |
| Acyl-CoA dehydrogenase | -0.071 | Down | 14.894 | 1.20E-34 |
| NADP-dependent D-sorbitol-6-phosphate dehydrogenase | -0.070 | Down | 14.933 | 2.06E-22 |
| Sugar transporter | -0.071 | Down | 14.958 | 1.29E-34 |
| Monoglyceride lipase | -0.070 | Down | 14.964 | 9.47E-35 |
| Translin | -0.073 | Down | 14.993 | 2.25E-34 |
| Malic enzyme | -0.071 | Down | 15.044 | 1.20E-34 |
| Signal peptidase complex subunit 2 | -0,072 | Down | 15.070 | 1.53E-34 |
| Major facilitator superfamily domain-containing protein | -0.070 | Down | 15.083 | 1.23E-33 |
| UDP-glycosyltransferase 1 | -0.071 | Down | 15.095 | 8.51E-35 |
| Deoxyuridine 5'-triphosphatenucleotido hydrolase | -0.073 | Down | 15.102 | 4.65E-34 |
| Endo-1,4-beta-glucanase | -0.070 | Down | 15.134 | 8.48E-35 |
| Cullin-1 | -0.071 | Down | 15.155 | 9.47E-35 |
| Cyclin dependent kinase A | -0.071 | Down | 15.157 | 4.06E-25 |
| Putative vesicle-associated membrane protein family protein | -0.072 | Down | 15.172 | 1.20E-34 |
| Ferredoxin | -0.072 | Down | 15.182 | 1.16E-34 |
| Protein kinase | -0.071 | Down | 15.198 | 9.47E-35 |
| 1-pyrroline-5-carboxylate dehydrogenase | -0.072 | Down | 15.273 | 9.89E-35 |
| Heat shock 70 kDa protein | -0.072 | Down | 15.274 | 9.47E-35 |
| Catalase | -0.073 | Down | 15.317 | 1.26E-34 |
| Coatomer subunit zeta-1 | -0.074 | Down | 15.318 | 8.70E-35 |
| Nitrogen regulatory protein P-II | -0.072 | Down | 15.337 | 7.83E-35 |
| Pre-mRNA-processing factor-like protein | -0.073 | Down | 15.400 | 5.10E-27 |
| Small nuclear ribonucleoprotein E | -0.073 | Down | 15.413 | 1.06E-34 |
| Membrane protein insertase YidC | -0.072 | Down | 15.444 | 1.07E-34 |
| Dolichyl-diphosphooligosaccharide-protein glycosyltransferase subunit | -0.073 | Down | 15.445 | 5.96E-30 |
| Vacuolar protein sorting-associated | -0.073 | Down | 15.569 | 6.81E-35 |
| Costars family protein ABRACL | -0.073 | Down | 15.585 | 3.90E-29 |
| Putative 4-methyl-5(B-hydroxyethyl)-thiazol monophosphate biosynthesis enzyme | -0.073 | Down | 15.590 | 7.18E-35 |
| Aquaporin | -0.074 | Down | 15.594 | 9.47E-35 |
| 60S ribosomal protein L38e | -0.075 | Down | 15.668 | 8.87E-35 |
| Patatin-like protein | -0.074 | Down | 15.720 | 2.53E-34 |
| Splicing factor U2AF subunit | -0.075 | Down | 15.745 | 9.18E-35 |

| Protein name [a] | PC1[b] | Direction of alterations | $log_2FC$[c] | $p_{adjusted}$[d] |
|---|---|---|---|---|
| Eukaryotic translation initiation factor 3 subunit B | -0.074 | Down | 15.763 | 9.47E-35 |
| Reticulon family protein | -0.074 | Down | 15.766 | 9.47E-35 |
| Lysine/histidine transporter | -0.075 | Down | 15.767 | 9.18E-35 |
| Mitochondrial uncoupling protein | -0.077 | Down | 15.850 | 8.87E-35 |
| Vacuolar protein sorting-associated protein 27 | -0.075 | Down | 15.927 | 9.47E-35 |
| Glutathione S-transferase | -0.077 | Down | 15.949 | 9.12E-35 |
| Glycylpeptide N-tetradecanoyltransferase | -0.075 | Down | 15.960 | 8.54E-29 |
| Developmentally-regulated GTP-binding protein 2 | -0.076 | Down | 16.013 | 3.89E-33 |
| Histidinol-phosphate aminotransferase | -0.074 | Down | 16.017 | 9.47E-35 |
| Clp protease ATP binding subunit | -0.076 | Down | 16.169 | 8.65E-35 |
| Adenylate kinase | -0.076 | Down | 16.193 | 3.38E-30 |
| Progesterone 5-beta-reductase | -0.076 | Down | 16.200 | 1.23E-34 |
| Xylose isomerase | -0.077 | Down | 16.342 | 7.74E-35 |
| Succinyl-CoA ligase [ADP-forming] subunit beta | -0.078 | Down | 16.504 | 7.18E-35 |
| Splicing factor U2af 38 kDa subunit | -0.078 | Down | 16.556 | 1.56E-32 |
| Proliferating cell nuclear antigen | -0.078 | Down | 16.569 | 1.90E-28 |
| Translocon-associated protein | -0.078 | Down | 16.685 | 6.03E-35 |
| NADH dehydrogenase 1 alpha subcomplex subunit 13 | -0.079 | Down | 16.852 | 5.64E-35 |
| Arginine/serine-rich splicing factor | -0.078 | Down | 16,986 | 4.37E-35 |
| Protein MEMO1 | -0.081 | Down | 17.238 | 6.81E-35 |
| 60S ribosomal protein L32 | -0.082 | Down | 17.330 | 1.99E-30 |
| Subtilisin-like serine protease | -0.081 | Down | 17.420 | 8.30E-35 |
| Pentatricopeptide repeat-containing protein | -0.082 | Down | 17.517 | 1.70E-35 |
| 10 kDa chaperonin | -0.083 | Down | 17.580 | 4.79E-35 |
| AP-4 complex accessory subunit tepsin | -0.083 | Down | 17.647 | 2.41E-34 |
| Phloem lectin | -0.086 | Down | 18.212 | 4.79E-35 |
| Ubiquitin-like domain-containing CTD phosphatase | -0.089 | Down | 18.696 | 4.49E-35 |
| 70 kDa heat shock protein | -0.100 | Down | 21.144 | 1.70E-35 |

[a]Identification of peptides and annotation of protein relied on a search against amino acid sequences of *Cucumis sativus* cv. Chinese Long v2 (obtained from the FTP-site of the Cucurbit Genomics Database v1) accomplished with SEQUEST algorithm, Mercator4 (v.2.0) was used for proteins annotation, subcellular localization of proteins was defined using WoLF PSORT. Data are available via ProteomeXchange with identifier PXD037725; [b]PC, ptincipal component; [c]FC, fold change; [d]$p_{adjusted}$, adjusted p value.

**Table S1-8.** Gas chromatographic (GC) separation conditions and electron ionization-quadrupole-mass spectrometry (EI-Q-MS) settings for analysis of primary thermally stabile metabolites from *Cucumis sativus* roots with GC-EI-Q-MS.

| **Parameters** | **Settings** |
|---|---|
| | **GC settings** |
| Separation column | MEGA-5 MS capillary column (30 m × 0.32 mm ID, 0.25 µm film thickness, MEGA S.r.l., Legnano, Italy) |
| Carrier gas / carrier gas flow rate | Helium/1.5 mL/min |
| Injector operation mode | Splitless mode (90 s splitless time) |
| Injector temperature | 280°C |
| Temperature program | 1 min at 40°C<br>ramp 15°C/min to 70°C<br>1 min at 70°C<br>ramp 6°C/min to 320°C<br>12 min at 320°C |
| **Parameters** | **MS settings** |
| Ionization mode | Electron ionization (EI) |
| Electron energy | 70 eV |
| Operation mode | Positive, scanning at 0.34 sec $scan^{-1}$ |
| *m*/*z* range | 50 - 700 |

The analysis relied on Shimadzu GCMS-QP2010 Ultra, equipped with an auto sampler AOC-5000 Plus (Shimadzu).

**Table S1-9.** The conditions of ion pair-reversed phase ultrahigh performance liquid chromatographic (IP-RP-UHPLC) separation and the settings for electrospray ionization-triple quadrupole-tandem mass spectrometry (ESI-QqQ-MS/MS) used for the analysis of anionic primary thermally labile metabolites from *Cucumis sativus* root with IP-RP-UHPLC- ESI-QqQ-MS/MS.

**Chromatography**

| **ACQUITY Sample Manager (SM)** | |
|---|---|
| Injection mode | PartialLoop |
| Injection volume | 5 µL |
| Weak wash solvent | 0.3 mmol/L aq. ammonium formate |
| Weak wash volume | 800 µL |
| Strong wash solvent | Acetonitrile |
| Strong wash volume | 400 µL |
| Target sample temperature | 4.0 C |
| Needle overfill flush | Automatic |

| Column conditions | |
|---|---|
| Separation column | EC 150/2 NUCLEOSHELL RP 18<br>(150 x 2 mm, particle size 2.7 µm) |
| Target column temperature | 40.0 C |

| ACQUITY Binary Solvent Manager (BSM) | |
|---|---|
| Eluent A | 0.3 mmol/L aq. ammonium formate |
| Eluent B | Acetonitrile |
| Seal wash duration | 5 min |
| Flow rate | 0.4 mL/min |
| Elution program | 2% eluent B isocratic - 2 min<br>gradient to 36% eluent B – 16 min |

**Mass spectrometry**

| General | |
|---|---|
| Mass analyzer type | triple quadrupole-linear ion trap (QqLIT, QTRAP, operated in QqQ mode) |
| Ion source | TurboIonSpray® |
| Experiment type | multiple reaction monitoring (MRM) |
| Operatinon mode | negative |
| Cycle time (s) | 1.1 |
| Pause between ranges (ms) | 5.007 |
| Settling time (s) | 0 |
| Duration | 24 min |

| Ion source settings | |
|---|---|
| Nebulizer gas (psig) | 60 |
| Drying gas (psig) | 70 |
| Curtain gas (psig) | 40 |
| Ion spray voltage (kV) | -4.5 |
| Ion source temperature (°C) | 450 |

| MS/MS settings | |
|---|---|
| Fragmentation mode | CAD |
| MS/MS experiment type | MRM |
| Collision gas | nitrogen |
| Collision gas pressure | 3 psig (medium) |

| | |
|---|---|
| Entrance potential (V) | -10.0 |
| Scheduled MRM | enabled |
| Scheduled MRM type | basic |
| MRM detection window (s) | 500 |
| Target scan time (s) | 1 |
| Dwell time | adjusted by scheduled MRM algorithm |
| Q1 resolution | unit |
| Q3 resolution | unit |
| Declustering potential (DP, V) | compound-specific (listed below) |
| Collision potential (CE, V) | compound-specific (listed below) |
| Exit potential (CXP, V) | compound-specific (listed below) |

**Analyte-specific settings**

**Analyte-specific combinations of Q1 and Q3 *m/z* ranges (transitions)**

| # | Analyte | $t_R$ (min) | Q1 (*m/z*) | Q3 (*m/z*) | DP (V) | CE (V) | CXP (V) |
|---|---|---|---|---|---|---|---|
| 1 | 2-deoxy-*D*-ribose 5-phosphate | N/A | 212.9 | 97.1 | -40 | -20 | -19 |
| 2 | 3-[(carboxylatovinyl)oxy]benzoate | N/A | 207.1 | 179.0 | -240 | -38 | -13 |
| 3 | 5-amino-4-imidazolecarboxamide ribotide | N/A | 337.1 | 78.9 | -85 | -50 | -5 |
| 4 | 5-formamido-1-(5-phospho-D-ribosyl)imidazole-4-carboxamide | N/A | 365.0 | 78.9 | -40 | -35 | -10 |
| 5 | 5-formyl-tetrahydrofolate | N/A | 472.2 | 315.1 | -40 | -35 | -10 |
| 6 | 5-methyl-tetrahydrofolate | N/A | 458.2 | 329.1 | -40 | -35 | -10 |
| 7 | 5′-phosphoribosyl-*N*-formylglycinamide | N/A | 313.0 | 78.9 | -40 | -35 | -10 |
| 8 | 5'-phosphoribosyl-5-aminoimidazole | N/A | 294.0 | 78.9 | -40 | -43 | -10 |
| 9 | allantoic acid | N/A | 175.0 | 132.0 | -35 | -32 | -12 |
| 10 | beta-nicotinamide mononucleotide | N/A | 334.0 | 78.9 | -25 | -16 | -13 |
| 11 | carboxyaminoimidazole ribotide | N/A | 338.0 | 78.9 | -40 | -35 | -10 |
| 12 | chorismate | N/A | 225.0 | 179.0 | -35 | -25 | -10 |
| 13 | cytidine-5'-diphosphate choline | N/A | 487.0 | 428.0 | -10 | -20 | -23 |
| 14 | glycineamide ribonucleotide | N/A | 285.0 | 78.9 | -40 | -35 | -10 |
| 15 | nicotinamide | N/A | 121.0 | 76.9 | -40 | -16 | -9 |
| 16 | nicotinamide mononucleotide | N/A | 333.0 | 78.9 | -50 | -30 | -13 |
| 17 | nicotinamide riboside | N/A | 253.1 | 121.0 | -40 | -35 | -10 |
| 18 | phosphoribosylamine | N/A | 227.0 | 78.9 | -40 | -35 | -10 |
| 19 | riboflavin-5′-phosphate | N/A | 455.1 | 97.0 | -35 | -25 | -10 |
| 20 | succinylaminoimidazole-carboxamide ribotide | N/A | 453.1 | 78.9 | -40 | -35 | -10 |
| 21 | tetrahydrofolate | N/A | 444.2 | 176.1 | -40 | -35 | -10 |

| | | | | | | | |
|---|---|---|---|---|---|---|---|
| 22 | histidine | 0.5 | 154.1 | 93.0 | -40 | -24 | -3 |
| 23 | arginine | 0.6 | 173.1 | 131.0 | -50 | -18 | -7 |
| 24 | glutamine | 0.6 | 145.1 | 108.9 | -30 | -18 | -5 |
| 25 | ornithine | 0.6 | 131.1 | 82.9 | -60 | -20 | -5 |
| 26 | proline | 0.6 | 114.1 | 86.0 | -55 | -18 | -3 |
| 27 | 4-aminobutanoic acid | 0.7 | 102.1 | 84.0 | -35 | -14 | -7 |
| 28 | alanine | 0.7 | 88.0 | 41.9 | -20 | -20 | -13 |
| 29 | allantoin | 0.7 | 157.0 | 97.2 | -60 | -16 | -1 |
| 30 | asparagine | 0.7 | 131.1 | 87.1 | -75 | -16 | -11 |
| 31 | citrulline | 0.7 | 174.1 | 131.0 | -35 | -18 | -7 |
| 32 | creatine | 0.7 | 130.1 | 88.1 | -25 | -14 | -5 |
| 33 | cysteine | 0.7 | 120.0 | 79.8 | -25 | -32 | -2 |
| 34 | lysine | 0.7 | 145.1 | 99.0 | -65 | -14 | -5 |
| 35 | cystine | 0.8 | 239.3 | 120.0 | -40 | -32 | -1 |
| 36 | dehydroascorbic acid | 0.8 | 173.0 | 127.0 | -15 | -18 | -17 |
| 37 | glycine | 0.8 | 74.0 | 74.0 | -36 | -13 | -3 |
| 38 | methionine | 0.8 | 148.0 | 47.0 | -45 | -24 | -5 |
| 39 | hexoses | 0.8 | 179.1 | 89.0 | -50 | -12 | -13 |
| 40 | S-adenosyl-*L*-homocysteine | 0.8 | 383.1 | 133.9 | -80 | -36 | -7 |
| 41 | serine | 0.8 | 104.0 | 74.0 | -20 | -16 | -3 |
| 42 | sucrose | 0.8 | 341.1 | 89.0 | -240 | -38 | -13 |
| 43 | threonine | 0.8 | 118.1 | 73.9 | -25 | -18 | -3 |
| 44 | uridine | 0.8 | 243.1 | 109.9 | -65 | -22 | -5 |
| 45 | valine | 0.8 | 233.3 | 116.0 | -25 | -10 | -5 |
| 46 | valine | 0.8 | 116.1 | 7.0 | -25 | -20 | -5 |
| 47 | cytidine | 1.0 | 242.1 | 108.9 | -70 | -18 | -5 |
| 48 | leucine + isoleucine | 1.0 | 261.3 | 130.2 | -30 | -10 | -1 |
| 49 | leucine + isoleucine | 1.0 | 130.1 | 87.1 | -25 | -25 | -10 |
| 50 | tyrosine | 1.0 | 180.1 | 118.9 | -60 | -24 | -5 |
| 51 | guanosine | 1.4 | 282.1 | 149.9 | -80 | -26 | -7 |
| 52 | 2'-deoxyguanosine | 1.5 | 266.1 | 150.0 | -115 | -24 | -3 |
| 53 | adenosine | 1.5 | 266.1 | 133.9 | -70 | -12 | -1 |
| 54 | phenylalanine | 1.5 | 164.1 | 103.0 | -55 | -24 | -5 |
| 55 | aspartic acid | 1.9 | 132.0 | 88.0 | -40 | -18 | -13 |
| 56 | glucopyranonic acid | 1.9 | 193.0 | 113.0 | -20 | -16 | -5 |
| 57 | galactopyranuronic acid | 2.0 | 193.0 | 113.0 | -20 | -16 | -5 |
| 58 | glyoxilic acid | 2.0 | 73.0 | 45.0 | -25 | -25 | -10 |
| 59 | *D*-galactonic acid/ *D*-gluconic acid | 2.1 | 195.1 | 129.0 | -50 | -18 | -9 |
| 60 | glucosamine 6-phosphate | 2.1 | 258.0 | 97.0 | -45 | -24 | -5 |
| 61 | glutamic acid | 2.1 | 146.0 | 102.0 | -80 | -18 | -9 |
| 62 | ribonic acid | 2.3 | 165.0 | 75.0 | -45 | -20 | -35 |
| 63 | glucosamine 1-phosphate | 2.4 | 258.0 | 78.9 | -55 | -42 | -1 |
| 64 | 2′-deoxyadenosine | 2.5 | 250.1 | 134.0 | -115 | -26 | -9 |
| 65 | glucolate | 2.5 | 75.0 | 47.0 | -30 | -14 | -13 |
| 66 | shikimic acid | 2.5 | 173.0 | 92.9 | -15 | -20 | -5 |
| 67 | 3-dehydroxyshikimic acid | 2.7 | 171.0 | 127.0 | -25 | -16 | -15 |
| 68 | quinic acid | 2.7 | 191.1 | 85.0 | -50 | -28 | -13 |
| 69 | uric acid | 2.7 | 167.0 | 124.0 | -45 | -20 | -7 |
| 70 | ascorbic acid | 2.8 | 175.0 | 115.0 | -25 | -25 | -5 |
| 71 | carbamoyl-alanine | 2.8 | 131.0 | 87.9 | -10 | -14 | -13 |
| 72 | chloride | 2.8 | 35.0 | 35.0 | -50 | -10 | -10 |
| 73 | dihydroorotic acid | 3.3 | 157.0 | 112.7 | -40 | -10 | -5 |

| | | | | | | | |
|---|---|---|---|---|---|---|---|
| 74 | tryptophan | 3.4 | 203.1 | 116.2 | -50 | -22 | -7 |
| 75 | lactic acid | 3.8 | 89.0 | 42.9 | -15 | -12 | -5 |
| 76 | glutathione | 4.0 | 306.1 | 143.0 | -5 | -26 | -7 |
| 77 | phosphate | 4.0 | 96.9 | 78.9 | -40 | -18 | -15 |
| 78 | cyclic guanosine monophosphate | 4.5 | 344.0 | 150.0 | -70 | -34 | -11 |
| 79 | orotic acid | 4.8 | 155.0 | 110.7 | -25 | -12 | -5 |
| 80 | pyruvic acid | 4.9 | 87.0 | 43.0 | -30 | -12 | -1 |
| 81 | nicotinamide adenine dinucleotide | 5.0 | 662.1 | 540.1 | -45 | -22 | -15 |
| 82 | glucose 6-phosphate | 5.4 | 259.1 | 97.0 | -65 | -18 | -13 |
| 83 | glyceraldehyde 3-phosphate | 5.4 | 169.0 | 97.0 | -30 | -12 | -5 |
| 84 | fructose 6-phosphate | 5.5 | 259.0 | 96.9 | -30 | -20 | -11 |
| 85 | 2-keto-3-deoxy-6-phosphogluconate | 5.8 | 257.0 | 97.0 | -30 | -20 | -9 |
| 86 | erythrose 4-phosphate | 5.9 | 199.0 | 96.8 | -40 | -12 | -5 |
| 87 | adenosine 2',3'-cyclic mono-phosphate | 6.2 | 328.0 | 134.0 | -125 | -36 | -5 |
| 88 | ribulose-5-phosphate | 6.3 | 229.0 | 96.8 | -35 | -20 | -5 |
| 89 | glucose-1-phosphate | 6.4 | 259.0 | 240.8 | -30 | -16 | -15 |
| 90 | ribulose-5-phosphate/xylulose-5-phosphate | 6.5 | 229.0 | 96.8 | -45 | -18 | -15 |
| 91 | mevalonic acid lactone | 6.6 | 147.1 | 59.1 | -45 | -20 | -7 |
| 92 | sedoheptulose 7-phosphate | 6.6 | 289.0 | 97.0 | -50 | -22 | -5 |
| 93 | 2-C-methylerythritol 4-phosphate | 6.8 | 215.0 | 78.9 | -40 | -56 | -9 |
| 94 | glycerophosphoric acid | 6.8 | 171.0 | 78.8 | -45 | -24 | -1 |
| 95 | cytidine monophosphate | 7.0 | 322.2 | 79.0 | -65 | -68 | -5 |
| 96 | nicotinic acid | 7.0 | 122.0 | 77.9 | -55 | -16 | -13 |
| 97 | pantothenic acid | 7.0 | 218.1 | 88.1 | -55 | -18 | -5 |
| 98 | adenosine 3',5'-cyclic mono-phosphate | 7.2 | 328.0 | 134.0 | -125 | -36 | -5 |
| 99 | ribose-1-phosphate | 7.7 | 229.0 | 211.0 | -50 | -14 | -3 |
| 100 | uridine monophosphate | 7.8 | 323.0 | 79.0 | -65 | -68 | -5 |
| 101 | guanosine 5'-monophosphate | 8.1 | 362.1 | 78.9 | -65 | -66 | -5 |
| 102 | inosinic acid | 8.3 | 347.0 | 134.8 | -70 | -38 | -7 |
| 103 | 2'-deoxyguanosine 5'-monophosphate | 8.4 | 346.1 | 78.8 | -80 | -42 | -3 |
| 104 | dihydroxyacetone phosphate | 8.5 | 169.1 | 97.0 | -35 | -14 | -11 |
| 105 | thymidine-5'-phosphate | 8.7 | 321.0 | 78.8 | -65 | -58 | -3 |
| 106 | 1-deoxy-D-xylulose 5-phosphate | 8.8 | 213.0 | 97.0 | -50 | -18 | -1 |
| 107 | adenosine monophosphate | 8.8 | 346.1 | 78.8 | -70 | -52 | -3 |
| 108 | glutathione disulfide | 8.9 | 611.1 | 306.1 | -35 | -34 | -7 |
| 109 | 2'-deoxyadenosine 5'-monophosphate | 9.1 | 330.1 | 195.0 | -85 | -22 | -17 |
| 110 | digalacturonic acid | 9.5 | 369.1 | 175.0 | -75 | -18 | -17 |
| 111 | phosphocreatine | 9.6 | 210.0 | 78.9 | -35 | -22 | -1 |
| 112 | malate | 9.8 | 133.0 | 115.0 | -20 | -16 | -5 |
| 113 | succinic acid | 9.9 | 117.0 | 73.0 | -25 | -16 | -7 |
| 114 | 3-hydroxypyruvate | 10.0 | 103.0 | 59.0 | -30 | -22 | -7 |
| 115 | 4-diphosphocytidyl-2-C-methyl-*D*-erythritol | 10.0 | 520.1 | 78.9 | -120 | -108 | -9 |
| 116 | ureidosuccinic acid | 10.1 | 175.1 | 131.8 | -25 | -16 | -7 |
| 117 | sulfate | 10.2 | 97.0 | 97.0 | -40 | -18 | -15 |
| 118 | uridine-5'-diphosphate-glucose | 10.2 | 565.0 | 323.0 | -125 | -36 | -11 |
| 119 | uridine-diphosphate-*N*-acetylglucosamine | 10.2 | 606.1 | 384.8 | -175 | -36 | -25 |
| 120 | fumaric acid | 10.4 | 115.0 | 71.0 | -5 | -12 | -13 |
| 121 | adenosine diphosphoribose | 10.5 | 558.1 | 346.0 | -170 | -34 | -19 |
| 122 | oxaloacetic acid-1 | 10.5 | 131.0 | 87.0 | -35 | -10 | -17 |

| | | | | | | | |
|---|---|---|---|---|---|---|---|
| 123 | oxaloacetic acid-2 | 10.5 | 131.0 | 43.0 | -35 | -18 | -11 |
| 124 | adenosine diphosphate glucose | 10.6 | 588.1 | 345.9 | -140 | -32 | -19 |
| 125 | $\alpha$-ketoglutaric acid | 10.7 | 145.0 | 101 | -10 | -12 | -13 |
| 126 | pentanoates | 10.7 | 101.1 | 101.1 | -50 | -10 | -5 |
| 127 | 2C-methyl-*D*-erythritol 2,4-cyclodiphosphate | 10.9 | 277.0 | 79.0 | -45 | -64 | -37 |
| 128 | (R)-5-phosphomevalonic acid | 11.2 | 227.0 | 97.0 | -30 | -35 | -10 |
| 129 | 1,4-dihydronicotinamide adenine dinucleotide | 11.4 | 664.1 | 78.9 | -100 | -124 | -1 |
| 130 | folate | 11.5 | 440.1 | 311.1 | -40 | -35 | -10 |
| 131 | 2-phosphoglyceric acid | 12.7 | 185.0 | 79.0 | -25 | -20 | -35 |
| 132 | isopentenyl pyrophosphate | 12.8 | 245.0 | 78.9 | -15 | -44 | -37 |
| 133 | 2*P*-glycolate | 12.9 | 155.0 | 79.0 | -15 | -36 | -35 |
| 134 | xanthosine-5'-phosphate | 12.9 | 363.0 | 151.1 | -60 | -36 | -5 |
| 135 | guanosine-5'-diphosphate | 13.0 | 442.0 | 78.9 | -85 | -70 | -3 |
| 136 | 6-phosphogluconic acid | 13.1 | 275.0 | 79.0 | -60 | -66 | -5 |
| 137 | flavin adenine dinucleotide | 13.1 | 784.1 | 79.0 | -60 | -130 | -1 |
| 138 | uridine-5'-diphosphate | 13.1 | 403.0 | 78.8 | -75 | -68 | -3 |
| 139 | cytidine-5'-diphosphate | 13.1 | 402.0 | 78.9 | -65 | -70 | -5 |
| 140 | 3-phosphoglyceric acid | 13.2 | 185.0 | 96.7 | -30 | -22 | -7 |
| 141 | (2E)-4-hydroxy-3-methylbut-2-en-1-yl diphosphate | 13.3 | 261.0 | 79.0 | -40 | -52 | -9 |
| 142 | aconitic acid | 13.3 | 173.0 | 128.7 | -25 | -10 | -55 |
| 143 | adenosine-5'-diphosphate | 13.3 | 426.2 | 78.9 | -75 | -66 | -3 |
| 144 | nicotinamide adenine dinucleotide phosphate | 13.4 | 743.1 | 620.0 | -55 | -22 | -17 |
| 145 | thymidine-5'-diphosphate | 13.4 | 401.0 | 78.8 | -70 | -68 | -3 |
| 146 | 2'-deoxyadenosine-5'-diphosphate | 13.5 | 410.0 | 78.9 | -60 | -76 | -3 |
| 147 | trigalacturonic acid | 13.8 | 545.1 | 369.0 | -105 | -24 | -25 |
| 148 | phosphoenolpyruvic acid | 13.9 | 167.0 | 78.8 | -20 | -16 | -9 |
| 149 | isocitric acid | 14.1 | 191.1 | 73.0 | -45 | -28 | -31 |
| 150 | citric acid | 14.2 | 191.0 | 87.0 | -35 | -22 | -15 |
| 151 | dimethylallylpyrophosphat | 14.2 | 245.0 | 78.9 | -15 | -44 | -37 |
| 152 | cytidine 5'-triphosphate | 15.4 | 482.2 | 158.8 | -85 | -36 | -9 |
| 153 | 4-diphosphocytidyl-2-C-methyl-*D*-erythritol 2-phosphate | 15.5 | 600.0 | 78.9 | -115 | -126 | -19 |
| 154 | desoxyadenosintriphosphat | 15.5 | 490.0 | 391.9 | -90 | -34 | -25 |
| 155 | sedoheptulose 1,7-bisphosphate | 15.5 | 369.0 | 97.0 | -35 | -20 | -27 |
| 156 | adenosine triphosphate | 15.6 | 506.2 | 158.8 | -80 | -38 | -9 |
| 157 | fructose-1,6-diphosphate | 15.7 | 339.0 | 96.9 | -35 | -22 | -11 |
| 158 | ribulose-1,5-bisphosphate | 15.7 | 309.0 | 97.0 | -35 | -20 | -27 |
| 159 | adenylosuccinic acid | 15.8 | 462.3 | 133.9 | -85 | -62 | -7 |
| 160 | orotidine 5'-monophosphate | 15.8 | 367.0 | 78.9 | -50 | -78 | -1 |
| 161 | mevalonate-5-diphosphate | 15.9 | 307.0 | 78.9 | -25 | -35 | -13 |
| 162 | guanosine-5'-triphosphate | 16.0 | 522.0 | 158.8 | -90 | -48 | -9 |
| 163 | deoxythymidine 5'-triphosphate | 16.0 | 481.0 | 158.7 | -80 | -38 | -9 |
| 164 | dihydronicotinamide adenine dinucleotide phosphate | 16.3 | 744.1 | 79.0 | -40 | -16 | -9 |
| 165 | ADP-ribose-2`-phosphate | 16.5 | 638.0 | 426.0 | -170 | -34 | -19 |
| 166 | coenzyme A | 17.0 | 766.1 | 407.9 | -245 | -50 | -19 |
| 167 | inositol triphosphate | 17.4 | 419.0 | 320.8 | -25 | -28 | -21 |
| 168 | 5-phosphoribosyl diphosphate | 16.8 | 388.9 | 176.8 | -55 | -28 | -9 |

| | | | | | | | |
|---|---|---|---|---|---|---|---|
| 169 | S-acetyl coenzyme A | 17.6 | 808.1 | 407.9 | -220 | -52 | -27 |
| 170 | methylmalonyl coenzyme A | 17.8 | 866.1 | 408.0 | -185 | -58 | -21 |
| 171 | geranyl diphosphate | 18.0 | 313.1 | 78.9 | -65 | -46 | -1 |
| 172 | β-hydroxy β-methylglutaryl-CoA | 18.5 | 910.1 | 407.9 | -220 | -52 | -27 |
| 173 | malonyl coenzyme A | 18.6 | 852.1 | 408.0 | -185 | -58 | -21 |
| 174 | ent-copal-8-ol diphosphate | 19.0 | 467.2 | 78.8 | -220 | -52 | -27 |
| 175 | succinyl coenzyme A | 19.0 | 866.1 | 407.6 | -260 | -56 | -25 |
| 176 | inositol-1,3,4,5-tetraphosphate | 19.2 | 498.3 | 400.7 | -100 | -30 | -27 |
| 177 | β-methylcrotonyl coenzyme A | 19.1 | 848.1 | 407.8 | -185 | -58 | -21 |
| 178 | 1-diphosinositol pentakisphosphate | 19.2 | 578.9 | 480.6 | -25 | -32 | -31 |
| 179 | geranylgeranyl pyrophosphate | 19.2 | 449.2 | 78.8 | -65 | -68 | -35 |
| 180 | isovaleryl coenzyme A | 19.2 | 850.2 | 407.9 | -240 | -58 | -19 |
| 181 | acetoacetyl coenzyme A | 19.5 | 580.1 | 408.0 | -220 | -52 | -27 |
| 182 | Phytic acid | 19.6 | 658.9 | 560.7 | -145 | -38 | -31 |
| 183 | farnesyl diphosphate | 19.7 | 381.1 | 78.9 | -50 | -50 | -5 |

The analysis relied Waters ACQUITY UPLC H-Class UPLC System (Waters GmbH, Eschborn, Germany) coupled online to a hybrid triple quadrupole-linear ion trap mass spectrometer (QqLIT) AB Sciex QTRAP 6500 (AB Sciex, Darmstadt, Germany).

**Table S1-10.** The conditions of ultrahigh performance liquid chromatographic (UHPLC) separation and the settings for electrospray ionization-quadrupole-time of flight mass spectrometry (ESI-QqTOF-MS) applied for the analysis of semi-polar secondary metabolites from *Cucumis sativus* root.

| **Chromatography** | |
|---|---|
| **SIL-30AC Autosampler** | |
| Injection mode | Partial Loop |
| Injection volume | 5 μL |
| Wash solvent | 50% MeOH |
| Wash solvent volume | 300 μL |
| Cooler temperature | 4.0 C |
| Rinse type | Internal & external |
| Rinse mode | Before and after aspiration |
| Needle overfill flush | Rinse port + rinse pump |
| Rinse time | 2 sec |
| **Column conditions** | |
| Separation column | ACQUITY UPLC BEH C18 Column (50 x 2.1 mm, particle size 1.7 μm) |
| Column oven temperature | 40.0 C |
| **LC separation parameters** | |
| Eluent A | 0.3 mmol/L aq. ammonium formate |
| Eluent B | acetonitrile |
| Flow rate | 0.4 mL/min |
| Elution program | 5% eluent B isocratic - 1 min<br>gradient to 95% eluent B – 6 min<br>95% eluent B isocratic – 2.5 min<br>gradient to 5% eluent B – 0.5 min<br>5% eluent B isocratic – 2.5 min (re-equilibration) |
| **Mass spectrometry** | |

| | |
|---|---|
| **General** | |
| Mass analyzer type | quadrupole-time of flight (QqTOF-MS) |
| Ionsource | ESI |
| Experiment type | Sequential Windowed Acquisition of All Theoretical Fragment Ion Mass Spectra (DIA) |
| Operatinon mode | positive, negative |
| Cycle time (s) | 1.108 |
| Duration | 6.5 min |
| **Ion source settings** | |
| Nebulizer gas (L/min) | 3 |
| Drying gas (L/min) | 10 |
| Ion spray voltage (kV) | 4.0/-3.0 (positive/negative mode) |
| Ion source temperature (°C) | 210 |
| **MS settings** | |
| Experiment type | TOF-MS |
| *m/z* range | 65 - 1250 |
| Accumulation time (ms) | 100 |
| ID function | ON |
| **MS/MS Settings** | |
| Collision gas | Ar |
| MS/MS experiment type | DIA |
| SWATH window number | 48 |
| SWATH window width (*m/z*) | 24.7 |
| CE | 10-80 (45±35V) |
| Accumulation time (ms) | 21 |

| | |
|---|---|
| ID function | OFF |
| Collision potential (V) | 45/-45 (positive/negative mode) |
| Collision energy spread (V) | 35 (positive/negative mode) |

The analysis relied on the high-performance liquid chromatograph coupled on line to a QqTOF mass spectrometer Shimadzu LCMS-9030 System (Shimadzu, Kyoto, Japan).

**Table S1-11.** Parameters of the nanoHPLC separation method employed in the nanoLC-QqTOF-MS-based proteomics experiments.

| **Parameter** | **Settings** | |
|---|---|---|
| Method parameters | | |
| Injection volume | 2 μL | |
| Injection mode | sample loading pressure 217.5 bar | |
| Column temperature | 45$^0$C | |
| Eluents | | |
| Solvent A | 0.1% (v/v) aq. formic acid | |
| Solvent B | 0.1% (v/v) formic acid in acetonitrile | |
| Elution regimen | Time (min) | % B |
| | 0 | 2 |
| | 40 | 40 |
| | 40,5 | 85 |
| | 55,9 | 85 |
| | 57,9 | 2 |
| | 60 | 2 |
| Trap Column | Thermo Trap Cartridge 5mm | |
| Volume | 0.148 μL | |
| Equilibration pressure | 217.5 bar | |
| Estimated equilibration time | 0.65 min | |
| Equilibration volume (×10) | 1.48 μL | |
| Separation Column | Bruker FORTY | |
| Volume | 0.742 μL | |
| Equilibration pressure | 600.0 bar | |
| Estimated equilibration time | 7.91 min | |
| Equilibration volume (×4) | 2.97 μL | |

**Table S1-12.** Instrument settings applied for ESI-QqTOF-MS DDA experiments employed in the nanoLC-QqTOF-MS-based proteomics experiments.

| Parameter | Settings |
|---|---|
| MS conditions | |
| Ionization mode | Positive |
| Mass to charge ratio (*m/z*) range | 150 – 2200 |
| Spectra rate | 2 Hz |
| End plate offset | 500 V |
| Capillary voltage | 4500 V |
| Nebulizer | 1.5 bar |
| Dry temperature | 200°C |
| Dry gas | 2.0 l/min |
| MS/MS conditions | |
| Scan mode | Auto MS/MS |
| Fragmentation type | Collision-induced dissociation |
| Isolation width | 2 – 3 |
| MS/MS spectra acquisition | 8 – 32 Hz |
| Threshold (per 1000 sum.) | 250 cts |
| Cycle time | 3 sec |
| Collision energy | from 23 eV (m/z 300) to 65 eV (m/z 1300) |
| Scan mode | Auto MS/MS |

**Table S1-13.** PEAKS Studio 10.6 parameters for database search settings.

| Database search settings | |
|---|---|
| Analysis program | PEAKS Studio 10.6 build 20201221 |
| Parent mass error tolerance: | 10.0 ppm |
| Fragment mass error tolerance: | 0.05 Da |
| Precursor mass search type: | Monoisotopic |
| Protease | Trypsin |
| Missed cleavage sites | 2 |
| FDR | 2 |
| Fixed modifications: | Carbamidomethylation: 57.02 |
| Variable modifications | Oxidation (M): 15.99 |
| | Acetylation (Protein N-term): 42.01 |
| | Deamidation (NQ): 0.98 |
| Max variable PTM per peptide | 2 |
| Filter charge | 1 – 7 |

# Figures

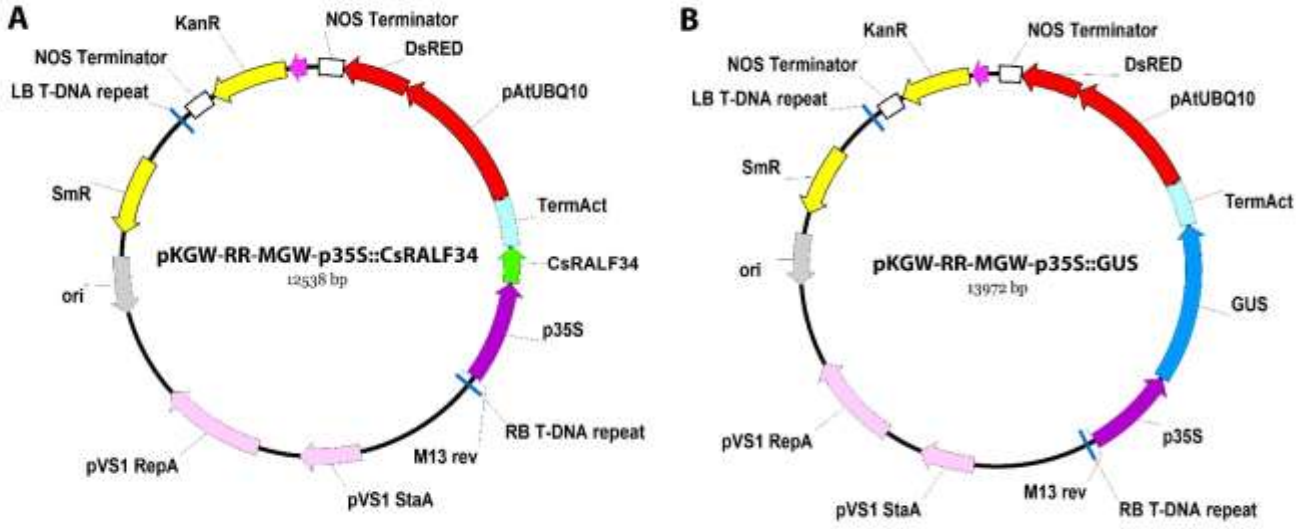


**Figure S1-1.** Map of vectors for *CsRALF34* overexpression.

Maps of binary vectors (A) pKGW-RR-MGW-*p35S::CsRALF34* and (B) pKGW-RR-MGW-*p35S::gusA* used for *R. rhizogenes*-mediated transformation of cucumber seedlings. Both vectors (A,B) contain DsRED1 under the control of the *AtUBQ10* promoter as a screenable marker within the T-DNA borders. (A) pKGW-RR-MGW-*p35S::CsRALF34* vector contains *p35S::CsRALF34* fusion for *CsRALF34* overexpression and (B) pKGW-RR-MGW-*p35S::gusA* contains *p35::gusA* fusion as a control against *CsRALF34* overexpression.

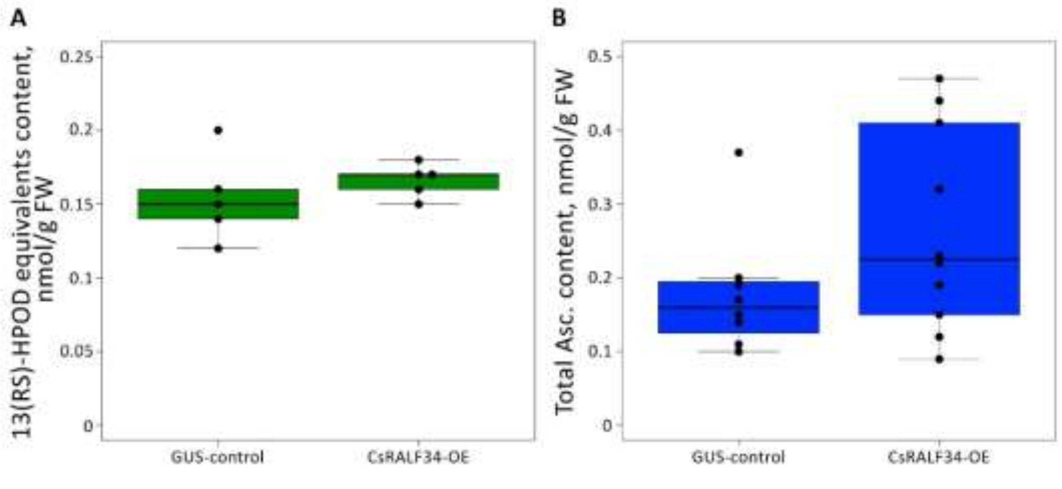


**Figure S1-2.** Biochemical characterization of *Cucumis sativus* transgenic roots overexpressing *CsRALF34*.

Graphs were drawn using R software default code for boxplot and stripchart functions. (**A**) The contents of hydroperoxides (expressed as 13S-hydroperoxy-9Z,11E-octadecanoic acid (13(RS)-HPOD) equivalents) in GUS-control roots (overexpression of *β*-glucuronidase gene) and with *CsRALF34* overexpression (RALF34-OE). (**B**) Total ascorbate (Asc) contents in GUS-control and in RALF34-OE. Statistical analysis using Student's t-test showed no significant differences ($p > 0.05$) in *CsRALF34* overexpression group compared to the GUS-control. The raw data are presented in Supplementary information 2.

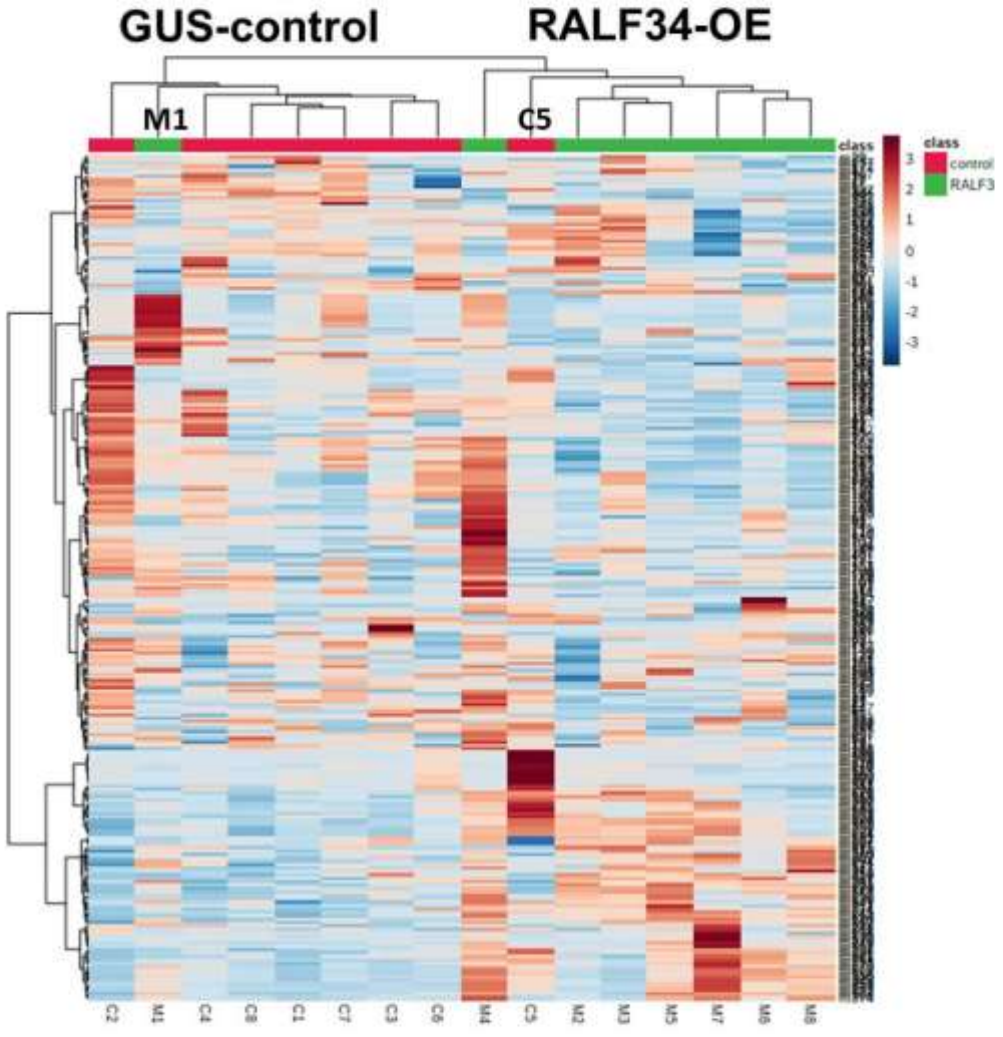


**Figure S1-3.** Results of hierarchical clustering with a heatmap representation of all primary metabolites.

Annotated with GC-EI-Q-MS and RP-IP-UHPLC-QqQ-MS/MS after merging together both result sets and processing the resulted data matrix by Metaboanalyst online tool. Based on the results of hierarchical clustering, the samples M1 and C5 can be treated as the outliers and were, therefore, excluded from the further analysis.

**A**

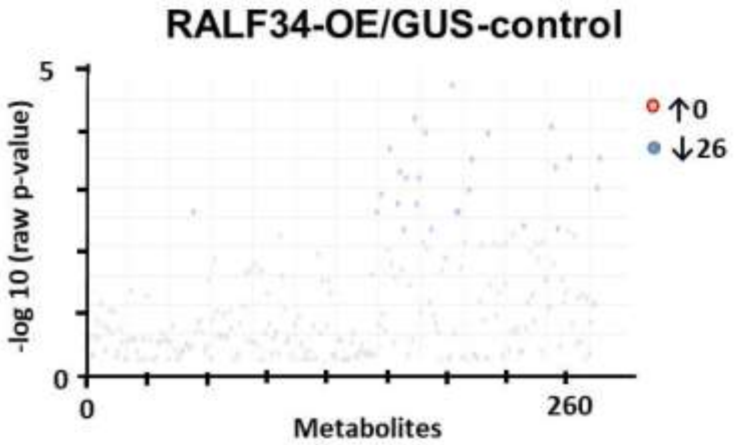


**B**

| significan tdown metabolites | t.stat | p.value | -log10(p) | FDR |
|---|---|---|---|---|
| serine | -6.857 | 0.000 | 4.756 | 0.004 |
| glycerophosphoric acid | -5.975 | 0.000 | 4.190 | 0.006 |
| nicotinic acid | -5.766 | 0.000 | 4.049 | 0.006 |
| isocitric acid | -5.612 | 0.000 | 3.943 | 0.006 |
| citric acid | -5.225 | 0.000 | 3.671 | 0.009 |
| sedoheptulose 7-phosphate | -4.999 | 0.000 | 3.509 | 0.009 |
| arginosuccinat | -4.999 | 0.000 | 3.509 | 0.009 |
| adenylosuccinic acid | -4.971 | 0.000 | 3.488 | 0.009 |
| ornithine | -4.788 | 0.000 | 3.354 | 0.011 |
| fructose 6-phosphate | -4.682 | 0.001 | 3.275 | 0.012 |
| glycine | -4.545 | 0.001 | 3.173 | 0.013 |
| glucose-6-phosphate | -4.539 | 0.001 | 3.168 | 0.013 |
| glucose | -4.310 | 0.001 | 2.994 | 0.018 |
| aconitic acid | -4.270 | 0.001 | 2.964 | 0.018 |
| arginine | -4.173 | 0.001 | 2.888 | 0.020 |
| erythrose 4-phosphate | -3.971 | 0.002 | 2.731 | 0.026 |
| glutamine | -3.962 | 0.002 | 2.725 | 0.026 |
| threonine | -3.801 | 0.003 | 2.597 | 0.029 |
| sucrose | -3.787 | 0.003 | 2.587 | 0.029 |
| 6-phosphogluconic acid | -3.784 | 0.003 | 2.584 | 0.029 |
| orotidine 5'-monophosphate | -3.430 | 0.005 | 2.302 | 0.049 |
| malic acid | -3.418 | 0.005 | 2.293 | 0.049 |
| glucose-1-phosphate | -3.408 | 0.005 | 2.285 | 0.049 |
| gluconic/galacturonic acid | 3.491 | 0.004 | 2.351 | 0.047 |
| *D*-mannose | 3.790 | 0.003 | 2.589 | 0.029 |
| cGMP | 5.595 | 0.000 | 3.932 | 0.006 |

**Figure S1-4.** Visualization of the results (A) and the corresponding statistical information (B) acquired in the t-test accomplished for the aqueous methanolic extracts obtained from the *Cucumis sativus* roots with *CsRALF34* overexpression or from the control group.

A

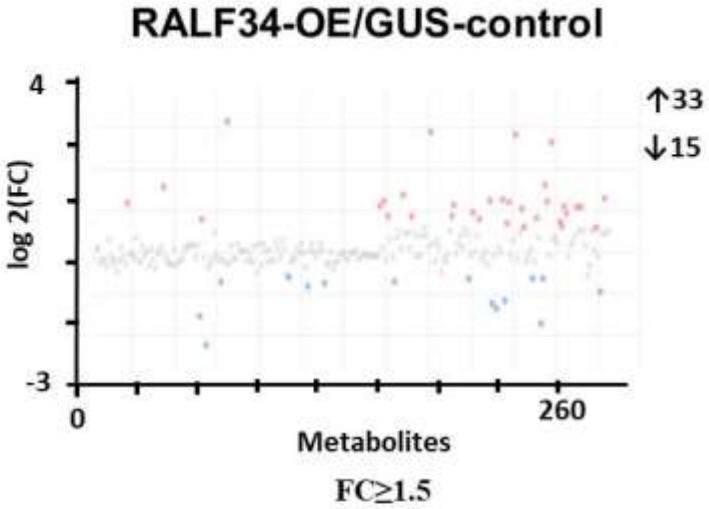


B

| | Significant changed metabolites | Fold Change | log2(FC) |
|---|---|---|---|
| significant down | 5-keto-*D*-gluconic acid | 0.213 | -2.231 |
| | glutathione | 0.306 | -1.710 |
| | *D*-mannose | 0.343 | -1.542 |
| | cGMP | 0.391 | -1.356 |
| | ATP | 0.425 | -1.235 |
| | CTP | 0.447 | -1.161 |
| | UTP | 0.519 | -0.947 |
| | RI_2785_unknwon_disacharide | 0.569 | -0.814 |
| | melibiose | 0.598 | -0.742 |
| | 1,2,3,4,6-*O*-glucopyranose, 5TMS | 0.610 | -0.714 |
| | ureidosuccinic acid | 0.614 | -0.704 |
| | ascorbic acid | 0.643 | -0.638 |
| | glutathione disulfide | 0.645 | -0.633 |
| | gluconic/galacturonic acid | 0.648 | -0.625 |
| | sucrose | 0.658 | -0.603 |

| | Significant changed metabolites | Fold Change | log2(FC) |
|---|---|---|---|
| significant up | **UMP** | 1.507 | 0.592 |
| | **fructose-1,6-diphosphate** | 1.526 | 0.609 |
| | **NAD+** | 1.538 | 0.621 |
| | **dADP** | 1.620 | 0.696 |
| | **nicotinic acid** | 1.665 | 0.735 |
| | **decanedioic acid, 2TMS** | 1.736 | 0.796 |
| | **adenosine** | 1.745 | 0.803 |
| | **glycolate** | 1.766 | 0.821 |
| | **glucose-1-phosphate** | 1.813 | 0.858 |
| | **arginine** | 1.825 | 0.868 |
| | **ribose-1-phosphate** | 1.834 | 0.875 |
| | **orotidine 5'-monophosphate** | 1.916 | 0.938 |
| | **3-phosphoglyceric acid** | 1.941 | 0.957 |
| | **1-deoxy-*D*-xylulose 5-phosphate** | 2.069 | 1.049 |
| | **ribonic acid** | 2.128 | 1.089 |
| | **shikimic acid** | 2.130 | 1.091 |
| | **ornithine** | 2.151 | 1.105 |
| | **2-phosphoglyceric acid** | 2.157 | 1.109 |
| | **2-deoxyribose 5-phosphate** | 2.202 | 1.139 |
| | **serine, 2TMS** | 2.277 | 1.187 |
| | **dGMP** | 2.301 | 1.202 |
| | **inosinic acid** | 2.328 | 1.219 |
| | **6-phosphogluconic acid** | 2.346 | 1.230 |
| | **AMP** | 2.364 | 1.241 |
| | **CMP** | 2.401 | 1.264 |
| | **xanthosine-5'-phosphate** | 2.456 | 1.296 |
| | **2-deoxyribose 5-phosphate** | 2.596 | 1.376 |
| | **2-phenylglycine** | 2.974 | 1.573 |
| | **histidine** | 3.055 | 1.611 |
| | **delta3-isopentenyl pyrophosphate** | 6.249 | 2.644 |
| | **dimethylallylpyrophosphat** | 7.109 | 2.830 |
| | **isopentenyl pyrophosphate** | 7.439 | 2.895 |
| | ***N*-*α*-acetyl-*L*-lysine** | 8.881 | 3.151 |

**Figure S1-5.** Visualization of the results (A) and the corresponding statistical information (B) acquired in the fold-change (FC) analysis with the cut-off FC ≥ 1.5, accomplished for the aqueous methanolic extracts obtained from the *Cucumis sativus* roots with *CsRALF34* overexpression or from the control group.

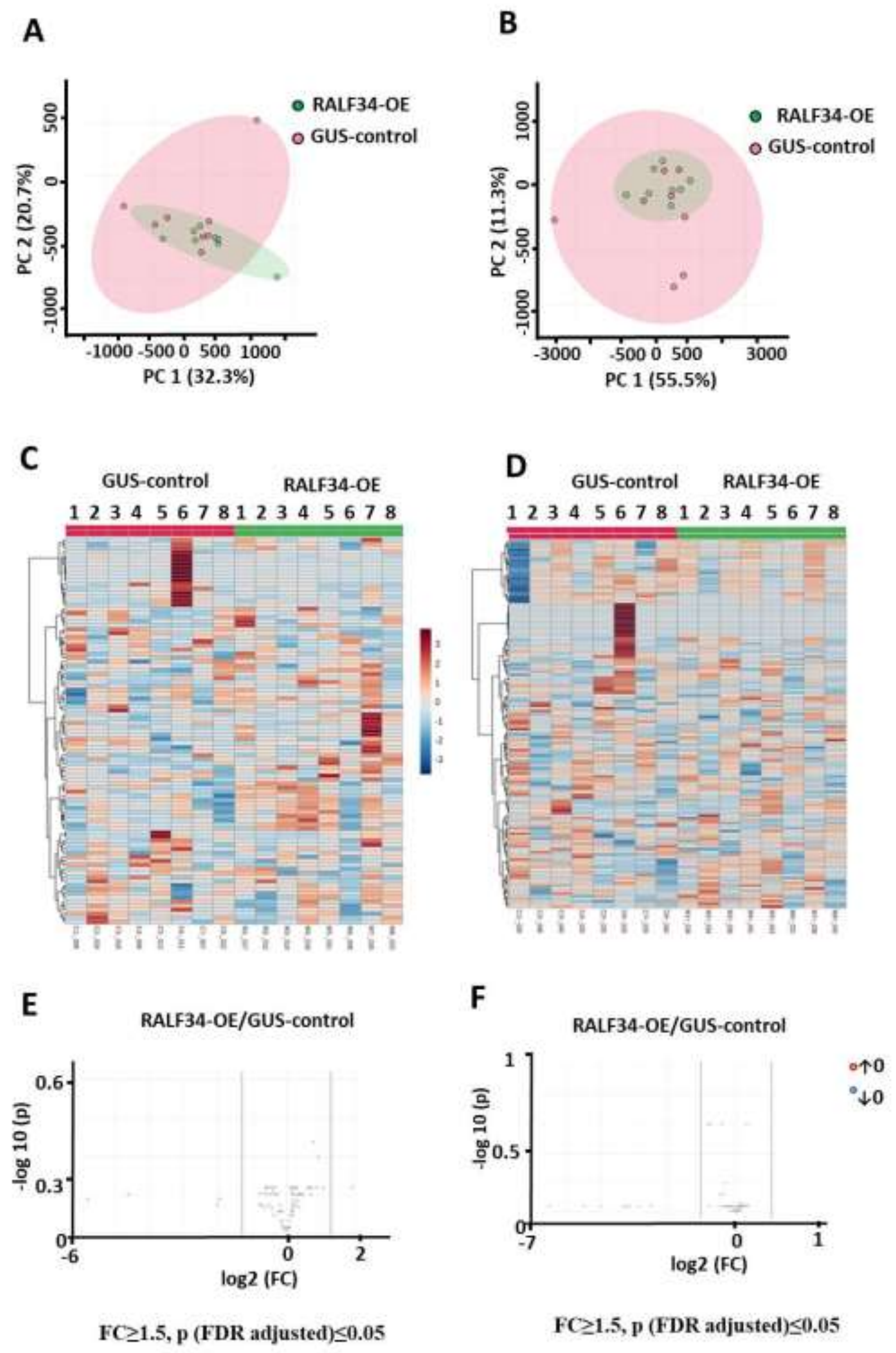


**Figure S1-6.** Analysis of the semi-polar secondary metabolites: post-processing of the RP-HPLC-QqTOF-MS data.

The negative (A, C and E) and positive (B, D and F) ion mode with principal component analysis, PCA (score plots, A and B), hierarchical clustering with the heatmap representation (C and D) and t-test visualization (Volcano plots, E and F).

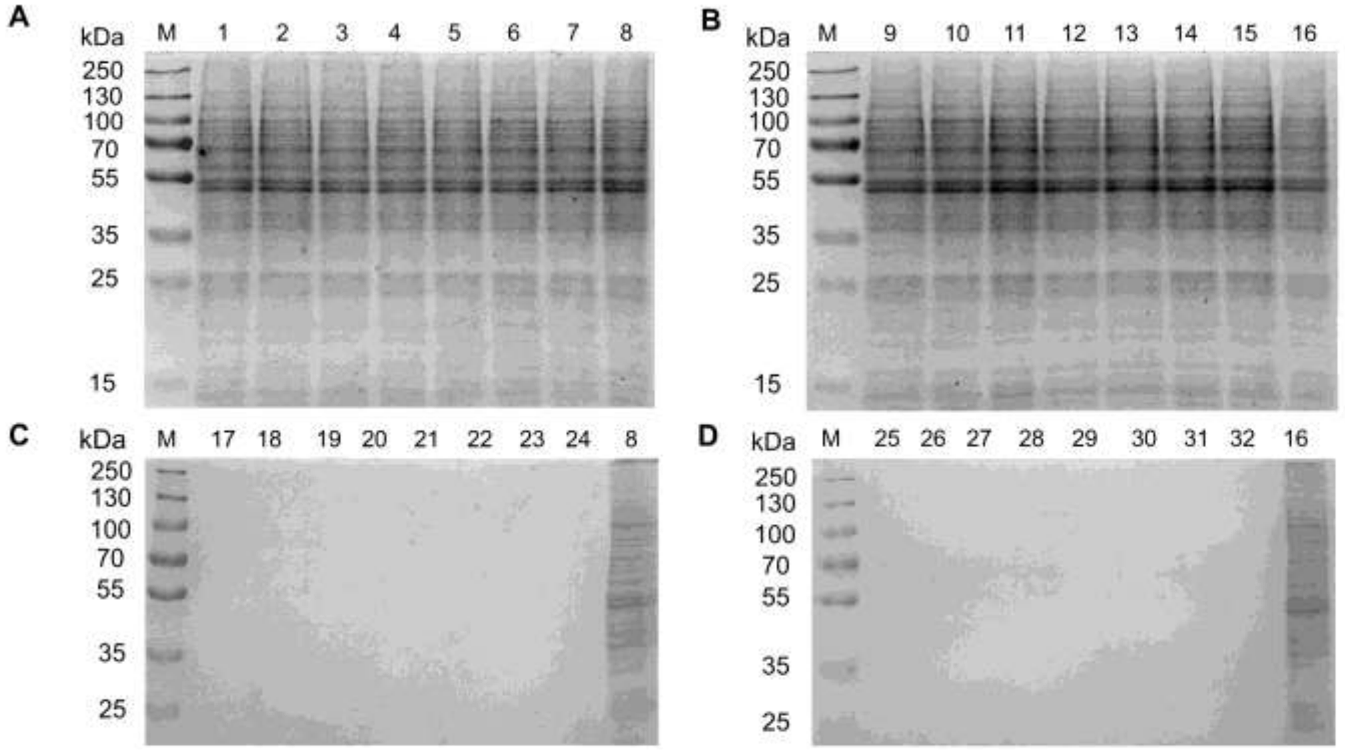


**Figure S1-7.** Electrophoregrams of total protein isolates and their tryptic hydrolysates from the *Cucumis sativus* roots from the control group (GUS-control) and with *CsRALF34* overexpression (CsRALF34-OE).

Isolates from 5 μg of total protein (panels A and B, lanes 1-16) and their tryptic hydrolysates (panels C and D, lanes 17-32) for control plants (A, C) and *CsRALF34* overexpression (B, D). M – marker of molecular weight (PageRuller Plus Prestained Protein Ladder, 10–250 kDa, Thermo Fisher Scientific). The gels were stained with 0.1% (w/v) Coomassie G-250 solution.

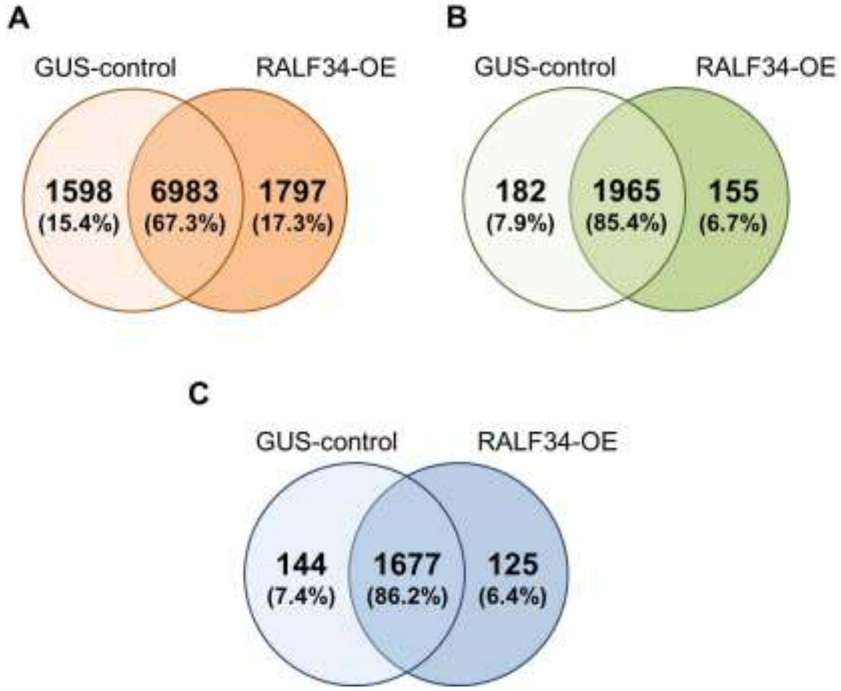


**Figure S1-8.** Number of peptides (A), proteins (B) and protein groups (C), identified in control (GUS-control) and CsRALF34-OE (overexpression of *CsRALF34*) *Cucumis sativus* roots.

Identification and annotation of peptides and proteins relied on *Cucumis sativus* sequence database in PEAKS Studio 10.6 software.

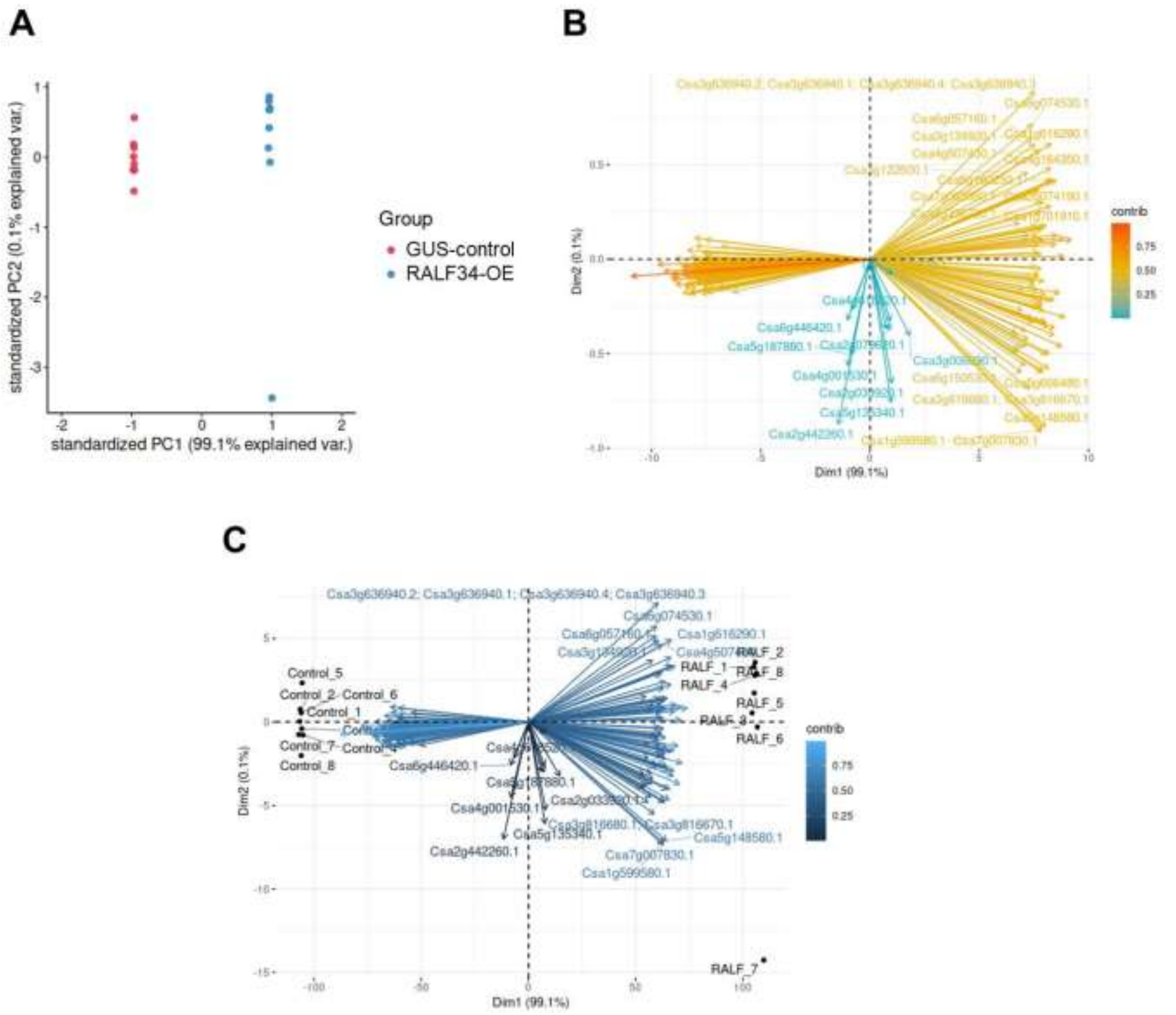


**Figure S1-9.** Principal component analysis (PCA) with a score plot (A), loadings plot (B) and a biplot illustrating correlation between principal components and source variables (C).

The complete list of the eigenvalues (loadings) for all differentially expressed proteins is provided in Table S1-4.

## Protocols

**Protocol S1-1.** The full list of reagents.

Unless stated otherwise, materials were obtained from the following manufacturers. AMRESCO LLC (Cleveland, Ohio, USA): bis-acrylamide (ultra pure grade), tris-(2-carboxyethyl)-phosphine hydrochloride (ultra pure grade), 2-mercaptoethanol (biotechnology grade), phenylmethyl sulfonyl fluoride (high purity grade); Bioanalytical Technologies 3M Company (Saint Paul, Minnesota, USA): Empore™ solid phase octodecyl extraction discs; Calbiochem (Madison, Wisconsin, USA): glycine (molecular biology grade); Carl Roth Gmbh (Karlsruhe, Germany): glacial acetic acid (ROTIPURAN®, p.a. 100%); Dia-M (Moscow, Russia): phenol for molecular biology (99.5%); Ekos-1 (Moscow, Russia): acetone (extra pure); Helicon (Moscow, Russia): *N,N,N′,N′*-tetramethylethylenediamine (ultra pure), ammonium persulfate (ACS grade), sodium dodecyl sulfate (biotechnology grade), acrylamide 2K (standard grade, extra pure), Urea (USP grade), polyoxyethilene-20-sorbitan monolaurate (reagent grade), DL-dithiothreitol (biotechnology grade), potassium chloride (CP); LSU Research Institute (Saint Petersburg, Russia): chloroform; Merck KGaA (Darmstadt, Germany): acetonitrile (LC-MS grade), ethanol (LC grade), methanol (LC-MS grade); Reachem (Moscow, Russia): hydrochloric acid (GR for analysis), trichloroacetic acid, sodium carbonate (extra pure); SERVA Electrophoresis GmbH (Heidelberg, Germany): Coomassie Brilliant Blue G-250 (ultra pure), 2-mercaptoethanol (research grade), trypsin NB (sequencing grade, modified from porcine pancreas); Thermo Fisher Scientific (Waltham, Massachusetts, USA): PageRuller™ Plus Prestained Protein Ladder #26619 (10–250 kDa); Dichrom GmbH (Marl, Germany): Progenta™ adaptors for stage-tips; Sigma-Aldrich (Saint Louis, Missouri, USA): ammonium formate (for LC-MS, ≥ 99.0%), ascorbate oxidase from *Cucurbita sp.*, catalase from bovine liver, ethylenediaminetetraacetic acid, iodoacetamide (BioUltra), ammonium bicarbonate (≥ 99.0%), formic acid (≥ 98%) , xylenol orange tetrasodium salt (ACS grade), ammonium acetate (≥ 98%), sorbitol (GR for analysis), tributylamine (≥ 99%); Vekton (St. Petersburg, Russia): thiobarbituric acid (≥ 99%). Water was purified in-house (resistance 5–15 mΩ/cm) on water conditioning and purification systems Elix 3 UV (Millipore, Moscow, Russia) or Millipore Milli-Q Gradient A10 system (resistance 5–15 mΩ/cm, Merck Millipore, Darmstadt, Germany).

**Protocol S1-2.** Determination of hydrogen peroxide contents.

Analysis of $H_2O_2$ contents in cucumber roots relied on the method described by Chantseva, *et al.* [1] with modifications. In detail, 100 mg of frozen milled plant material were supplemented with 1 mL of ice-cold $HClO_4$ (0.4 mol/L), vortexed intensively (30 s) and centrifuged (13000x g, 10 min, 4°C). Then the supernatants were neutralized by 4 mol/L potassium hydroxide to achieve pH = 7 with sub-sequent centrifugation (13000x g, 10 min, 4°C). Next, 375 µL of sodium phosphate buffer (0.1 mol/L, pH 5.6), 1 UN of ascorbate oxidase (1.5 µL) and 125 µL of each sample extract were added to two UV grade cuvettes, and the mixtures were vortexed for 30 s. Afterwards, 1 µL of catalase solution (1 UN) was added to one of the cuvettes with further incubation (2 min, 25°C). Finally, 500 µL aliquots of FOX solution (0.2 mmol/L xylenol orange tetrasodium salt, 200 mmol/L sorbitol, 50 mmol/L sulphuric acid, 0.5 mmol/L ammonium ferrous sulphate hexahydrate) were added to both cuvettes (with and without supplemented catalase). The cuvettes were vortexed (30 s) and left in the dark for 30 min, after what the absorbance was measured at 575 nm (the length of the optical way – 1 cm) and calculations were accomplished as described previously [1].

**Protocol S1-3.** Determination of lipid peroxidation product contents.

Lipid peroxidation products were quantified as malondialdehyde equivalents according to the protocol described by Soboleva, *et al.* [2] with modifications. In detail, approximately 25 mg of frozen milled plant material were left on ice for 3 minutes, before addition of 300 µL 5% (w/v) trichloroacetic acid, the resulted suspensions were vortexed for 30 s and centrifuged (10000 x g, 20 min, 4°C). Afterwards, 1 mL of thiobarbituric acid (TBA) reagent (0.5% w/v TBA in 20% trichloroacetic acid) were supplemented to 250 µL of supernatant. The mixtures were incubated (30 min, 95°C). Afterwards, the mixtures were cooled on ice to stop the reaction, centrifuged (1900 x g, 10 min, 4°C) and 1 mL of each coloured supernatant was used to measure the absorbance at 532 nm against the proper blank (250 µL 5% w/v trichloroacetic acid and 750 µL TBA reagent). The non-specific absorbance at 600 nm was

subtracted from the absorbance acquired at 532 nm. The contents of malondialdehyde were calculated with ε = 155 $mM^{-1}cm^{-1}$. The length of optical way – 1 cm.

**Protocol S1-4.** Determination of lipid hydroperoxide contents.

Analysis of lipid hydroperoxide contents relied on the method of Frolov, *et al.* [3] with modifications. In detail, 10 mg of frozen milled plant material were transferred on ice and 3 min later supplemented with 750 µL of ice-cold chloroform-methanol mixture (ratio 1:2, v/v) and 150 µL of aquatiq acetic acid (0.15 mol/L), then vortexed for 30 s. The next step was addition of 225 µL of chloroform with 0.01% (w/v) butylated hydroxytoluene (BHT) and the same volume of distillated water, vortexing (30 s) and centrifugation (3000x g, 5 min, 4°C). Afterwards, the lower phase was collected, transferred to black polypropylene tubes and dried under reduced pressure (CentriVap Vacuum Concentrator, Labconco, Kansas City, Missouri, USA) for 120 min. After this, 100 µL of 0.01% (w/v) BHT in methanol was added and tubes were left on ice for 30 min. Further addition of 900 µL of ferrous ion oxidation xylenol orange (1.0 mmol/L xylenol orange and 2.5 mmol/L ammonium ferrous sulphate in 250 mmol/L $H_2SO_4$ – 0.01% (w/v) BHT in methanol, 1:9, v/v) was also accompanied by 30 min incubation on ice and, afterwards, the absorption at 560 nm (the length of the optical way – 1 cm) against pure FOX reagent was assessed. The hydroperoxide contents were calculated as 13S-hydroperoxy-9Z, 11E-octadecanoic acid equivalents, ε = 6.0 x $10^4$ $M^{-1}cm^{-1}$.

**Protocol S1-5.** Determination of ascorbic acid contents.

Determination of the ascorbate contents relied on the method described by Shumilina, *et al.* [4] with modifications. In detail, 50 mg of frozen milled plant material were supplemented with 500 µL of ice-cold $HClO_4$ (2.5 mol/L), were vortexed intensively for 30 s and centrifuged (13000 x g, 10 min, 4°C). Afterwards, the supernatants were neutralized by saturated solution of $Na_2CO_3$ to achieve pH 7.0 with further centrifugation (13000x g, 10 min, 4°C). To assess the contents of reduced ascorbate (ascorbic acid), 1.8 µL of sodium phosphate buffer (0.1 mol/L, pH 5.6) and 200 µl of each supernatant were supplemented to a UV grade cuvette. The suspensions were mixed by the vortex for 30 s, and the optical density (A1) of the samples was measured at 265 nm . Next, 4 µL of ascorbate oxidase (0.5 e.a./µl) was added and the samples were vortexed (30 s). After 5 min incubation, the optical density ($A_2$) was measured again at 265 nm. To assess the total ascorbate contents, 3.3 µL of dithiothreitol (DTT) were added to 250 µL of supernatant, vortexed (30 s) and incubated (15 min, 25°C). Then, 1.8 µL of sodium phosphate buffer (0.1 mol/L, pH 5.6) was added to 200 µL of each reduced sample, vortexed (30 s) and the optical density ($A_3$) at 265 nm was assessed. The contents of oxidized ascorbate (dehydroascorbic acid) were calculated as the difference between the values obtained for the total and reduced ascorbate. The statistical calculations were done as described previously [4]. The length of the optical way – 1 cm.

**Protocol S1-6.** Analysis of Primary Metabolites.

Analysis of the temperature-stable polar primary metabolites relied on water-methanol extraction of frozen ground cucumber roots (45 ± 10 mg) as described by Chantseva, Bilova, Smolikova, Frolov and Medvedev [1] with minor modifications. Aliquots (100 µL) of the extracts (total extract volume was 1100 µL) were freeze-dried overnight. The aliquot volumes were adjusted in a series of preliminary optimization experiments to 0.5, 1, 2, 4, 8, 16, 31, 63 and 125 µL of a pooled root extract (n = 3). The residues were sequentially derivatized with methoxyamine hydrochloride (MOA) and N-methyl-N-(trimethylsilyl)trifluoroacetamide (MSTFA) according to the earlier established procedure [1]. The samples (1 µL) were analyzed by GC-EI-Q-MS using a GCMS-QP2010 Ultra (Shimadzu, Kyoto, Japan), equipped with an auto sampler AOC-5000 Plus (Shimadzu) under the instrumental settings summarized in Table S1-8. Prior to the analysis, the samples were randomized with quality controls (QCs –aliquots of the pool prepared from equal volumes of all extracts) injected after each four samples.

Analysis of temperature-labile anionic primary metabolites was accomplished according to n integrated protocol including isolation of both polar primary and semi-polar secondary compounds. The applied procedure relied on the three-step extraction procedure described by Leonova, *et al.* [5] with minor modifications. Approximately 100 ± 5 mg of frozen ground root material were placed in 2 mL microtubes filled with 200 mg glass beads (0.75–1 mm diameter), 3 and 1 stainless steel beads of 3 mm and 5 mm diameter, respectively, and supplemented with 900 µL of cold (-80°C) dichloromethane (DCM)/ethanol mixture (2:1, v/v) with addition of 100 µL of ice-cold 1:200 HCl:water. After intensive homogenization (Mixer Mill MM 400 ball mill, Retsch, Haan, Germany, 30 Hz, 2x 30 s) and centrifugation (4°C, 10 000 g, 5 min), 300 µL of the polar supernatant fraction were

transferred into new pre-cooled 1.5 mL polypropylene microtubes. The residues were supplemented with 50 µL of ice-cold 1:200 HCl:water (v/v) mixture. The samples were mixed and centrifuged as described above, before 120 µL of the polar supernatant fractions were combined with the first portions and lyophilized. Then 180 µL of cold EtOH:water (3:1, v/v) solution were added into each sample and shaken at 1900 rpm (i.e., the maximum intensity) for 5 min at 4°C. In the next step, the samples were sonicated (3 min), and again shaken at 1900 rpm for 5 min at 4°C. This treatment was repeated twice more. Then the samples were centrifuged for 5 min at 12 000 x g, 4°C. The samples were analyzed with the above described randomization/standardization strategy by RP-IP-UHPLC-ESI-QqQ-MS/MS using Waters ACQUITY H-Class UPLC System (Waters GmbH, Eschborn, Germany), coupled on-line to an AB Sciex QTRAP 6500 LC-MS/MS System (AB Sciex, Darmstadt, Germany) under the chromatographic and mass spectrometric settings summarized in Supplementary information 1 (Table S1-9).

**Protocol S1-7.** Analysis of Semi-Polar Secondary Metabolites.

To the non-polar supernatant fractions (700 µL) were added 500 µL cold (-80°C) tetrahydrofuran. After mixing (1900 rpm, 5 min, 4°C) and centrifugation (4°C, 10 000 g, 5 min) the non-polar supernatant fraction (500 µL) was combined with the first portion and gently dried under reduced pressure at 4°C in a Labconco CentriVap vacuum concentrator (Labconco, Kansas City, Missouri, USA). The residues were reconstituted in 180 µL of a water-methanol mixture (1:3, v/v), vortexed (3000 g, 15 s), centrifuged (4°C, 10000 g, 10 min), and the supernatants were filtered as described above. The samples were analyzed with the above described randomization/standardization strategy by reversed phase-high performance liquid chromatography-electrospray ionization-quadrupole-time-of-flight mass spectrometry (RP-HPLC-ESI-QqTOF-MS) using liquid chromatograph-mass spectrometer S LCMS-9030 System (Shimadzu), operated in positive or negative ion mode. Data acquisition relied on the sequential window acquisition of all theoretical mass spectra (SWATH) mode.

**Protocol S1-8.** Protein isolation and determination.

The protein extraction was accomplished with approximately 250 mg of milled frozen root material by relied on the method of Frolov, *et al.* [6] with some modifications. The 700 µL of cold (4°C) phenol extraction buffer (0.7 mol/L sucrose, 0.1 mol/L KCl, 5 mmol/L ethylenediaminetetraacetic acid, 2% (v/v) β-mercaptoethanol and 1 mmol/L phenylmethylsulfonyl fluoride in 0.5 mol/L Tris-HCl buffer, pH 7.5) was added to the plant material. The suspensions were vortexed for 30 s. Then 700 µL of cold phenol (4°C) preliminarily saturated with 0.5 mol/L Tris-HCl buffer (pH 7.5) were added. After further mixing for 30 s, the samples were shaken (30 min, 900 rpm, 4°C) and centrifuged (5000x g, 15 min, 4°C). Afterwards, the phenolic (upper) phase was washed two times with equal volumes of the phenol extraction buffer (after each buffer addition: vortexing 30 s, shaking for 30 min at 900 rpm at 4°C, and centrifugation at 5000x g for 15 min at 4°C). Then, the proteins were precipitated by addition of 1 mL of ice-cold (–20°C) ammonium acetate in methanol (0.1 mol/L), followed by storage overnight at −20°C. Next morning, the protein fraction was collected by centrifugation (5000x g, 10 min, 4°C), and the supernatants were discarded. The pellets were washed twice with two volumes of methanol (relative to the volume of the phenol phase), and twice with the same volume of acetone (both at 4°C). Each time after re-suspending, the samples were centrifuged (5000 x g, 10 min, 4°C) and supernatants were discarded. Finally, the cleaned pellets were dried under air flow in a fume hood for 1 h and then re-constituted in 70 µL of 4% sodium dodecyl sulphate (SDS). The subsequent determination of protein concentrations was done by Bicinchoninic Acid Kit for Protein Determination (Sigma-Aldrich).

**Protocol S1-9.** Solid Phase Extraction.

The proteolytic hydrolysates were pre-cleaned by reversed phase solid phase extraction (RP-SPE) using the elution scheme of Spiller, *et al.* [7] with minor modifications. Stage-Tips with six layers of C-18 Extraction Disks (Sigma-Aldrich, 66883-U) were prepared in 200 µL polypropylene pipette tips and inserted in 2 mL tubes via plastic tube adaptors. The Stage-Tips were conditioned with 100 µL of MeOH (2000x g, 5 min, 25°C) and equilibrated with two portions of 200 µL of 0.1% (v/v) formic acid (FA, 2000x g, 5 min, 25°C), before the individual tryptic digests were applied and the stage tips were centrifuged (2000x g, 5 min, 25°C). After the washing (2 x 200 µL of 0.1% (v/v) FA) with centrifugation after each step (2000x g, 5 min, 25°C), the peptides were eluted by sequential application of 150 µL of 60% (v/v) acetonitrile and 150 µL of 80% (v/v) acetonitrile, 0.1% (v/v) FA. Finally, the pooled eluate was

transferred to 0.5 mL polypropylene tubes and dried (4°C) under the reduced pressure in the vacuum concentrator CentriVap Vacuum Concentrator (Labconco).

## References


1. Chantseva, V.; Bilova, T.; Smolikova, G.; Frolov, A.; Medvedev, S. 3D-clinorotation induces specific alterations in metabolite profiles of germinating *Brassica napus* L. seeds. *Biol. Commun.* **2019**, *64*, 55–74, doi:10.21638/spbu03.2019.107.
2. Soboleva, A.; Frolova, N.; Bureiko, K.; Shumilina, J.; Balcke, G.U.; Zhukov, V.A.; Tikhonovich, I.A.; Frolov, A. Dynamics of reactive carbonyl species in pea root nodules in response to polyethylene glycol (PEG)-induced osmotic stress. *Int. J. Mol. Sci.* **2022**, *23*, doi:10.3390/ijms23052726.
3. Frolov, A.; Bilova, T.; Paudel, G.; Berger, R.; Balcke, G.U.; Birkemeyer, C.; Wessjohann, L.A. Early responses of mature *Arabidopsis thaliana* plants to reduced water potential in the agar-based polyethylene glycol infusion drought model. *J. Plant Physiol.* **2017**, *208*, 70-83, doi:10.1016/j.jplph.2016.09.013.
4. Shumilina, J.; Gorbach, D.; Popova, V.; Tsarev, A.; Kuznetsova, A.; Grashina, M.; Dorn, M.; Lukasheva, E.; Osmolovskaya, N.; Romanovskaya, E., et al. Protein glycation and drought response of pea (*Pisum sativum* L.) root nodule proteome: a proteomics approach. *Biol. Commun.* **2021**, *66*, 210–224, doi:10.21638/spbu03.2021.303.
5. Leonova, T.; Popova, V.; Tsarev, A.; Henning, C.; Antonova, K.; Rogovskaya, N.; Vikhnina, M.; Baldensperger, T.; Soboleva, A.; Dinastia, E., et al. Does protein glycation impact on the drought-related changes in metabolism and nutritional properties of mature pea (*Pisum sativum* L.) seeds? *Int. J. Mol. Sci.* **2020**, *21*, 1-29, doi:10.3390/ijms21020567.
6. Frolov, A.; Didio, A.; Ihling, C.; Chantzeva, V.; Grishina, T.; Hoehenwarter, W.; Sinz, A.; Smolikova, G.; Bilova, T.; Medvedev, S. The effect of simulated microgravity on the *Brassica napus* seedling proteome. *Funct. Plant Biol.* **2018**, *45*, 440-452, doi:10.1071/FP16378.
7. Spiller, S.; Frolov, A.; Hoffmann, R. Quantification of specific glycation sites in human serum albumin as prospective type 2 diabetes mellitus biomarkers. *Protein Peptide Lett.* **2017**, *24*, 887-896, doi:10.2174/0929866524666170202124120.
